# Combining sampling and attractor dynamics in spiking models of head direction systems

**DOI:** 10.1371/journal.pcbi.1014577

**Published:** 2026-07-30

**Authors:** Vojko Pjanovic, Jacob A. Zavatone-Veth, Paul Masset, Sander W. Keemink, Michele Nardin

**Affiliations:** 1 Janelia Research Campus, Howard Hughes Medical Institute, Ashburn, Virginia, United States of America; 2 Department of Machine Learning and Neural Computing, Donders Institute for Brain, Cognition and Behaviour, Radboud University, Nijmegen, Netherlands; 3 Society of Fellows and Center for Brain Science, Harvard University, Cambridge, Massachusetts, United States of America; 4 Department of Psychology, McGill University, Montréal, Québec, Canada; 5 Mila - Quebec Artificial Intelligence Institute, Montréal, Québec, Canada; Johns Hopkins University, UNITED STATES OF AMERICA

## Abstract

Neural populations can maintain stable representations of navigation-related variables while integrating uncertain sensory signals. Experimental evidence showed that the precision of head-direction (HD) representations in flies and mice depends on the reliability of sensory cues, highlighting the influence of input uncertainty in attractor-based neural circuits. How do neural dynamics maintain stability while computing under uncertainty? Here, we propose a spiking neural network that unifies two principles — stability through attraction and uncertainty through fluctuation — and reinterpret the HD circuit as an uncertainty-aware integrator rather than a deterministic compass. Specifically, the network uses sampling-based probabilistic inference, where a neural population represents input uncertainty by rapidly fluctuating among likely hypotheses about the world while preserving a stable representation of head direction along an attractor manifold. This formulation suggests why a classical HD “bump” becomes less precise, namely due to rapid fluctuations, reflecting the uncertainty in angular velocity inputs. Our implementation yields experimentally testable predictions: correlated subthreshold voltage fluctuations, multi-timescale nonlinear interaction patterns, and characteristic statistics of bump movement. By combining probabilistic inference with attractor dynamics within one single circuit, our framework suggests how neural populations across species can represent an estimate and its uncertainty through fluctuations while maintaining stability, which could be a general principle for uncertainty-aware computation in noisy biological systems.

## Author summary

Animals rely on navigation for a broad repertoire of behaviors, including foraging, returning home, avoiding threats, finding mates, and moving between familiar locations; in turn, to enable efficient navigation, animals need to maintain an internal representation of navigational variables such as heading. Neural head-direction circuits are often modeled as attractor networks, which maintain a stable “bump” of neural activity representing orientation. However, real sensory inputs are noisy, such that animals must estimate how reliable those inputs are to guide their decision-making. We asked how one neural circuit could both maintain a stable head-direction estimate and represent uncertainty. We developed a spiking neural network model that combines attractor dynamics with sampling-based probabilistic inference. In the model, noisy angular-velocity inputs drive samples that are integrated over time into a head-direction estimate that remains constrained to a circular attractor. This means that fluctuations in the activity bump are not simply noise: they reflect uncertainty in the incoming motion signals. The model also explains how visual landmarks can recalibrate the head-direction representation according to cue reliability, and provides experimentally testable predictions regarding structured voltage correlations, multi-timescale interaction patterns, uncertainty-dependent bump movement, and skewed bump-velocity statistics. Our results suggest that combining attractor dynamics with probabilistic sampling allows neural circuits to represent both an estimate and its uncertainty, providing a general computational mechanism that bridges two traditionally separate frameworks for computation in noisy biological systems.

## Introduction

Animals track their position and orientation as they navigate through uncertain and dynamic environments. To do so, neural populations maintain internal representations of navigational variables—such as head direction (HD) [1,2]—even in the face of noisy or conflicting sensory cues [3–5]. Across species, cue uncertainty modulates population-level HD representations, for example broadening an activity bump in flies and altering network gain and latent structure in mice [6,7]. In general, the brain faces the challenge of integrating uncertain inputs while maintaining coherent internal estimates of external variables and their uncertainties over time [8–11]. Here, we propose a spiking network that addresses this challenge and employ it to shed light on the HD system’s representation dynamics in the face of uncertainties across species.

When faced with uncertain inputs, neural circuits balance two demands: inferring the most likely external signals, potentially while tracking uncertainty [6,12], and maintaining stable, persistent outputs for computation and behavior [13]. Different computational perspectives have been used to describe these functions mechanistically. On the one hand, sampling-based probabilistic inference describes how neural variability can be harnessed to explore the posterior distribution over external signals. In sampling, neural variability is not merely noise, but it reflects a specific computation that generates a sequence of possible stimulus values whose statistics approximate the posterior. This has been used extensively to model the early stages of environmental stimulus processing [14–28], and neural sampling-based mechanisms have accumulated substantial experimental support over the last decade [29–33]. On the other hand, attractor network models explain how recurrent connectivity can stabilize neural activity patterns, supporting persistent representations of navigation variables such as head direction (HD) [13,34–39]. Biologically, there is substantial evidence that attractor-like mechanisms are at play, with prominent examples in HD systems of rodents [40,41] and the central complex of the fruit fly [2,42,43].

How can an HD system represent a navigational variable while maintaining and relaying information about the uncertainty of sensory stimuli? In this work, we will consider the possibility that sampling-based inference and attractor dynamics might both be necessary to model higher-order navigation systems. While stochastic fluctuations have long been incorporated into attractor models of decision making [44], perceptual rivalry [45,46], and multistable memory [47,48], they are usually treated as a perturbation rather than as an explicit mechanism for probabilistic inference. On the other side, prior work has explored stochastic neural dynamics as a mechanism for sampling-based inference [10,18,24,29,30], but without explicitly incorporating structured continuous attractor manifolds as computational primitives. Here, we set out to construct a network in which: a) stochastic fluctuations play a functional role in implementing sampling-based inference; b) inferred quantities are integrated and attracted to a continuous attractor manifold; c) attractor dynamics are derived from a prior which represents the manifold; and d) input uncertainty is represented and broadcast downstream.

**Fig 1:**
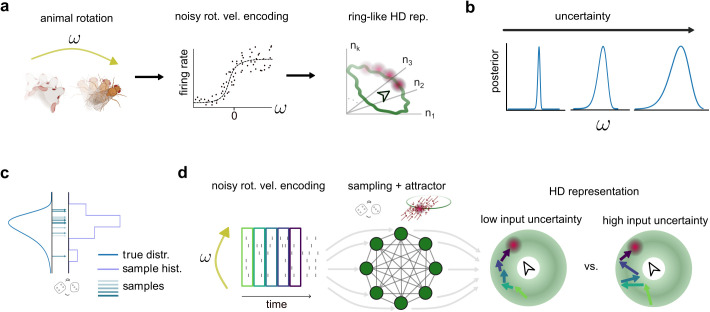
Integrating noisy motion signals through sampling on an attractor. **(a)** Angular velocity ω is encoded noisily by populations of neurons in different species (e.g., GLNO cells in fruit flies [50], brainstem’s dorsal tegmental nucleus (DTN) cells in rats [51]). The HD output activity lies on a circularly arranged set of neurons in the fruit fly’s brain [2], and on a circular manifold in a high−D space in the rat’s brain [41]. **(b)** For increasing noise levels on the encoding of the angular velocity, the posterior distribution will show more uncertainty. **(c)** Estimating true density and statistics through sampling (left line: true distribution; arrows: samples from true distribution; right line: samples histogram). **(d)** Angular velocity inputs are encoded as noisy Poisson spikes, which are in turn fed into a recurrent spiking neural network (SNN) that implements angular velocity sampling, integration, and attraction. The SNN represents the HD by linear readout, and is updated according to angular velocities sampled from the Poisson spikes. The readout will meander around a circular attractor, providing an immediate HD estimate as well as, implicitly, the input uncertainty encoded by the movement of the bump. Components of Fig 1A were created in BioRender. Nardin, M. (https://BioRender.com/x68r143) and (https://BioRender.com/w64d461) are licensed under CC BY 4.0.

Starting from first principles of sampling with spikes [26] and attractor dynamics [49], we derive a spiking neural network model that combines the strengths of both computational paradigms: it uses stochastic voltage fluctuations to fully explore the probability distribution of sensory inputs, and recurrent connectivity to stabilize the integrated estimate on a continuous manifold. Using the HD system as a case study (Fig 1), we show that noise can serve as a computational resource: specifically, angular velocity signals encoded by noisy sensory neurons are continuously sampled, integrated over time, and constrained to evolve near a circular attractor representing head direction. Rather than focusing on the detailed anatomical implementation, here we focus on the high-level principles underlying HD computations: in particular, this framework proposes a novel view of navigation-related computations under uncertainty. Our HD model suggests a novel explanation of recent experimental data on HD dynamics under uncertainty [6,7], and provides testable predictions for subthreshold voltage correlations, connectivity motifs, and variability in bump movement.

## Results

### A spiking network unifies sampling and attractor dynamics

To maintain an internal representation of an animal’s head direction (HD), an HD system must infer the head rotation velocity from noisy inputs [50–52] and update the current HD estimate within circularity constraints [2,13,41] (Fig 1A). We modeled this process with a spiking network that performs probabilistic inference, expanding upon previous work [26,27,49,53,54], with the addition of an attractor-based integrator. The network receives angular velocity inputs from populations of noisy Poisson neurons that encode rotational motion. Noise makes it impossible to exactly recover the true latent variable; from a theoretical perspective, the best one can do is to consider a posterior distribution of possible angular velocities, with noise increasing uncertainty (Fig 1B). In contrast to classical attractor models that set aside or average out noise [34,37,38], our network explicitly treats population activity in short time windows as samples from an underlying probability distribution, a process called probabilistic sampling (Fig 1C, Box 1). Through local recurrent interactions, the network samples an estimate from the posterior distribution over angular velocity, integrates it over time, and attracts the activity pattern towards a circular manifold (Fig 1D). This combination allows the system to represent both the HD estimate and the stimulus encoding uncertainty (Fig 1E), providing a mechanistic bridge between probabilistic and dynamical views of HD computation.

### Mechanisms for integrating velocity samples on an attractor

**Fig 2:**
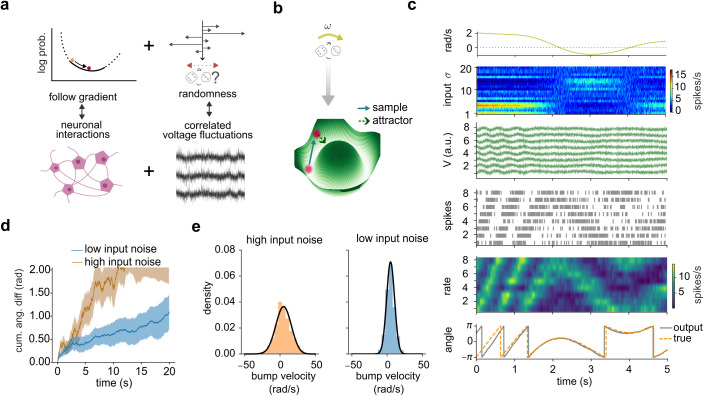
Sampling-based integration of angular velocity on a circular manifold. **(a)** Langevin dynamics allow one to sample from arbitrary probability distributions. This entails following the slope of the log distribution together with a source of stochasticity to promote exploration. In our networks, these terms are implemented via neuronal interactions and correlated voltage stochastic fluctuations, respectively. **(b)** In our implementation, the neural representation of HD consists of two variables *x*,*y*, linked to the angle via transformation x=ρcos(θ) and y=ρsin(θ). Without a mechanism to pull the representation towards $x^{2}+y^{2}=\rho^{2}$, the network readout can go to zero or diverge (see S6 Fig). Hence, we consider a specific prior to attract the network dynamics towards the circle. In this way, the network can explore two dimensions, as suggested in recent experiments [7]. **(c)** Top to bottom: rotational velocity ω; noisy Poisson encoding of rotational velocity $\sigma_{i}\sim P(\sigma_{i}|\omega )$; voltage *V* of a HD network with 8 neurons; HD network output spikes; HD network firing rates obtained by smoothing the spikes with an exponential kernel; network HD output vs. true angle. **(d)** HD representation drift measured as absolute angle difference of network output vs. true orientation during continuous clockwise turning at 5 rad/sec for low vs high input noise, implemented as different numbers of encoding neurons (1000 vs 5000), and averaged across 50 repetitions (solid line: mean; shaded area: 1STD). **(e)** Distribution of instantaneous bump movement for low- vs high-noise inputs for constant clockwise turning at 5 rad/sec. Overlay (black) true posterior distribution averaged over Poisson inputs.

The HD circuit must transform angular velocity, $\omega_{t}$, encoded through Poisson neurons following a distribution $P(\sigma_{t}|\omega_{t})$, into a coherent estimate of orientation, $\theta_{t}$. Our model does this through local interactions by combining a sampling-based inference process with a stabilizing attractor manifold (Fig 1). Specifically, the network samples instantaneous rotational velocity from noisy inputs, and stabilizes the integrated HD estimate by attracting towards a circular manifold, where bump fluctuations encode stimulus uncertainty (Fig 1C). We implement sampling-based inference in spiking neural networks through Langevin sampling (Fig 2A), as proposed in previous work [26,55], which we extended to work with non-Gaussian distributions (Box 1, S1 Text).

**Box 1. Non-Gaussian sampling with Spike Coding Networks**

Non-Gaussian distributions are important in neuroscience to explain various phenomena. For example, songbirds partially reject large auditory feedback perturbations, a phenomenon explained by non-Gaussian, heavy-tailed Bayesian models [56,57]. Similarly, in barn owls, prism-induced sensory conflicts produce adaptation that saturates or depends on gradual training, phenomena inconsistent with Gaussian cue integration [58,59].

In this paper, we use Spike Coding Networks (SCNs) to implement non-Gaussian sampling (S1 Fig). SCNs are a subset of Spiking Neural Networks that have the capability to embed dynamical systems in their network topology and reproduce the prescribed dynamics [53]. This network’s structure is fully derived from first principles and characterized by a combination of fast and slow recurrent connections, with a synaptic connectivity topology that ensures spikes occur only when the network state deviates from the target dynamical state. Probabilistic sampling was previously implemented in such SCNs as a dynamical system through Langevin Dynamics, as shown in [26,27,55].

A linear read-out of the network then $\hat{z}$ represents a sample from a distribution P(𝐳), and over time converges to the correct distribution. However, all previous approaches were limited to purely Gaussian distributions.

Through the use of slow multiplicative synaptic interactions [49], we extended the computational capabilities to non-linear dynamical systems, which in turn allows for sampling from non-Gaussian distributions (S1C Fig). In the Appendix we explore the capability of these networks by sampling from high-dimensional and non-Gaussian distributions, including several multi-modal distributions, and analyze how sampling performance depends on the number of neurons, voltage timescales, and network weights (see S1 Text, S2 and S3 Figs).

The nonlinear extension to sampling from polynomial log-probabilities easily enables sampling-based inference for variables encoded by noisy Poisson neurons (S4 Fig), as is used for the networks in the main text, and also allows for the recovery of higher-order moments of the posterior distribution (S5 fig). The SCN sampling-based inference framework is flexible and can in principle be used for many other approaches besides head direction estimation. For example, it was previously used to study probabilistic systems for decision-making [28]. Our work extends these capabilities to non-Gaussian sampling, and can help deal with multimodal distributions and complex decision-making tasks. Finally, this approach could also be useful for neuromorphic systems that needs to efficiently and quickly estimate probability distributions and perform computations based on them.

We present the high-level steps of our derivations here and point the reader to the methods for a complete derivation. The fundamental computation that our network performs is to continuously update the HD estimate, $\theta_{t}$, using the incoming spikes, $\sigma_{t}$, through approximate Bayesian filtering:


$$\begin{array}{c} P(\theta_{t}|\sigma_{t},\theta_{t-dt})\sim P(\sigma_{t}|\theta_{t},\theta_{t-dt})P(\theta_{t}|\theta_{t-dt}), \end{array}$$ (1)

The first term on the right hand side, $P(\sigma_{t}|\theta_{t},\theta_{t-dt})$, represents the incoming angular velocity information, using $\omega_{t}\propto \theta_{t}-\theta_{t-dt}$, and the second term, $P(\theta_{t}|\theta_{t-dt})$, represents a prior over angles. In our implementation, we consider the angular representation in Cartesian coordinates (x,y)=(ρcos(θ),ρsin(θ)) and a small-angle approximation $\omega_{t}\propto y_{t}x_{t-dt}-x_{t}y_{t-dt}$. Using discrete-time Langevin sampling on $P(x_{t},y_{t}|\sigma_{t},x_{t-dt},y_{t-dt})\propto P(\sigma_{t}|y_{t}x_{t-dt}-x_{t}y_{t-dt})P(x_{t},y_{t}|x_{t-dt},y_{t-dt})$, we derive an update rule of the form:


$$\begin{array}{c} (x_{t},y_{t})=(x_{t-dt},y_{t-dt})+dt\left(\nabla_{x,y}\log P(x_{t},y_{t}|\sigma_{t},x_{t-dt},y_{t-dt})\right)+\sqrt{2dt}\eta_{t} \end{array}$$ (2)

If the gradient of logP is mostly driven by $\nabla \log P(\sigma_{t}|\omega_{t})$, this implementation effectively samples from the posterior over angular velocities given Poisson spikes $\sigma_{t}$. Implementing this method in any neural network requires: 1) dynamics, which follow the gradient of a probability distribution, implemented through neuronal interactions, and 2) stochasticity, to explore the entire probability landscape, implemented through correlated voltage fluctuations [60–62] (Fig 2A). We implemented Eq 2 into a spike coding network, and obtained voltage dynamics of the form:


$$\begin{array}{c} {𝐯}_{t+dt}=(1-\lambda dt){𝐯}_{t}+dt\left(\underset{\text{infer \& integrate}}{\underbrace{I({𝐫}_{t},\sigma_{t})}}+\underset{\text{attractor}}{\underbrace{A({𝐫}_{t})}}+\underset{\text{fast E/I balance}}{\underbrace{\Omega_{f}{𝐨}_{t}}}\right)+\underset{\text{stochastic fluctuations}}{\underbrace{\sqrt{2dt}\;\xi_{t}}} \end{array}$$ (3)

The network consists of ${𝐨}_{t}$ representing spike trains, ${𝐫}_{t}$ representing the filtered spike trains, and a few complementary components derived from first principles (see S1 Text):

- Fast recurrent interactions $\Omega_{f}$ (S1C Fig, top), specific to SCN networks and not related to the inference process, which allow the network to maintain an excitation/inhibition balance [53,54]. This timescale is consistent with AMPA and ${\text{GABA}}_{a}$ receptors, with rapid activation and decay [63].
- Slow feedforward $I({𝐫}_{t},\sigma_{t})$ and recurrent $A({𝐫}_{t})$ interactions (S1C Fig, bottom), mediating linear and nonlinear computations, which implement the gradient of $\log P(x_{t},y_{t}|\sigma_{t},x_{t-dt},y_{t-dt})$. In biology, this timescale is consistent with NMDA and ${\text{GABA}}_{b}$ receptors, with slow rise and longer-lasting activations [64,65]. The nonlinear recurrent interactions $A({𝐫}_{t})$ maintain a localized bump of activity near a circular manifold, providing a stable representation of head direction.
- Finally, correlated voltage fluctuations $\xi_{t}$, which are a high-dimensional projection of $\eta_{t}$ onto the neurons (S1D and S1E Fig), drive the network to explore the posterior distribution over angular velocities. These fluctuations allow the network to continually generate new probabilistic samples, which are then integrated into the evolving head-direction estimate.

The interplay between neuronal interactions and stochastic voltage fluctuations ensures that the network remains anchored to the circular attractor (Figs 2B and S6) while still reflecting sensory uncertainty in its moment-to-moment dynamics. As a baseline, this model reproduces several experimentally observed phenomena. Firstly, the network shows a localized “bump” of activity whose position correlates with the heading direction (Fig 2C). When velocity inputs are reliable, the bump moves smoothly and tracks the true head direction with high precision (Fig 2C). The network maintains a good HD estimate for a period of time but slowly loses the true orientation as the various sources of noise accumulate, reminiscent of biological studies [2,42]. When inputs are noisy or ambiguous, the inferred velocity samples become more variable, leading to faster deviations from true HD (Fig 2D). Furthermore, the average network activity is higher during periods of high angular velocity (S6D Fig), as previously shown [2]. Finally, this HD system can be implemented in very small networks (S7 Fig) [2,66]. Crucially, the movement of the bump implicitly encodes input uncertainty, with short-time bump velocities recovering the angular-velocity posterior (Fig 2E). This sampling-based inference formulation allows downstream areas to compute input uncertainty, or other high-order statistics of the posterior (S5 Fig). In the full HD network, posterior recovery depends on the regime of operation: when the attractor is too weak, the HD representation leaves the circular manifold (S6B and S6E Fig), leading to poor HD estimates; when it is too strong, bump motion is over-constrained and posterior variability is suppressed, leading to poor posterior estimates (S6E Fig).

In conclusion, across a range of parameters, the network maintains a stable circular representation while continuously integrating velocity samples. This results in trajectories that are both stochastic and structured, reminiscent of bump dynamics observed in fly and rodent HD systems [2,40]. Our model, therefore, suggests that stochasticity in HD circuits is not just noise superimposed on a deterministic attractor, but might represent a signature of probabilistic inference operating within an attractor manifold, and serves the purpose of relaying input uncertainty to downstream areas for further computation.

### Visual reset through multimodal integration

Real HD systems have to cope with fluctuating sensory reliability and cues from multiple modalities [4,6,67]. To examine whether our framework deals with these challenges, we extended the model to incorporate additional input pathways and reset mechanisms.

Sampling-based inference is additive in log-probability, so additional inputs simply provide additional constraints on the inferred direction. When cues are consistent, the network rapidly aligns and stabilizes the head-direction estimate; when cues conflict, the network resolves them according to their reliability, producing larger variability in bump motion when the available information is ambiguous, yielding a broader posterior. This behavior does not require explicit tuning but rather emerges from the combination of multiple cues, each weighted according to its reliability, and the combined sampling and attraction process.

### Visual cues realign the attractor through probabilistic biasing

**Fig 3:**
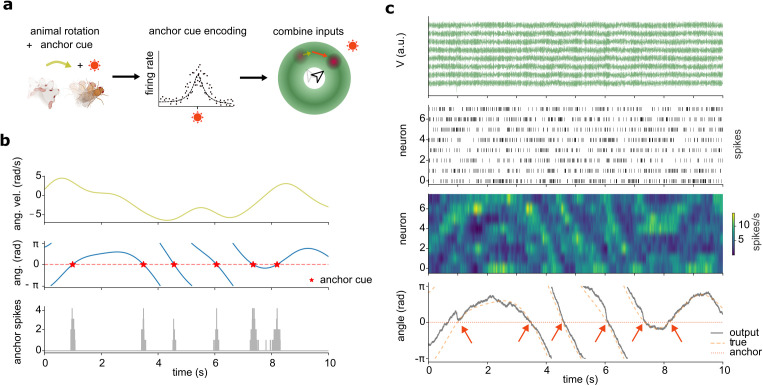
Visual reset realigns the sampling-based attractor through multimodal integration. **(a)** Schematic of the computational setting. The head-direction network integrates noisy angular velocity samples (yellow trace) through sampling-based inference, resulting in stochastic but bounded bump motion on a circular attractor. A transient visual anchor cue (red) provides an additional sensory inputs on orientation; both inputs are integrated according to their reliability. **(b-c)** Representative single trial dynamics during a reset event. During angular rotation, cue is encoded noisily by an external population of visual neurons when θ=0. Before cue onset, uncertainty in sampled angular velocities leads to a gradual drift of the inferred head direction. Activation of the visual anchor cue rapidly biases the network state toward the correct orientation, realigning the activity bump without disrupting ongoing sampling dynamics. After the reset, integration resumes with the same stochastic structure. Components of Fig 3A were created in BioRender. Nardin, M. (https://BioRender.com/x68r143) and (https://BioRender.com/w64d461) are licensed under CC BY 4.0.

In the absence of perfectly reliable inputs, uncertainty in sampled angular velocities leads to a gradual accumulation of bump drift. To mimic the role of stable landmarks observed in both flies and rodents, we introduce a secondary sensory population encoding the angular position of a visual anchor. A transient burst of activity from this population rapidly realigns the activity bump to the correct orientation, sharply reducing accumulated drift (Figs 3A, 3B, and S8). After this reset, the network resumes sampling-based integration of angular velocity as before. Importantly, this realignment mechanism does not overwrite the underlying sampling dynamics. Instead, it pushes the network state toward a more reliable region of the posterior by weighting the incoming visual anchor information based on its reliability.

In flies, landmark alignment involves structured circuit interactions, plasticity, and experience-dependent remapping [2,52,68], whereas in mammals it is often described as landmark-driven realignment of head-direction tuning [1,67]. The dynamics produced by our model are reminiscent of external-cue realignment observed in rats, in which salient landmarks with high reliability rapidly override path-integration signals, realigning head-direction representations in both the anterior thalamic nuclei and postsubiculum [67]. Moreover, recent population-level recordings in mice show that head-direction dynamics during realignment transiently engage an additional latent dimension beyond the angular manifold [7]. Such higher-dimensional structure arises naturally in our framework and becomes particularly apparent during larger reset events, when the external sensory evidence quickly pushes the network towards the anchor orientation, temporarily overcoming the attraction towards the circle (S9 Fig).

These results demonstrate that the sampling-based attractor framework can accommodate biologically inspired computations, such as multimodal integration, and allows rapid realignment to reliable cues.

### Physiological signatures of sampling in head direction systems

A central goal of our combining sampling-based inference with attractor dynamics in a single spiking circuit is to produce specific, experimentally testable predictions. These predictions arise from the implementation of the sampling process, the multiscale connectivity structure of the network, and the attractor stabilization; in turn, they map onto measurable features of real head-direction circuits, demonstrating the ability of this framework to inspire new experiments in biology.

### Prediction 1: Structured subthreshold voltage correlations

**Fig 4:**
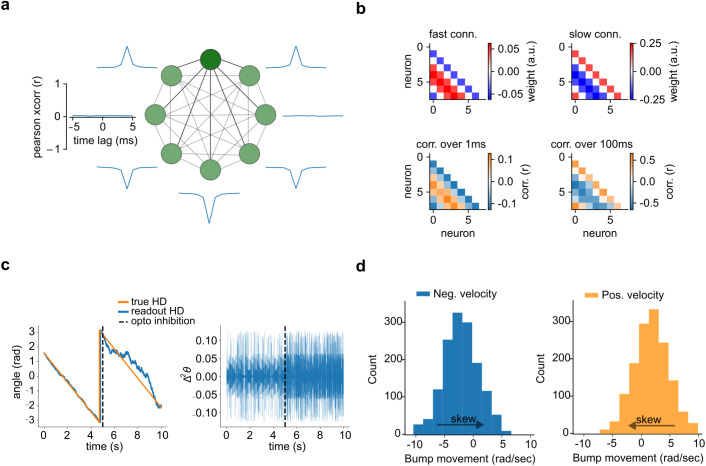
Experimentally testable predictions from networks performing HD computations through sampling. (a) Correlated stochastic fluctuations predict specific patterns of subthreshold voltage correlations among neurons with similar tuning. Top: example voltage trace for dark green neuron. Around the circle: subthreshold voltage cross-correlations between the green neuron and the others in the network. Notice that neurons with opposite tuning exhibit negative subthreshold correlations. **(b)** Top: fast connections $\Omega_{f}$ show nearest neighbor inhibition and long-range excitation, while slow connections $\Omega_{s}$ show a classical nearest neighbor excitation and long-range inhibition. Bottom: short-time vs. long-time correlations in neural activity, measured on spiking activity convolved with a Gaussian kernel of 1ms vs. 100ms. **(c)** Left: decoded vs. true heading in a representative trial. At the dashed line, the outputs of the Poisson neurons encoding the angular velocity are “optogenetically inhibited”, effectuated in the model by reducing the neuronal gains in the population through scaling β→0.2β, after which the estimate drifts more. Right: $\Delta^{2}\theta$ grows after inhibition, showing that reduced input reliability increases bump jitter, leading to less reliable estimates; this is reflected in ensemble statistics, see S12 Fig. **(d)** Distribution of bump velocity for positive and negative angular velocity (±4 rad/sec). The variability in bump movement velocity reflects the uncertainty in the inferred angular velocity.

The sampling process requires neurons to share structured sources of stochastic fluctuation. In our model, voltage noise is correlated across neurons according to their tuning similarity. Neurons with nearby preferred directions exhibit positively correlated subthreshold fluctuations, whereas neurons with opposite tuning show weak or negative correlations (Fig 4A). This pattern reflects the main underlying functional principle of our networks: shared voltage fluctuations enable coordinated exploration of the posterior distribution. This can be tested by computing cross-correlations between the voltages of nearby and distant cells and could be achieved experimentally, for example, in a fly navigating an artificial environment paired with voltage imaging of the central complex neurons [2,69,70], or head-fixed mice navigating in an artificial environment [70]. Moreover, given the absence of sensory stimuli during the larva/pupa stages of fruit fly development, one could study the dimensionality of neural activity, which will be driven by other areas’ inputs and other noisy sources, which taken altogether could be seen as structured voltage noise.

### Prediction 2: Multi-timescale interaction structure

The spiking model we present here, based on the theory of spike coding networks [53], separates computation across two distinct synaptic timescales, similar to previous implementations [26,27,49]. In our case, the fast connections, which operate on a timescale of a few milliseconds and ensure excitation-inhibition balance, show nearest-neighbor inhibition and long-range excitation. On the other hand, slow connections, which operate on the timescale of tens of milliseconds and implement the dynamics, show a ring-like topology with classical nearest neighbor excitation and long-range inhibition, reminiscent of what was used in previous computational studies [39] inspired by true biological connectivity [71] (Fig 4B, top). This is observable in the short-time versus long-time correlations in neural activity (Fig 4B, bottom), an analysis that could be carried out using ultra-fast calcium recording [72] or electrophysiology in the central complex of fruit fly, or with detailed knowledge of the timescales of interactions in a connectome [71]. Nonlinear interactions will act over slow timescales and constrain the activity to the desired manifold. These slower nonlinear interactions might be implemented through widespread peptidergic neuromodulation [73], and could be studied in a peptidergic connectome [74].

### Prediction 3: Uncertainty-dependent bump movement

Because the network integrates samples of angular velocity, the motion of the bump reflects the variability of the sampled posterior rather than solely the mean of the input stream, potentially allowing downstream readers to estimate higher-order statistics of the underlying posterior distribution (S5 Fig). When sensory inputs are reliable, the bump jitters less and moves smoothly; when inputs are noisy or ambiguous, the inferred velocity samples vary more widely, producing larger and more irregular bump displacements (Fig 2E). These fluctuations are not signs of network failure; they reflect increased uncertainty about the inputs. The model links variability in bump speed and position to the statistical structure of incoming sensory spikes. This yields a direct, causal experimental test: optogenetically inhibiting the input population that conveys the angular velocity signal reduces the reliability of the encoded self-motion estimate and should therefore lead to more drift of the HD estimate and increased bump jitter (Figs 4C and S12), which is qualitatively in line with the bump broadening observed experimentally [6]. Our model explains this behavior not as degradation of the attractor, but as a functional expression of probabilistic inference operating within it.

### Prediction 4: Asymmetries and skew in bump velocity distributions

Poisson-like sensory encoding does not, in general, produce symmetric posterior distributions over angular velocity. In particular, for low spike counts and nonlinear tuning curves, the resulting posterior is typically skewed (S1 Text). As a result, the inferred angular velocity exhibits asymmetric fluctuations, which translate into skewed distributions of bump velocity. This effect is especially pronounced at low input reliability, where the posterior is broader and more asymmetric. (Fig 4D). This prediction is tied to the sampling formulation, rather than structural requirements, and it is not expected from noiseless deterministic attractor models; in our network, reducing or removing the correlated voltage fluctuations reduced bump movement and leads to biased angular velocity estimates (S11 Fig). The prediction of skewed posteriors also differs from models in which fluctuations are treated as a perturbation of deterministic attractor dynamics, in that noise is typically symmetric and leads to less skew (S10 Fig). Assessing the skewness of fast bump movement might be feasible using fast imaging or electrophysiological measurements.

## Discussion

Neural circuits maintain stable internal representations while preserving information about the uncertainty of inputs. We propose how these two requirements can be reconciled within a single spiking network by embedding sampling-based inference inside a continuous attractor. Concretely, we implemented inference as Langevin sampling of angular velocity from noisy spiking inputs, combined with a soft circular prior that stabilizes the integrated velocity vector inputs into a head direction (HD) estimate. This unifies two computations that are often treated separately: attractor dynamics that support persistent population codes, and probabilistic computation in which variability reflects uncertainty rather than being simple noise. While we do not claim that our model is a complete, circuit-level reproduction of any specific HD system, our goal is to identify requirements for sampling-based inference within attractor-based spiking circuits to inspire future experiments. As such, this model provides experimentally testable predictions related to the connectivity and neural activity correlations over different timescales, subthreshold inputs, and other bump kinematics measurements.

### Head direction as an uncertainty-aware integrator

Classical ring attractor models of head direction typically treat angular velocity as a control signal that deterministically moves the bump, with noise introduced as a perturbation [35,37,38]. In contrast, we treat velocity inputs as noisy observations and cast decoding as an inference problem. The resulting dynamics can be understood as integrating samples from a posterior distribution over angular velocity while maintaining a stable circular representation of head direction. Under the assumption that neural dynamics are faster than behavioral dynamics, this reduces to integrating the posterior mean; at the same time, the movement of the bump preserves information about the uncertainty of the inputs, with the sampling formulation allowing for the computation of higher-order statistics of the posterior. Because the network samples from a posterior over angular velocity, increased variability in the bump trajectory corresponds to increased uncertainty in the self-motion signal being integrated. This suggests a potential functional role for these fluctuations: downstream circuits could use the temporal coherence of the HD representation as an implicit estimate of reliability, without requiring an explicit decoding of variance. More concretely, such fluctuations could support reliability-dependent cue integration. Increased bump variability would indicate that path integration is unreliable, favoring stronger weighting of external cues during reorientation, while stable dynamics would indicate high-confidence self-motion integration. In this way, the temporal statistics of HD activity could serve as an implicit reliability signal for downstream navigation circuits.

This perspective differs from approaches that explicitly track a variance-like quantity. For example, a Bayesian ring-attractor formulation based on a circular Kalman filter predicts that uncertainty/variance is reflected in bump amplitude and that direction selectivity should progressively decrease in the absence of visual inputs [75]. In contrast, in our model, increased uncertainty primarily manifests as an increased bump movement driven by the sampling process, while the instantaneous (~1ms) bump profile remains fairly constant. In flies, the HD bump can remain stable in darkness without losing direction selectivity [2,39,52], yet recent work reported bump width broadening under cue conflict and uncertainty [6]. These observations can be reconciled within our framework: increased uncertainty produces larger excursions of the bump trajectory, which can broaden the time-averaged (10~100 ms) population activity even when the instantaneous bump shape is stable. Consistent with experimental reports, our simulations also reproduce hallmark features of head-direction dynamics, including increased activity during fast rotations and progressive loss of absolute orientation in darkness [2,52], and they achieve accurate integration with relatively small networks, consistent with compact insect circuits and recent theoretical results [39,76]. Finally, recent population-level recordings in mice suggest that HD networks during realignment explore a second dimension, beyond the circular manifold [7], which arises naturally in our model during cue-driven correction, due to the fact that we implemented velocity vector integration with a soft 2D circular prior, and rapid realignment forces the network to deviate from the vicinity of the circle briefly.

### Feasibility of implementation and predictions

The model is theoretically derived from first principles, providing clear dependencies between aspects of the model and consequent phenomena and predictions. Implementing Langevin sampling requires two ingredients: (i) a drift term proportional to gradients of a log-probability and (ii) structured fluctuations that drive exploration of the distribution. In our networks, the drift term is generally nonlinear and can be implemented using multiplicative interactions [49]. Such nonlinearities can arise from dendritic mechanisms and synaptic integration, and they can also be approximated using additional populations or learned nonlinear function approximators [77–80]. The stochastic component requires shared fluctuations, such as correlated subthreshold activity and shared variability, which are widely observed and provide a plausible biological substrate [60,61,81]. Our contribution is not simply to add stochasticity to an attractor network, as noise has been incorporated into attractor models in several contexts [44–48]. Instead, we assign a functional role to neural variability within attractors through a principled sampling-based inference method. The computation we implemented is Langevin sampling with a soft circular constraint, and we used the theory of spike coding networks (SCNs), which offer an analytical solution to embedding low-dimensional dynamics into a spiking neural network [26,27,53,54]. Other implementations can, in principle, approximate the same drift and diffusion terms using alternative spiking frameworks or learning-based approaches, including the Neural Engineering Framework or recurrent networks with learned nonlinearities [79,80,82,83]. This distinction matters for interpretation of the physiological predictions: some follow from the sampling process, such as subthreshold voltage correlations, uncertainty-dependent bump movement statistics, and skewed statistics of inferred velocities, whereas others depend on the particular SCN implementation, such as distinct fast and slow interactions. Overall, the model’s interpretability makes the dependencies between model components and their associated phenomenology explicit, allowing causes and effects to be cleanly separated.

### Relation to previous work

Several previous works have described functional roles for spiking variability in recurrent dynamics. For example, variability can stabilize bistable states in balanced spiking networks with nonlinear synaptic transmission [84], and can support nonlinear dynamics in fluctuation-driven networks of winner-take-all units [85]. Our framework is complementary: rather than treating variability as a mechanism for stabilizing or generating nonlinear dynamics, we use correlated voltage fluctuations to implement posterior sampling.

Our framework connects and extends two active lines of research. First, multiple spiking models have implemented ring-attractor dynamics for head direction in both insects and mammals, often aiming to capture circuit anatomy and deterministic bump updates [86,87]. Second, sampling-based probabilistic computation has been implemented in spiking networks using MCMC-like dynamics or Langevin samplers [20–22,26,27]. Our contribution is to show how sampling-based inference can be embedded within a continuous attractor so that the circuit simultaneously maintains a stable representation and encodes uncertainty through sampling dynamics.

### Outlook

We have explored how sampling-based computations and attractor dynamics can be implemented within spiking neural networks, specifically in the case of the HD system. This work supports the proposition of sampling-based methods as a viable and powerful framework for neural computation in non-sensory brain areas, and in turn offers testable hypotheses for experimental and computational neuroscience. Future work can extend these ideas to higher-dimensional manifolds (e.g., toroidal representations for grid-cell dynamics) and other task domains (e.g., decision making), incorporate plasticity to learn or approximate nonlinear interactions, and compare predicted subthreshold and population-level statistics to experimental data to shed light on probabilistic computations in the brain.

## Materials and methods

Here, we highlight the main formalism for deriving head-direction representation by inferring angular velocity and integrating it onto an attractor, and the implementation thereof into an SCN; for a didactic build-up of the methodology, see the Appendix.

### HD representation through integration of sampling-based inferred angular velocity

We want our network to represent the current head direction (HD), $\theta_{t}$, which is updated by combining the previous HD estimate, $\theta_{t-dt}$ and the noisily encoded angular velocity signal, $\sigma_{t}$, provided by the external neural population. We formalize this requirement in a probabilistic sense, considering


$$\begin{array}{c} \theta_{t}\sim P(\theta_{t}|\sigma_{t},\theta_{t-dt}). \end{array}$$ (4)

This probability can be decomposed using Bayes’ rule:


$$\begin{array}{c} P(\theta_{t}|\sigma_{t},\theta_{t-dt})\propto P(\sigma_{t}|\theta_{t},\theta_{t-dt})P(\theta_{t}|\theta_{t-dt}), \end{array}$$ (5)

We consider the case where the external population input, $\sigma_{t}$, depends only on the change in angle, $\theta_{t}-\theta_{t-dt}\propto \omega_{t}$, and assume that


$$\begin{array}{c} P(\sigma_{t}|\theta_{t},\theta_{t-dt})=P(\sigma_{t}|\theta_{t}-\theta_{t-dt})\approx P(\sigma_{t}|\omega_{t}), \end{array}$$ (6)

such that Eq 5 becomes


$$\begin{array}{c} \underset{\text{HD angle posterior}}{\underbrace{P(\theta_{t}|\sigma_{t},\theta_{t-dt})}}\propto \underset{\text{ang. vel. input}}{\underbrace{P(\sigma_{t}|\omega_{t})}}\underset{\text{prior}}{\underbrace{P(\theta_{t}|\theta_{t-dt})}}. \end{array}$$ (7)

The term $P(\sigma_{t}|\omega_{t})$ will inform the HD system about the angular velocity $\omega_{t}$ through Poisson spikes $\sigma_{t}$, while the term $P(\theta_{t}|\theta_{t-dt})$ will implement a prior, which can take different forms, as described below.

Consider first Langevin sampling in $\theta_{t}$: assume that $P(\sigma_{t}|\omega_{t})\sim \prod_{i=1}^{N}Poiss(\exp(\beta_{i}\omega_{t}))$, where we use β to denote the vector containing all weights $\beta_{i}$ associated with each Poisson input neurons, and a flat prior $P(\theta_{t}|\theta_{t-dt})\propto c$:


$$\begin{array}{ccc} d\theta_{t} & = & \nabla_{\theta }\log P(\theta_{t}|\sigma_{t},\theta_{t-dt})dt+\sqrt{2dt}\;\eta_{t} \end{array}$$ (8)

$$\begin{array}{ccc} & = & dt(-\beta \exp(\beta (\theta_{t}-\theta_{t-dt}))+\beta \sigma_{t})+\sqrt{2dt}\;\eta_{t} \end{array}$$ (9)

$$\begin{array}{cc} & \approx dt(-\beta (1+\beta (\theta_{t}-\theta_{t-dt})))+\beta \sigma_{t})+\sqrt{2dt}\;\eta_{t}, \end{array}$$ (10)

where $\eta_{t}\sim N(0,1)$. Denote $\alpha =1+\beta^{\;𝖳}\beta dt$, then, assuming $d\theta_{t}\approx \theta_{t}-\theta_{t-dt}$ and rearranging, we obtain


$$\begin{array}{c} \theta_{t}=\theta_{t-dt}+\frac{\beta }{\alpha }(\sigma_{t}-1)dt+\frac{\sqrt{2dt}}{\alpha }\eta_{t}. \end{array}$$ (11)

This equation could be implemented in a SCN, see S1 Text, but would suffer from angular wraparound issues at the −π→π boundary. To avoid this problem, we will adopt a 2−D formalism, as explained below.

### Softening of circular constraints and shift to Cartesian coordinates

To avoid angular wraparound issues, and allow for the exploration of a second dimension as suggested by [7], we consider the angular representation in *x*,*y* coordinates:


$$\begin{array}{c} P(\theta_{t}|\sigma_{t},\theta_{t-dt})=P(x_{t},y_{t}|\sigma_{t},x_{t-dt},y_{t-dt}). \end{array}$$ (12)

This will require us to soften the strict circular constraint that is intrinsic when using only θ. We still assume that knowing *x*,*y* will not inform us about the angular velocity, i.e., P(ω|x,y)=P(ω).

We assume that the angular velocity is well approximated in Cartesian coordinates by the small angle approximation


$$\begin{array}{cc} \omega_{t}\approx \Delta \theta_{t} & \approx \sin(\Delta \theta_{t})\\ & =\sin(\theta_{t}-\theta_{t-dt})\\ & =\sin(\theta_{t})\cos(\theta_{t-dt})-\cos(\theta_{t})\sin(\theta_{t-dt})\\ & =y_{t}x_{t-dt}-x_{t}y_{t-dt}. \end{array}$$ (13)

We consider a population of Poisson neurons responding to the angular velocity through a kernel $\phi_{t}=\phi (\omega_{t})\approx \phi (y_{t}x_{t-dt}-x_{t}y_{t-dt})$ and a soft ring prior of radius ρ with form $P(x_{t},y_{t}|x_{t-dt},y_{t-dt})\propto \exp\left(-\gamma {\left(x_{t}^{2}+y_{t}^{2}-\rho^{2}\right)}^{2}\right)$ (Fig 4B and 4C). These choices allow us to rewrite Eq 6 as:


$$\begin{array}{cc} P(x_{t},y_{t}|\sigma_{t},x_{t-dt},y_{t-dt}) & \propto P(\sigma_{t}|y_{t}x_{t-dt}-x_{t}y_{t-dt})P(x_{t},y_{t}|x_{t-dt},y_{t-dt})\\ & \propto \exp[-\phi_{t}+\sigma_{t}\log(\phi_{t})-\log(\sigma_{t}!)\\ & \;-\gamma {\left(x_{t}^{2}+y_{t}^{2}-\rho^{2}\right)}^{2}]. \end{array}$$ (14)

Taking the log, we get


$$\begin{array}{cc} \log P(x_{t},y_{t}|\sigma_{t},x_{t-dt},y_{t-dt}) & =-\phi_{t}+\sigma_{t}\log(\phi_{t})-\log(\sigma_{t}!)\\ & \;-\gamma {\left(x_{t}^{2}+y_{t}^{2}-\rho^{2}\right)}^{2}. \end{array}$$ (15)

The exact form of the Langevin sampler will depend on the choice of the firing rate nonlinearity of the Poisson neurons. For example, using an exponential kernel


$$\begin{array}{c} \phi (y_{t}x_{t-dt}-x_{t}y_{t-dt})=\exp(\beta (y_{t}x_{t-dt}-x_{t}y_{t-dt})), \end{array}$$ (16)

where β is the neuronal gain, and taking the gradient $\nabla_{x,y}\log P$, leads to


$$\begin{array}{cc} \nabla_{x_{t},y_{t}}\log & P(x_{t},y_{t}|\sigma_{t},x_{t-dt},y_{t-dt})\\ & =[\begin{array}{c} -y_{t-dt}\\ x_{t-dt} \end{array}]\beta (\sigma_{t}-\exp(\beta (y_{t}x_{t-dt}-x_{t}y_{t-dt}))-4\gamma [\begin{array}{c} x_{t}\\ y_{t} \end{array}]\left(x_{t}^{2}+y_{t}^{2}-\rho^{2}\right)\\ & \approx [\begin{array}{c} -y_{t-dt}\\ x_{t-dt} \end{array}]\beta (\sigma_{t}-1-\beta (y_{t}x_{t-dt}-x_{t}y_{t-dt}))-4\gamma [\begin{array}{c} x_{t}\\ y_{t} \end{array}]\left(x_{t}^{2}+y_{t}^{2}-\rho^{2}\right). \end{array}$$ (17)

Considering the Langevin sampler in discrete time, and assuming that $x_{t}^{2}+y_{t}^{2}\approx x_{t-dt}^{2}+y_{t-dt}^{2}$, one gets


$$\begin{array}{cc} \left[\begin{array}{c} x_{t}\\ y_{t} \end{array}]=[\begin{array}{c} x_{t-dt}\\ y_{t-dt} \end{array}\right] & +dt\beta [\begin{array}{c} -y_{t-dt}\\ x_{t-dt} \end{array}](\sigma_{t}-1-\beta y_{t}x_{t-dt}+\beta x_{t}y_{t-dt})\\ & -4\gamma dt[\begin{array}{c} x_{t-dt}\\ y_{t-dt} \end{array}](x_{t-dt}^{2}+y_{t-dt}^{2}-\rho^{2})+\sqrt{2dt}\eta_{t}, \end{array}$$ (18)

where η~𝒩(0,𝐈). Assuming small angles, we may write


$$\begin{array}{c} {}[\begin{array}{c} -y_{t-dt}\\ x_{t-dt} \end{array}](y_{t}x_{t-dt}-x_{t}y_{t-dt})\approx [\begin{array}{c} x_{t}-x_{t-dt}\\ y_{t}-y_{t-dt} \end{array}], \end{array}$$ (19)

and by moving the terms in $\cdot_{t}$ to the left-hand side, simplifying, and again $\alpha =1+dt\beta^{\;𝖳}\beta$, we obtain the equation


$$\begin{array}{cc} \left[\begin{array}{c} x_{t}\\ y_{t} \end{array}]=[\begin{array}{c} x_{t-dt}\\ y_{t-dt} \end{array}\right] & +dt\frac{\beta }{\alpha }[\begin{array}{c} -y_{t-dt}\\ x_{t-dt} \end{array}](\sigma_{t}-1)\\ & -\frac{4}{\alpha }\gamma dt[\begin{array}{c} x_{t-dt}\\ y_{t-dt} \end{array}](x_{t-dt}^{2}+y_{t-dt}^{2}-\rho^{2})+\frac{\sqrt{2dt}}{\alpha }\eta_{t}. \end{array}$$ (20)

This equation resembles a noisy 2−D angular velocity integrator informed by $\sigma_{t}$, with a nonlinear part that implements a soft circular constraint, whose strength can be regulated by an extra parameter γ, and takes the form of a limit cycle of radius ρ [88]. This general procedure could be extended to more complicated examples, which we will explore below after considering the implementation of the equation in an SCN model.

### HD representation through sampling-based inference and attractor dynamics in a SCN

The SCN model has the general form


$$\begin{array}{c} \dot{𝐯}=-\lambda 𝐯+{𝐃}^{\;𝖳}\left(F(𝐳)+\lambda 𝐳\right)-{𝐃}^{\;𝖳}𝐃𝐨, \end{array}$$ (21)

where *F* specifies the target dynamics, which are tracked by the network and can be linearly read out from the spike rates through $\hat{z}=𝐃𝐫\approx 𝐳$. This formalism has been treated for linear systems, i.e., F(𝐳) is linear in 𝐳 [53], and has been adapted for sampling from Gaussian distributions [26]. We further extended this framework to perform sampling-based inference of non-Gaussian distributions through the use of multiplicative interactions in SCNs, which allow nonlinear dynamics such as $F(𝐳)\sim \sum_{k}{𝐳}^{k}$ [49]. By using multiplicative interactions in Eq 21, we can directly implement Eq 20 into an SCN, leading to the governing voltage equation:


$$\begin{array}{c} {𝐯}_{t+1}=(1-\lambda dt){𝐯}_{t}+dt\left(\underset{\text{infer \& integrate}}{\underbrace{I({𝐫}_{t},\sigma_{t})}}+\underset{\text{attractor}}{\underbrace{A({𝐫}_{t})}}+\Omega_{s}{𝐫}_{t}+\Omega_{f}{𝐨}_{t}\right)+\frac{\sqrt{2dt}}{\alpha }\;\xi_{t} \end{array}$$ (22)

where


$$\begin{array}{c} I({𝐫}_{t},\sigma_{t})=-\frac{\beta^{\;𝖳}(\sigma_{t}-1)}{\alpha }{𝐃}^{\;𝖳}[\begin{array}{c} {𝐃}^{\left(1\right)}{𝐫}_{t}\\ -{𝐃}^{\left(0\right)}{𝐫}_{t} \end{array}] \end{array}$$ (23)

with ${𝐃}^{\left(i\right)}$ denoting the i−th row of ${𝐃}^{\;𝖳}$, and


$$\begin{array}{c} A({𝐫}_{t})=\frac{-4\gamma }{\alpha }{𝐃}^{\;𝖳}𝐃{𝐫}_{t}((𝐃{𝐫}_{t}{)}^{2}-\rho^{2}). \end{array}$$ (24)

For completeness: $\Omega_{s}={𝐃}^{\;𝖳}𝐃$ is slow recurrent connectivity contributing to signal representation; $\Omega_{f}=-{𝐃}^{\;𝖳}𝐃$ is fast recurrent connectivity for maintaining excitation/inhibition balance; and $\xi ={𝐃}^{\;𝖳}\eta$, with η~𝒩(0,𝐈), is projected noise for correlated voltage fluctuations. Note that in the main text we simplify the equation for readability, omitting the slow linear term $\Omega_{s}{𝐫}_{t}$ and the damping factor α.

### Multimodal integration and possible reset mechanisms

The HD system receives both proprioceptive and allocentric cues, and it can reset towards a known landmark, which necessitates the integration of visual and angular velocity cues [2,35]. Consider an external input to the network, $\sigma^{\left(reset\right)}$, that interacts with the other terms as in


$$\begin{array}{c} P(\theta_{t}|\sigma_{t},\sigma^{\left(reset\right)},\theta_{t-dt})\propto P(\sigma_{t}|\theta_{t},\theta_{t-dt})P(\theta_{t}|\theta_{t-dt},\sigma^{\left(reset\right)}), \end{array}$$ (25)

and the prior $P(\theta_{t}|\theta_{t-dt},\sigma^{\left(reset\right)})$ is such that $P(\theta_{t}|\theta_{t-dt},\sigma^{\left(reset\right)})=P(\theta_{t}|\theta_{t-dt})$ if $\sigma^{\left(reset\right)}=0$, and $P(\theta_{t}|\theta_{t-dt},\sigma^{\left(reset\right)})\sim 𝒩\left(0,f(\sigma^{\left(reset\right)})\right)$ otherwise.

A slightly more realistic example could entail an external input that encodes the direction of a known landmark, again at θ=0, noisily, such as $P(\sigma^{\left(reset\right)}|\theta )\propto \text{Poiss}(c\exp(-\theta^{2}))$ and $P(\theta_{t}|\theta_{t-dt},\sigma^{\left(reset\right)})=P(\sigma^{\left(reset\right)}|\theta_{t})P(\theta_{t}|\theta_{t-dt})$. In that case,


$$\begin{array}{c} \underset{\text{angle posterior}}{\underbrace{P(\theta_{t}|\sigma_{t},\sigma^{\left(reset\right)},\theta_{t-dt})}}\propto \underset{\text{ang. vel. input}}{\underbrace{P(\sigma_{t}|\theta_{t}-\theta_{t-dt})}}\underset{\text{reset}}{\underbrace{P(\sigma^{\left(reset\right)}|\theta_{t})}}\underset{\text{prior}}{\underbrace{P(\theta_{t}|\theta_{t-dt})}}. \end{array}$$ (26)

This can be implemented in 1−D as


$$\begin{array}{cc} \theta_{t}-\theta_{t-dt} & =\nabla_{\theta }\log P(\theta_{t}|\sigma_{t},\sigma^{\left(reset\right)},\theta_{t-dt})dt+\sqrt{2dt}\eta_{t} \end{array}$$ (27)

$$\begin{array}{cc} & =dt(-\beta \exp(\beta (\theta_{t}-\theta_{t-dt}))+\beta \sigma_{t}+2\theta_{t-dt}\left(c\exp(-\theta_{t-dt}^{2})-\sigma^{\left(reset\right)}\right)+\sqrt{2dt}\eta_{t} \end{array}$$ (28)

and in 2−D, assuming $P(\sigma^{\left(reset\right)}|x,y)\propto \text{Poiss}(c\exp(-(x-\rho {)}^{2}-y^{2}))$, as


$$\begin{array}{cc} \left[\begin{array}{c} x_{t}\\ y_{t} \end{array}]=[\begin{array}{c} x_{t-dt}\\ y_{t-dt} \end{array}\right] & +dt\beta [\begin{array}{c} -y_{t-dt}\\ x_{t-dt} \end{array}](\sigma_{t}-1-\beta x_{t}y_{t-dt}+\beta y_{t}x_{t-dt}) \end{array}$$ (29)

$$\begin{array}{cc} & +2dt[\begin{array}{c} x_{t-dt}-\rho \\ y_{t-dt} \end{array}](\exp(-(x-\rho {)}^{2}-y^{2})-\sigma^{\left(reset\right)})\\ & -4dt[\begin{array}{c} x_{t-dt}\\ y_{t-dt} \end{array}](x_{t-1}^{2}+y_{t-1}^{2}-\rho^{2})+\sqrt{2dt}\eta_{t}, \end{array}$$ (30)

## Supporting information

S1 Text Extended methods. The extended methods contain the underlying derivations and additional details for the mathematical formulation presented in the Methods, as well as a convergence analysis for sampling distributions with varying parameters. (PDF)

S1 Fig Spiking neural networks can sample from arbitrary distributions through a combination of nonlinear synaptic interactions and correlated voltage fluctuations. (PDF)

S2 FigSampling from high −D non-Gaussian distributions in mSCNs.(PDF)

S3 Fig Sampling behaviour and performance for various distributions. (PDF)

S4 Fig Sampling-based inference of Poisson encoded angular velocity signals. (PDF)

S5 Fig Sampling-based inference allows the computation of various statistics online on time-varying stimuli posterior distributions. (PDF)

S6 FigConstraining 2−D angular representations.(PDF)

S7 Fig HD implementations in small vs large networks. (PDF)

S8 Fig Performance of a visual reset mechanism. (PDF)

S9 Fig Excursions from circular manifold. (PDF)

S10 Fig Comparison of independent voltage noise vs correlated voltage fluctuations. (PDF)

S11 Fig Removing correlated voltage fluctuation biases network posterior mean estimate. (PDF)

S12 Fig Ensemble statistics for optogenetic inhibition experiment. (PDF)
