# Supplementary material for: Combining sampling and attractor dynamics in spiking models of head direction systems
Source: PLoS Comput Biol. 2026 Jul 30;22(7):e1014577. doi: 10.1371/journal.pcbi.1014577 (PMC13450861; doi:10.1371/journal.pcbi.1014577)
Supplement: Supplementary file 1 [file pcbi.1014577.s001.pdf]

Extended methods — Combining sampling and attractor dynamics in spiking models of head direction systems

Vojko Pjanovic[1,2], Jacob A. Zavatone-Veth[3], Paul Masset[4,5], Sander W. Keemink[2,&], Michele Nardin[1,*,&]

1 Janelia Research Campus, Howard Hughes Medical Institute, Ashburn, Virginia, USA
2 Department of Machine Learning and Neural Computing, Donders Institute for Brain, Cognition and Behaviour, Radboud University, Nijmegen, Netherlands
3 Society of Fellows and Center for Brain Science, Harvard University, Cambridge, Massachusetts, USA
4 Department of Psychology, McGill University, Montréal, Québec, Canada
5 Mila - Quebec Artificial Intelligence Institute, Montréal, Québec, Canada

& These authors contributed equally to this work.
* Correspondence to nardinm@janelia.hhmi.org

Notational conventions

We introduce the symbols used throughout this work in Table 1, and specify dimensionalities or values used.

Table 1. Notational conventions

Symbol	Meaning	Type / Dimension
N	Number of neurons	Variable
V	Voltage	N
T	Threshold	N
o	Spikes	N
r	Filtered spike train	N
M	Dimension of dynamical variable	Variable
z	Dynamical variable	M
c	External input	M
D	Decoding matrix	(N, M)
$\hat{\cdot}$	Estimate	Notation
λ	Voltage leak	Variable
Ω	Connectivity matrix	(N, N)
$\otimes$	Kronecker product	Operator
E	Cost function	Scalar
ν	Linear cost control	Variable
μ	Quadratic cost control	Variable
A	Linear dynamical system	(M, M)
$F(\cdot)$	Polynomial dynamical system	Function
dt	Simulation time step	Variable
P	Probability distribution	Function
U	Energy function	Function
∇	Gradient	Operator
η	Brownian noise (Langevin)	M
N_p	Number of Poisson neurons	Variable
σ	Sensory neuron spikes	N_p
ϕ	Poisson firing kernel	Function
β	Rate parameter	(N_p, M)
τ_s	Sampling timescale	Variable
θ	Angle	Scalar
ω	Angular velocity	Scalar
ρ	Ring radius	Scalar

Spike Coding Networks

We start by introducing the model by [1], upon which we will expand in the coming sections.

The goal is deriving the equations of a network that follows the temporal evolution of a dynamical signal $\mathbf{z} \in \mathbb{R}^M$,

$$\dot{\mathbf{z}} = \mathbf{A}\mathbf{z} + \mathbf{c}(t), \tag{1}$$

where $\mathbf{A} \in \mathbb{R}^{M \times M}$ is the state-transition matrix and $\mathbf{c} \in \mathbb{R}^M$ is the external input. The network readout, $\hat{\mathbf{z}} \in \mathbb{R}^M$, is an estimate of the signal $\mathbf{z}$,

$$\hat{\mathbf{z}}(t) \approx \mathbf{z}(t), \tag{2}$$

and is updated dynamically using the spiking of the N neurons, $\mathbf{o}(t)$:

$$\dot{\hat{\mathbf{z}}}(t) = -\lambda \hat{\mathbf{z}}(t) + \mathbf{D}\mathbf{o}(t), \tag{3}$$

where λ is the leak term, and $\mathbf{D}$ is an inherent and fixed decoding weight matrix of size $M \times N$. For simplicity, we will consider the vector $\mathbf{r}$ of filtered spike trains

$$\dot{\mathbf{r}}(t) = -\lambda \mathbf{r} + \lambda \mathbf{o}(t), \tag{4}$$

such that the stimulus estimate

$$\hat{\mathbf{z}}(t) = \frac{1}{\lambda} \mathbf{D} \mathbf{r}(t). \tag{5}$$

To minimize the difference between the real signal $\mathbf{z}$ and the readout $\hat{\mathbf{z}}$, the cost function $E(t)$ is minimized

$$E(t) = \int_0^t du \left[\|\mathbf{z}(u) - \hat{\mathbf{z}}(u)\|_2^2 + \nu \|\mathbf{r}(u)\|_1 + \mu \|\mathbf{r}(u)\|_2^2 \right], \tag{6}$$

where the first term minimizes the difference between signal and estimate, the second term forces to minimize the amount of spikes, and the third term distributes spikes across neurons, leading to efficient and balanced spiking, respectively.

Consequently, the network dynamics will perform greedy minimization of $E(t)$, such that they only spike if it leads to a decrease in $E(t)$. The spiking rule for neurons will be

$$v_i(t) > T_i, \tag{7}$$

where

$$v_i(t) = \mathbf{D}_i^\mathsf{T} (\mathbf{z}(t) - \hat{\mathbf{z}}(t)) - \mu \lambda r_i(t), \tag{8}$$

and

$$T_i(t) = \frac{1}{2} \nu \lambda + \mu \lambda^2 + \|\mathbf{D}_i\|^2. \tag{9}$$

With $\nu, \mu = 0$,

$$V(t) = \mathbf{D}^\mathsf{T} (\hat{\mathbf{z}}(t) - \mathbf{z}(t)), \tag{10}$$

and

$$\mathbf{T} = \frac{1}{2} \|\mathbf{D}\|_2^2, \tag{11}$$

Taking the time-derivative of $\mathbf{v}$, rearranging, and using the approximation $\mathbf{z} \approx \hat{\mathbf{z}}$, we obtain [1–3]

$$\dot{\mathbf{v}} = -\lambda \mathbf{v} + \mathbf{D}^\mathsf{T} (\mathbf{A} \hat{\mathbf{z}} + \mathbf{c}(t) + \lambda \hat{\mathbf{z}}) - \mathbf{D}^\mathsf{T} \mathbf{D} \mathbf{o}. \tag{12}$$

Multiplicative interactions in SCNs

If the dynamics of $\mathbf{z}$ are described by a linear system,

$$\dot{\mathbf{z}} = \mathbf{A} \mathbf{z}, \tag{13}$$

we can re-write eq. 12 as

$$\dot{\mathbf{v}} = -\lambda \mathbf{v} + \Omega_f \mathbf{o} + \Omega_s \mathbf{r}, \tag{14}$$

where

$$\begin{aligned} \Omega_f &= -\mathbf{D}^\mathsf{T} \mathbf{D} \\ \Omega_s &= \mathbf{D}^\mathsf{T} (\mathbf{A} + \lambda \mathbf{I}) \mathbf{D} \end{aligned} \tag{15}$$

are the fast and slow recurrent connectivity matrices, respectively. In the case of non-linear systems of the form $\dot{\mathbf{z}} = F(\mathbf{z})$, the resulting model has formal network dynamics given by

$$\dot{\mathbf{v}} = -\lambda\mathbf{v} + \mathbf{D}^\mathsf{T}(F(\hat{\mathbf{z}}) + \lambda\hat{\mathbf{z}}) - \mathbf{D}^\mathsf{T}\mathbf{D}\mathbf{o}, \tag{16}$$

where $F(\cdot)$ is a non-linear function. One approach is to approximate the function F by using a polynomial expansion up to order g:

$$F(\mathbf{z}) \approx \sum_{d=0}^{g} \mathbf{A}_d \mathbf{z}^{\otimes d}, \tag{17}$$

where $\otimes$ denotes the Kronecker product (see below). This derivation quantifies the minimum set of multiplicative interactions necessary for implementing a polynomial dynamical system in a SCN, which we call a multiplicative SCN (mSCN) [4]:

$$\dot{\mathbf{v}} = -\lambda\mathbf{v} + \Omega_f\mathbf{o} + \sum_{d=0}^{g} \Omega_s^{md}\mathbf{r}^{\otimes d}, \tag{18}$$

where

$$\begin{aligned} \Omega_f &= -\mathbf{D}^\mathsf{T}\mathbf{D}, \\ \Omega_s^{m1} &= \mathbf{D}^\mathsf{T}(\mathbf{A}_1 + \lambda\mathbf{I})\mathbf{D}, \\ \Omega_s^{md} &= \mathbf{D}^\mathsf{T}\mathbf{A}_d\mathbf{D}^{\otimes d}. \end{aligned} \tag{19}$$

All these interactions could be carried out in the dendritic tree of a neuron [5, 6].

Next, to simplify notation and intuit computational implementation, we rewrite the summation to a vectorized form through concatenation. First we define the term Ω_s that encapsulates all the interaction matrices Ω_s^{md} as

$$\Omega_s = \begin{bmatrix}\Omega_s^{m0} & \Omega_s^{m1} & \Omega_s^{m2} & \cdots & \Omega_s^{mg}\end{bmatrix}, \tag{20}$$

where each Ω_s^{md} corresponds to the individual interaction matrices defined in 19. Furthermore, we introduce the term $g(\mathbf{r}) \in \mathbb{R}^L$ as a function of $\mathbf{r}$ that, in our case of multiplicative interactions, contains all the powers of $\mathbf{r}$, such that

$$g(\mathbf{r}) = \begin{bmatrix}\mathbf{r} & \mathbf{r}^{\otimes 2} & \ldots & \mathbf{r}^{\otimes g}\end{bmatrix}, \tag{21}$$

This term represents all the possible multiplicative interactions in $\mathbf{r}$, from $\mathbf{r}^{\otimes 1}$ to $\mathbf{r}^{\otimes g}$, and L is of length $\sum_{d=0}^{g} N^d$. This allows the expression of the sum in the compact form,

$$\sum_{d=0}^{g} \Omega_s^{md}\mathbf{r}^{\otimes d} =: \Omega_s g(\mathbf{r}), \tag{22}$$

which leads to the resulting expression for the voltage equations

$$\dot{\mathbf{v}} = -\lambda\mathbf{v} + \Omega_f\mathbf{o} + \Omega_s g(\mathbf{r}). \tag{23}$$

Note that $g(\mathbf{r})$ could consist of other types of non-linear interactions as well, depending on the method at hand and the capabilities of the neurons in the network [5].

Kronecker formalism

Multiplicative interactions in the SCN framework depend on the use of the Kronecker product. The $\otimes$ symbol represents the Kronecker product, of which the operation between two matrices is given by

$$\mathbf{A} \otimes \mathbf{B} = \begin{bmatrix} a_{11}\mathbf{B} & \ldots & a_{1n}\mathbf{B} \\ \vdots & \ddots & \vdots \\ a_{m1}\mathbf{B} & \ldots & a_{mn}\mathbf{B} \end{bmatrix}. \tag{24}$$

An interesting property of the Kronecker product is the mixed-product property, given by

$$(\mathbf{A} \otimes \mathbf{B})(\mathbf{C} \otimes \mathbf{D}) = (\mathbf{AC} \otimes \mathbf{BD}), \tag{25}$$

which is allowed in the case that the matrix products $\mathbf{AC}$ and $\mathbf{BD}$ exist. As in [4], we will often use the notation $\mathbf{D}^{\otimes d}$ to denote the d-fold Kronecker product of $\mathbf{D}$ with itself; i.e. if $d = 3$, then $\mathbf{D}^{\otimes 3} = \mathbf{D} \otimes \mathbf{D} \otimes \mathbf{D}$. Summarized biological support and in-depth elaboration for the use of multiplicative interactions can be found in [4]

Sampling using SCNs

The classical SCN formulation [1,3] is given by

$$\dot{\mathbf{v}} = -\lambda \mathbf{v} + \mathbf{D}^{\mathsf{T}}(\dot{\mathbf{z}} + \lambda \mathbf{z}) - \mathbf{D}^{\mathsf{T}}\mathbf{D}\mathbf{o}. \tag{26}$$

On the other hand, the general form of standard Langevin Dynamics is given by

$$d\mathbf{z}(t) = -\frac{1}{\tau_s}\nabla U(\mathbf{z})dt + \sqrt{\frac{2}{\tau_s}}d\eta(t), \tag{27}$$

where $U(\mathbf{z})$ is the log-probability density function (PDF), τ_s is the timescale of the sampling performed by the network - in the main text and Methods set to $\tau_s = 1$ -, and $\eta \sim \mathcal{N}(0, \mathbf{I})$ is Brownian noise. Specifically, we consider PDFs belonging to the exponential family,

$$P(\mathbf{z}) = e^{-U(\mathbf{z})}. \tag{28}$$

The Langevin Dynamics 27 can be formally plugged into the membrane voltage equation 26, as in [2,7], to obtain

$$d\mathbf{v} = \left[-\lambda \mathbf{v} + \mathbf{D}^{\mathsf{T}}\left(-\frac{1}{\tau_s}\nabla U(\mathbf{z}) + \lambda \mathbf{z}\right) - \mathbf{D}^{\mathsf{T}}\mathbf{D}\mathbf{o}\right]dt + \mathbf{D}^{\mathsf{T}}\sqrt{\frac{2}{\tau_s}}d\eta(t). \tag{29}$$

Lastly we use the assumption that $\mathbf{z} \approx \hat{\mathbf{z}}$,

$$d\mathbf{v} = \left[-\lambda \mathbf{v} + \mathbf{D}^{\mathsf{T}}\left(-\frac{1}{\tau_s}\nabla U(\hat{\mathbf{z}}) + \lambda \hat{\mathbf{z}}\right) - \mathbf{D}^{\mathsf{T}}\mathbf{D}\mathbf{o}\right]dt + \mathbf{D}^{\mathsf{T}}\sqrt{\frac{2}{\tau_s}}d\eta(t). \tag{30}$$

For the Gaussian distribution, the gradient of the energy function will be linear in $\mathbf{z}$ and consequently also linear in $\mathbf{r}$ [2,7]. However, as is, the current formulation does not accommodate more complex distributions, as the energy function will contain non-linear interactions between $\mathbf{r}$.

Sampling polynomial-moment exponential family distributions

Consider $P(\mathbf{z}) \propto e^{-U(\mathbf{z})}$ for $\mathbf{z} \in \mathbb{R}^M$; if $U(\mathbf{z})$ is a polynomial with maximum degree $g+1$, $\nabla_{\mathbf{z}} U$ has maximum degree g and can always be written in the form

$$\nabla_{\mathbf{z}} U(\mathbf{z}) = \sum_{d=0}^{g} \mathbf{A}_d \mathbf{z}^{\otimes d}, \tag{31}$$

where $\mathbf{A}_d \in \mathbb{R}^{M \times M^d}$ is the matrix of coefficients for the degree-d terms in ∇U, and we define $\mathbf{z}^{\otimes d}$ as above. Then we can rewrite 30 as

$$d\mathbf{v} = \left[-\lambda \mathbf{v} + \mathbf{\Omega_f}\mathbf{o} - \frac{1}{\tau_s}\sum_{d=0}^{g} \mathbf{\Omega_s^{md}}\mathbf{r}^{\otimes d}\right]dt + \sqrt{\frac{2}{\tau_s}}\mathbf{D}^{\mathsf{T}}d\eta(t), \tag{32}$$

where

$$\mathbf{\Omega_f} = -\mathbf{D}^\mathsf{T}\mathbf{D},$$
$$\mathbf{\Omega_s^{m1}} = \mathbf{D}^\mathsf{T}(\mathbf{A}_1 - \lambda\mathbf{I})\mathbf{D},$$
$$\mathbf{\Omega_s^{md}} = \mathbf{D}^\mathsf{T}\mathbf{A}_d\mathbf{D}^{\otimes d} \text{ for } d \in \{0, 2, 3, ..., g\}.$$

Notice that we must ensure that the polynomial $U(\mathbf{z})$ is well behaved, i.e. that $\int e^{U(\mathbf{z})}\, d\mathbf{z} < \infty$. This is true, for example, in $1-D$ for polynomials $U(\mathbf{z})$ with a maximum degree that is even, and when the highest order term has a positive coefficient (e.g. $U(z) = z^2$).

Using the same notation introduced in 22, we can rewrite 32 as:

$$\dot{\mathbf{v}} = -\lambda\mathbf{v} + \mathbf{\Omega}_s\mathbf{g}(\mathbf{r}) + \mathbf{\Omega}_f\mathbf{o} + \boldsymbol{\eta}. \tag{33}$$

Sampling-based inference of stimuli through Poisson sensory neurons

A latent variable is encoded by noisy spiking neurons, and the spiking is then used by downstream regions for further computation. One such computation is the inference of the latent variable, which will be inherently uncertain due to the encoding noise. One way to deal with this uncertainty is to sample from the posterior distribution over latent variables, a process called sampling-based inference [2, 8].

Starting with a single Poisson neuron with firing rate kernel ϕ dependent on the incoming stimulus $\mathbf{z}$:

$$P(\sigma|z) = \frac{\phi(z)^\sigma}{\sigma!}e^{-\phi(z)}. \tag{34}$$

The posterior distribution over latent variables, assuming a flat prior, can be obtained with Bayes' rule simply as

$$P(z|\sigma) \propto P(\sigma|z) \tag{35}$$

Here, we want to sample from $P(z|\sigma)$ using Langevin Dynamics, which is, formally:

$$dz(t) = -\nabla_z \log P(z|\sigma)dt + \sqrt{\frac{2}{\tau_s}}d\eta(t). \tag{36}$$

Extending to multiple neurons and high$-D$ variables $\mathbf{z}$, we can write

$$P(\mathbf{z}|\sigma) \propto \exp^{-U(\mathbf{z})} \Longleftrightarrow U(\mathbf{z}) = -\log P(\mathbf{z}|\sigma), \tag{37}$$

where

$$P(\mathbf{z}|\sigma) \propto \prod_i^N \exp^{-\phi_i(\mathbf{z})} \frac{\phi_i(\mathbf{z})^{\sigma_i}}{\sigma_i!}, \tag{38}$$

such that

$$U(\mathbf{z}) = -\log P(\mathbf{z}|\sigma) = \sum_i^N \phi_i(\mathbf{z}) - \sigma_i \ln\left(\phi_i(\mathbf{z})\right) + \ln(\sigma_i)! \tag{39}$$

Consequently,

$$\begin{aligned} \nabla_\mathbf{z} U(\mathbf{z}) &= \sum_i^N \nabla_\mathbf{z}\phi_i(\mathbf{z}) - \sigma_i \nabla_\mathbf{z} \ln \phi_i(\mathbf{z}) \\ &= \sum_i^N \nabla_\mathbf{z}\phi_i(\mathbf{z}) \left[1 - \frac{\sigma_i}{\phi_i(\mathbf{z})}\right]. \end{aligned} \tag{40}$$

Once a kernel is chosen, 40 can be plugged into 30 to obtain a SCN that performs sampling from the posterior distribution over latent variables $\mathbf{z}$, which is read out through $\hat{\mathbf{z}} = \mathbf{D}\mathbf{r}$:

$$\begin{aligned} d\mathbf{v} &= \left[-\lambda \mathbf{v} + \mathbf{D}^\mathsf{T}\left(-\frac{1}{\tau_s}\nabla U(\hat{\mathbf{z}}) + \lambda\hat{\mathbf{z}}\right) - \mathbf{D}^\mathsf{T}\mathbf{D}\mathbf{o}\right]dt + \mathbf{D}^\mathsf{T}\sqrt{\frac{2}{\tau_s}}d\eta(t) \\ &= \left[-\lambda \mathbf{v} + \mathbf{D}^\mathsf{T}\left(-\frac{1}{\tau_s}\sum_i^N \nabla_\mathbf{z}\phi_i(\mathbf{z})\left[1 - \frac{\sigma_i}{\phi_i(\mathbf{z})}\right] + \lambda\hat{\mathbf{z}}\right) - \mathbf{D}^\mathsf{T}\mathbf{D}\mathbf{o}\right]dt + \mathbf{D}^\mathsf{T}\sqrt{\frac{2}{\tau_s}}d\eta(t) \end{aligned} \tag{41}$$

In the following, we will see the different forms of this equation for different choices of firing rate kernel ϕ, but in principle, any positive function with continuous derivative will work.

Exponential Kernel

Choosing an exponential firing rate kernel with projection vectors β,

$$\phi_i(\mathbf{z}) = e^{\beta_i \mathbf{z}} \rightarrow \nabla_\mathbf{z}\phi_i(\mathbf{z}) = \beta_i\phi_i(\mathbf{z}), \tag{42}$$

such that

$$\nabla_\mathbf{z} U(\mathbf{z}) = \sum_i^N \beta_i(e^{\beta_i\mathbf{z}} - \sigma_i). \tag{43}$$

we find Langevin Dynamics on $\mathbf{z}$ evolve according to

$$d\mathbf{z}(t) = -\frac{\beta}{\tau_s}\left(e^{\beta\mathbf{z}} - \sigma\right)dt + \sqrt{\frac{2}{\tau_s}}d\eta(t). \tag{44}$$

We can now plug this into the membrane voltage equation 41, discerning between the spiking of the Poisson neurons σ and the spiking of the mSCN $\mathbf{o}$, and using the fact that $\mathbf{z} \approx \hat{\mathbf{z}}$,

$$d\mathbf{v} = \left[-\lambda\mathbf{v} - \frac{1}{\tau_s}\mathbf{D}^\mathsf{T}\beta\left(e^{\beta\hat{\mathbf{z}}} - \sigma\right) + \lambda\mathbf{D}^\mathsf{T}\hat{\mathbf{z}} - \mathbf{D}^\mathsf{T}\mathbf{D}\mathbf{o}\right]dt + \sqrt{\frac{2}{\tau_s}}\mathbf{D}^\mathsf{T}d\eta(t). \tag{45}$$

Using $\hat{\mathbf{z}} = \mathbf{D}\mathbf{r}$,

$$d\mathbf{v} = \left[-\lambda\mathbf{v} - \frac{1}{\tau_s}\mathbf{D}^\mathsf{T}\beta\left(e^{\beta\mathbf{D}\mathbf{r}} - \sigma\right) + \lambda\mathbf{D}^\mathsf{T}\mathbf{D}\mathbf{r} - \mathbf{D}^\mathsf{T}\mathbf{D}\mathbf{o}\right]dt + \sqrt{\frac{2}{\tau_s}}\mathbf{D}^\mathsf{T}d\eta(t). \tag{46}$$

The exponential term can be approximated by its truncated Taylor series and rewritten with multiplicative synapses as in 32.

Linear Kernel

For a linear firing rate kernel with base firing rate r_0,

$$\phi_i(\mathbf{z}) = A_i\mathbf{z} + r_0 \rightarrow \nabla_\mathbf{z}\phi_i(\mathbf{z}) = A_i. \tag{47}$$

Plugged into 40,

$$\nabla U(\mathbf{z}) = \sum_i^N A_i\left(1 - \frac{\sigma_i}{A_i\mathbf{z} + r_0}\right). \tag{48}$$

Using the simple approximation $\frac{1}{1+x} \approx 1 - x + x^2$,

$$\nabla U(\mathbf{z}) = \sum_i A_i\left[1 - \frac{\sigma_i}{r_0}\left(1 - \frac{A_i\cdot\mathbf{z}}{r_0} + \frac{(A_i\cdot\mathbf{z})^2}{r_0^2}\right)\right] \tag{49}$$

If we use $\mathbf{z} \approx \hat{\mathbf{z}} = \mathbf{D}\mathbf{r}$, then we can rewrite $(A_i \cdot \mathbf{z})^2$ as

$$(A_i \cdot \mathbf{z})^2 = (A_i \cdot \mathbf{D}\mathbf{r})^2 = A_i^{\otimes 2}(\mathbf{D}\mathbf{r})^{\otimes 2} = A_i^{\otimes 2}\mathbf{D}^{\otimes 2}\mathbf{r}^{\otimes 2}. \tag{50}$$

Plugging this into the equation for ∇U,

$$\nabla U(\mathbf{z}) = \sum_i A_i \left[1 - \frac{\sigma_i}{r_0} + \frac{\sigma_i A_i^\intercal \mathbf{D}}{r_0^2}\mathbf{r} - \frac{\sigma_i (A_i \otimes A_i)(\mathbf{D} \otimes \mathbf{D})}{r_0^3}\mathbf{r}^{\otimes 2} \right] \tag{51}$$

Writing in vectorized form and plugging into 41, one finds

$$d\mathbf{v} = \Bigg[-\lambda \mathbf{v} - \frac{1}{\tau_s}\mathbf{D}^\intercal \mathbf{A}^\intercal \left(1 - \frac{\sigma \mathbf{A}\mathbf{r}}{r_0} + \frac{\sigma \mathbf{A}^{\otimes 2}\mathbf{D}^{\otimes 2}\mathbf{r}^{\otimes 2}}{r_0^2} \right) \tag{52}$$

$$+ \lambda \mathbf{D}^\intercal \mathbf{D}\mathbf{r} - \mathbf{D}^\intercal \mathbf{D}\mathbf{o} \Bigg] dt + \sqrt{\frac{2}{\tau_s}}\mathbf{D}^\intercal d\eta(t) \tag{53}$$

This derivation can be extended to arbitrary precision by including more Taylor expansion terms, up to K:

$$\begin{aligned} d\mathbf{v} &= \left(-\lambda \mathbf{v} + \lambda \mathbf{D}^\intercal \mathbf{D}\mathbf{r} - \mathbf{D}^\intercal \mathbf{D}\mathbf{o} \right) dt \\ &\quad - \frac{1}{\tau_s}\mathbf{D}^\intercal \mathbf{A}^\intercal \left[1 - \frac{\sigma}{r_0}\sum_{n=0}^{K} \left(-\frac{1}{r_0} \right)^n \mathbf{A}^{\otimes n}\mathbf{D}^{\otimes n}\mathbf{r}^{\otimes n} \right] dt \\ &\quad + \sqrt{\frac{2}{\tau_s}}\mathbf{D}^\intercal d\eta(t), \end{aligned} \tag{54}$$

Logistic kernel

For a logistic firing rate kernel

$$\phi_i(\mathbf{z}) = (1 + \exp(-A_i \mathbf{z}))^{-1} \rightarrow \nabla_{\mathbf{z}} \phi_i(\mathbf{z}) = \frac{A_i \exp(-A_i \mathbf{z})}{(1 + \exp(-A_i \mathbf{z}))^2}. \tag{55}$$

Plugged into 40,

$$\nabla U(\mathbf{z}) = \sum_i^N \frac{A_i \exp(-A_i \mathbf{z})}{(1 + \exp(-A_i \mathbf{z}))^2} \left[1 - \sigma_i (1 + \exp(A_i \mathbf{z})) \right]. \tag{56}$$

Let's consider for simplicity the case where $\mathbf{z}$ is $1 - D$; similar approximations can be achieved in higher dimensions. The term $\nabla_z \phi_i(z)$ is well approximated, within a neighborhood of $z \approx 0$, by a parabola:

$$\nabla_z \phi_i(z) \approx -A_i\left(\frac{1}{4} - \frac{A_i^2 z^2}{32}\right), \tag{57}$$

therefore we consider

$$\begin{aligned} \nabla U(z) &\approx \sum_i^N -A_i \left(\frac{1}{4} + \frac{A_i^2 z^2}{32} \right) \left[1 - \sigma_i (1 + \exp(A_i \mathbf{z})) \right] \\ &\approx \sum_i^N -A_i \left(\frac{1}{4} + \frac{A_i^2 z^2}{32} \right) \left[1 - \sigma_i (2 + A_i z + A_i^2 z^2 + \cdots + A_i^K z^K) \right]. \end{aligned} \tag{58}$$

This is a polynomial of order $K + 2$, and can be easily implemented into 41.

Different noise models

Other discrete probability distributions that can be chosen as noise models and implemented in 41 upon choosing appropriate approximations and/or synaptic nonlinearities:

Bernoulli: Suppose we are dealing with independent binary neurons σ_i with firing probability sigmoid $f_i(\mathbf{z}) \in [0,1]$:

$$P(\sigma_i|\mathbf{z}) = f_i(\mathbf{z})^{\sigma_i}(1 - f_i(\mathbf{z}))^{1-\sigma_i} \tag{59}$$

Suppose that $f_i(\mathbf{z}) = \frac{1}{1+\exp(-\beta_i^\intercal \mathbf{z})}$. Taking the gradient of $\log f(\mathbf{z})$ we have the simple relationship $\nabla_{\mathbf{z}} \log f(\mathbf{z}) = \beta(1 - f(\mathbf{z}))$, hence

$$\nabla_{\mathbf{z}} \log P(\sigma_i|\mathbf{z}) = \sigma_i\beta_i(1 - f_i(\mathbf{z})) + (1 - \sigma_i)\beta_i f_i(\mathbf{z})) \tag{60}$$

Considering now a population $\sigma = \{\sigma_1, \ldots, \sigma_k\}$ where $P(\sigma|\mathbf{z}) = \prod_i P(\sigma_i|\mathbf{z})$,

$$\nabla_{\mathbf{z}} \log P(\sigma|\mathbf{z}) = \sum_i \sigma_i\beta_i(1 - f_i(\mathbf{z})) + (1 - \sigma_i)\beta_i f_i(\mathbf{z})) \tag{61}$$

Binomial: If each neuron is binomial, as in $\sigma_i \in \{0, 1, \ldots, n\}$ and

$$P(\sigma_i|\mathbf{z}) = \binom{n}{\sigma_i} f_i(\mathbf{z})^{\sigma_i}(1 - f_i(\mathbf{z}))^{n-\sigma_i} \tag{62}$$

there follow similar derivations as above, and

$$\nabla_{\mathbf{z}} \log P(\sigma|\mathbf{z}) = \sum_i \sigma_i\beta_i(1 - f_i(\mathbf{z})) + (n - \sigma_i)\beta_i f_i(\mathbf{z})) \tag{63}$$

Geometric: if $\sigma_i \in \{0, 1, \ldots\}$, where $P(\sigma_i|\mathbf{z}) = f_i(\mathbf{z})(1 - f_i(\mathbf{z}))^{\sigma_i}$, then

$$\nabla_{\mathbf{z}} \log P(\sigma|\mathbf{z}) = \sum_i \beta_i(1 - f_i(\mathbf{z})) + \sigma_i\beta_i f_i(\mathbf{z})) \tag{64}$$

Skewness of posterior

Assuming each input neuron i follows Poisson noise with firing rate kernel ϕ_i dependent on the incoming stimulus z^*, a simple flat prior means that the posterior will have form

$$P(z|\sigma) \propto \prod_i \frac{\phi_i(z)^{\sigma_i}}{\sigma_i!} e^{-\phi_i(z)}. \tag{65}$$

In regimes with low spike counts and nonlinear, monotonous transfer function ϕ_i, such as ReLU, exponential, or sigmoids, the Poisson posterior over z will generally be skewed. This skewness is not guaranteed in all possible models, but it is the generic case for realistic tuning curves and firing rates. Intuitively, a single Poisson neuron's posterior will behave as $\propto \phi^\sigma e^{-\phi}$, which is highly asymmetric for any $\sigma \geq 0$. The exact shape of the full distribution will depend on ϕ_i. Let's unpack one example to illustrate: $\phi_i(z) = \exp(\beta_i z)$. The log-likelihood is $l(z) \propto \sum_i \sigma_i(\beta_i z) - \exp(\beta_i z)$. We can find the mode of the distribution by simply finding the root of $l'(z) = 0$. The Taylor expansion of the log-density for a symmetric distribution should have odd moments equal to zero around the mode: $l'''(z) = -\sum_i \beta_i^3 \exp(\beta_i z)$. This is $\neq 0$ in general, unless the tuning functions are arranged in a special way. Moreover, this also shows that $l'''(z)$ is dominated by terms such that $\beta_i z^* > 0$, showing that the skewness is generally negative for positive z^*, and positive for negative z^*.

Separation of timescales, and temporal integration to estimate moments

To deal with dynamically encoded stimuli, we assume that the neural dynamics are at least one order of magnitude faster than the stimulus dynamics; this could be justified by observing that animals interact with their surroundings with actions often spanning hundreds of milliseconds, while the average membrane timescales in the cortex are on the order of tens of milliseconds; or by observing the millisecond precision of stimulus-related spikes in the visual system of multiple organisms for natural stimuli with much slower timescales [9, 10]. This allows for the exploration of the posterior distribution over dynamic stimuli online, through a moving average or a similar filter.

Extension to a Torus Manifold

In the HD model, the latent state was represented in two Cartesian coordinates (x_t, y_t) corresponding to a single angular variable θ_t, and the prior was chosen to constrain the dynamics (softly) to the circular manifold $x_t^2 + y_t^2 = \rho^2$. Here we show how the same construction extends to a two-dimensional torus, corresponding to the product manifold of two circles.

We represent the two phase variables θ_t and ϕ_t in 4D Cartesian form:

$$x_t = \cos\theta_t, \qquad y_t = \sin\theta_t, \qquad \chi_t = \cos\phi_t, \qquad \upsilon_t = \sin\phi_t, \tag{66}$$

and collect them in the 4D vector $z_t = \big(x_t, y_t, \chi_t, \upsilon_t\big)^T \in \mathbb{R}^4$. The SCN readout then generalizes as $z_t \approx Dr_t$ with $D \in \mathbb{R}^{N\times 4}$.

For a single circle, the prior introduced in a previous paragraph enforced a soft attraction towards $x^2 + y^2 = \rho^2$. A toroidal attractor is obtained by summing two circular priors, one for each phase dimension:

$$U_{\text{torus}}(z_t) = \gamma_1\big(x_t^2 + y_t^2\big)\big(x_t^2 + y_t^2 - 2\rho_1^2\big) + \gamma_2\big(\chi_t^2 + \upsilon_t^2\big)\big(\chi_t^2 + \upsilon_t^2 - 2\rho_2^2\big) \tag{67}$$

where $\gamma_1, \gamma_2 > 0$ determine the attraction strength and ρ_1, ρ_2 are the radii of the two circular factors. U_{torus} is zero on the circles of radius ρ_1 and ρ_2, and increases smoothly away from the manifold.

Next, following the same Langevin approximation as in the HD case, the gradient of the toroidal prior gives two independent attractor forces:

$$\nabla_{x,y} U_{\text{torus}} = \gamma_1 \begin{bmatrix} x_t \\ y_t \end{bmatrix} \Big(x_t^2 + y_t^2 - 2\rho_1^2\Big), \tag{68}$$

$$\nabla_{\chi,\upsilon} U_{\text{torus}} = \gamma_2 \begin{bmatrix} \chi_t^2 \\ \upsilon_t^2 \end{bmatrix} \Big(\chi_t^2 + \upsilon_t^2 - 2\rho_2^2\Big). \tag{69}$$

Finally, to sample on the torus, we consider two populations σ_t^1 and σ_t^2 encoding noisy samples of the two phase velocities $\omega_t^{(1)}$ and $\omega_t^{(2)}$. Using the small-angle approximation, the discrete-time sampler becomes

$$\begin{bmatrix} x_{t+1} \\ y_{t+1} \end{bmatrix} = \begin{bmatrix} x_t \\ y_t \end{bmatrix} + dt\,\beta_1 \begin{bmatrix} y_t \\ -x_t \end{bmatrix} \big(\sigma_t^1 - 1\big) - dt\,\gamma_1 \begin{bmatrix} x_t \\ y_t \end{bmatrix} \big(x_t^2 + y_t^2 - 2\rho_1^2\big) + \sqrt{2dt}\,\eta_t^1 \tag{70}$$

$$\begin{bmatrix} \chi_{t+1} \\ \upsilon_{t+1} \end{bmatrix} = \begin{bmatrix} \chi_t \\ \upsilon_t \end{bmatrix} + dt\,\beta_2 \begin{bmatrix} \upsilon_t \\ -\chi_t \end{bmatrix} \big(\sigma_t^2 - 1\big) - dt\,\gamma_2 \begin{bmatrix} \chi_t \\ \upsilon_t \end{bmatrix} \big(\chi_t^2 + \upsilon_t^2 - 2\rho_2^2\big) + \sqrt{2dt}\,\eta_t^2 \tag{71}$$

As in the circular case, these dynamics can be embedded in a spiking network by identifying the attractor with components of the slow nonlinear interaction matrix $\Omega_s g(r)$, where Ω_s now includes the gradients derived from U_{torus}.

Convergence analysis

To better understand the overall performance and the impact of the parameters on the sampling of the SCN, we chose three distributions and sampled from them using 30 with varying parameters:

$$d\mathbf{v} = \left[-\lambda\mathbf{v} + \mathbf{D}^\mathsf{T}\left(-\frac{\epsilon}{\tau_s}\nabla U(\hat{\mathbf{z}}) + \lambda\hat{\mathbf{z}}\right) - \mathbf{D}^\mathsf{T}\mathbf{D}\mathbf{o}\right]dt + \mathbf{D}^\mathsf{T}\sqrt{\frac{2\epsilon}{\tau_s}}d\eta(t). \tag{72}$$

To appropriately test the sampling performance, we hand-picked three distributions that portrayed interesting and somewhat complicated characteristics: the simplest distribution is one-dimensional with fourth-order terms in the energy function, which results in a double-peaked distribution (S3 Fig, C, left); the second distribution is also one-dimensional but with eighth-order terms in the energy function, leading to a triple-peaked distribution (S3 Fig, C, right); lastly, we also analyzed a three-dimensional distribution with fourth-order terms in the energy function, as seen in (S2 Fig, A). Specifically, the distributions are given by respectively

$$\begin{aligned} U_1(x) &= x + x^2 - 3x^3 + x^4, \\ U_2(x) &= (0.9 * (x-0.1)^8 + 1.05 * (x-0.13)^7 - x^6 - x^5 + (x+0.15)^2)/0.4, \\ U_3(x,y,z) &= x + x^2 - 3x^3 + y^2 - 3y + x^2y^2 + xyz^2 + x^2yz + x^4 + z^4. \end{aligned} \tag{73}$$

As for the parameters, we varied the number of neurons N, the voltage leak λ, and the magnitude of the decoding matrix $D_{mag} = \|\mathbf{D}\|$. The following parameter sets were used for sampling all distributions:

$$\begin{aligned} N &= [4,\ 8,\ 16,\ 32,\ 64,\ 128,\ 200], \\ D_{mag} &= \left[\frac{1}{2},\ \frac{1}{4},\ \frac{1}{8},\ \frac{1}{16},\ \frac{1}{25},\ \frac{1}{32},\ \frac{1}{50},\ \frac{1}{64},\ \frac{1}{100}\right], \\ \lambda &= [0.1,\ 1,\ 2,\ 4,\ 8,\ 10,\ 16,\ 20,\ 32,\ 64]. \end{aligned} \tag{74}$$

Additionally, for the 1D, 4th and 8th order distributions, $D_{mag} = \left[\frac{1}{200},\ \frac{1}{400}\right]$ and $\lambda = \left[\frac{1}{2},\ \frac{1}{50},\ \frac{1}{100},\ \frac{1}{500},\ \frac{1}{1000}\right]$ were added to the parameter sets for better characterization. We ran all permutations of the parameters to see which combination would produce the best result, with 20 runs for each permutation to make sure accurate results are obtained given the stochasticity in the sampling procedure. Other relevant parameters were standardized across all runs: for the 1D distributions, nt = 10000 timesteps, dt = 0.001 time step size, $\epsilon = 10$ learning rate; for the 3D distribution, nt = 10000 timesteps, dt = 0.01 time step size, $\epsilon = 100$ learning rate. No random seed was used for simulations, so the readout matrices $\mathbf{D}$, i.e. also the interaction matrices (19), as well as the noise η were randomized in every run.

Performance calculation

To quantify the performance of the sampling, we used the $\mathcal{L}_1$ metric between the histogram bins of the ground truth distribution and the sampled empirical distribution with standardized bins,

$$L_1(b_1, b_2) = \sum_{i=1}^{n} |b_1[i] - b_2[i]| \,. \tag{75}$$

The histogram bins were standardized for each distribution to ensure validity: in the 1D 4th order case, 100 histogram bins between -3 and 5; in the 1D 8th order case, 100 bins between -1.5 and 1.5; in the 3D 4th order case, 200 bins between -4 and 4 in each dimension. It was ensured that the histograms of the ground truth distribution and empirical distribution had the same bin centers. The ground truth distribution and the empirical distributions were appropriately normalized: the ground truth distribution was normalized by dividing by the partition function through numerical integration over the entire space, and the empirical distribution was normalized using the density option in the numpy.histogramdd function.

Furthermore, as the $\mathcal{L}_1$ metric magnitude varied strongly in each distribution ensemble, i.e. the collections of all sampling runs for a specific distribution in 73, we calculate a normalized similarity score for each distribution. Within each distribution ensemble, the best $\mathcal{L}_1$ is taken as the performance baseline. Then, we compute the difference between each $\mathcal{L}_1$ value and the best (minimum) $\mathcal{L}_1$ in the dataset, effectively inverting the metric so that higher values correspond to better similarity. This ensures that a higher score represents a better match. To further normalize the scores for comparison across different distributions, we divide by the maximum value obtained after inversion, yielding a final similarity measure in the range $[0, 1]$. The transformed score is thus given by:

$$S_i = \frac{\max(\mathcal{L}_1) - \mathcal{L}_1^i}{\max(\mathcal{L}_1) - \min(\mathcal{L}_1)} \tag{76}$$

where S_i is the normalized similarity score for the ith distribution.

Table 2. Best and worst parameter sets for sampling from the distributions given by 73, visualized in S3 Fig, C,D.

2*Distribution	Parameters		
	N	D_{mag}	λ
1D, 4th order best	32	$\frac{1}{25}$	0.5
1D, 4th order worst	64	$\frac{1}{400}$	64
1D, 8th order best	16	$\frac{1}{50}$	$\frac{1}{100}$
1D, 8th order worst	32	$\frac{1}{400}$	64
3D, 4th order best	128	$\frac{1}{8}$	2
3D, 4th order worst	8	$\frac{1}{50}$	1

Results

The results of the simulations can be seen in S3 Fig. The sampled distributions for the best and worst parameter sets are visualized in S3 Fig, C, D. To see the effects of each parameter on the sampling performance individually, we determined the optimal parameter sets $\{N, D_{mag}, \lambda\}$, and varied each parameter while the other two are fixed to the optimal value; the result is visualized in S3 Fig, F.

Interpreting the result in S3 Fig, F, we see that the number of neurons N does not influence performance in the 1D case, which is sensible from the geometric perspective [11], as 2 neurons with decoding vectors spanning positive and negative values suffice for optimal performance. For 3D distributions, the number of neurons impacts performance, due to the potential lack of closure in the bounding box for low N [11, 12]. Second, the magnitude of the readout matrix $\mathbf{D}$ is most relevant to the sampling performance of the network, which shows a strong dependence on D_{mag}. Both small and large magnitudes lead to bad results, for different reasons: small decoding vectors cannot counteract the decay, while big decoding vectors are too imprecise to obtain a reasonable readout [11]. Finally, the voltage decay λ has generally negligible impact on performance. Only when $\lambda > 10$ does it affect the sampling negatively. This corresponds to a quick decay and, thus, a short memory of the voltage and readout. The network forgets the previous state and cannot properly sample the distribution. From S3 Fig, E, we see that the sampling performance is not independently modulated by the parameters D_{mag} and λ; therefore, a potential solution for the posited problem is to increase D_{mag}, as the spiking will have a larger effect on the readout and can counteract the quick decay [11], but this will generally decrease the precision of the readout.

References

1. Boerlin M, Machens CK, Denève S. Predictive Coding of Dynamical Variables in Balanced Spiking Networks. PLOS Computational Biology. 2013 Nov;9(11):e1003258. Publisher: Public Library of Science. Available from: https://journals.plos.org/ploscompbiol/article?id=10.1371/journal.pcbi.1003258. doi:10.1371/journal.pcbi.1003258.

2. Savin C, Denève S. Spatio-temporal Representations of Uncertainty in Spiking Neural Networks. In: Ghahramani Z, Welling M, Cortes C, Lawrence N, Weinberger KQ, editors. Advances in Neural Information Processing Systems. vol. 27. Curran Associates, Inc.; 2014. Available from: https://proceedings.neurips.cc/paper_files/paper/2014/file/4e2545f819e67f0615003dd7e04a6087-Paper.pdf.

3. Barrett DG, Denève S, Machens CK. Optimal compensation for neuron loss. eLife. 2016 Dec;5:e12454. Publisher: eLife Sciences Publications, Ltd. Available from: https://doi.org/10.7554/eLife.12454. doi:10.7554/eLife.12454.

4. Nardin M, Phillips JW, Podlaski WF, Keemink SW. Nonlinear computations in spiking neural networks through multiplicative synapses. Peer Community In Circuit Neuroscience. 2021 Nov:100003. ArXiv:2009.03857 [cs, q-bio]. Available from: http://arxiv.org/abs/2009.03857. doi:10.24072/pci.cneuro.100003.

5. London M, Häusser M. Dendritic computation. Annual Review of Neuroscience. 2005;28:503-32. doi:10.1146/annurev.neuro.28.061604.135703.

6. Xu Nl, Harnett MT, Williams SR, Huber D, O'Connor DH, Svoboda K, et al. Nonlinear dendritic integration of sensory and motor input during an active sensing task. Nature. 2012 Dec;492(7428):247-51. Available from: https://www.nature.com/articles/nature11601. doi:10.1038/nature11601.

7. Masset P, Zavatone-Veth J, Connor JP, Murthy V, Pehlevan C. Natural gradient enables fast sampling in spiking neural networks. In: Koyejo S, Mohamed S, Agarwal A, Belgrave D, Cho K, Oh A, editors. Advances in Neural Information Processing Systems. vol. 35. Curran Associates, Inc.; 2022. p. 22018-34. Available from: https://proceedings.neurips.cc/paper_files/paper/2022/file/8a0fd48510590071e3c129a79b8b8527-Paper-Conference.pdf.

8. Orbán G, Berkes P, Fiser J, Lengyel M. Neural Variability and Sampling-Based Probabilistic Representations in the Visual Cortex. Neuron. 2016 Oct;92(2):530-43. Available from: https://www.sciencedirect.com/science/article/pii/S0896627316306390. doi:10.1016/j.neuron.2016.09.038.

9. Simoncelli EP, Olshausen BA. Natural image statistics and neural representation. Annual Review of Neuroscience. 2001;24:1193-216. doi:10.1146/annurev.neuro.24.1.1193.

10. Butts DA, Weng C, Jin J, Yeh CI, Lesica NA, Alonso JM, et al. Temporal precision in the neural code and the timescales of natural vision. Nature. 2007 Sep;449(7158):92-5. doi:10.1038/nature06105.

11. Calaim N, Dehmelt FA, Gonçalves PJ, Machens CK. The geometry of robustness in spiking neural networks. eLife. 2022 May;11:e73276. Available from: https://doi.org/10.7554/eLife.73276. doi:10.7554/eLife.73276.

12. Denève S, Machens CK. Efficient codes and balanced networks. Nature Neuroscience. 2016 Mar;19(3):375-82. Available from: https://www.nature.com/articles/nn.4243. doi:10.1038/nn.4243.
