# Supplementary material for: Combining sampling and attractor dynamics in spiking models of head direction systems
Source: PLoS Comput Biol. 2026 Jul 30;22(7):e1014577. doi: 10.1371/journal.pcbi.1014577 (PMC13450861; doi:10.1371/journal.pcbi.1014577)
Supplement: Supplementary file 2 [file pcbi.1014577.s002.pdf]

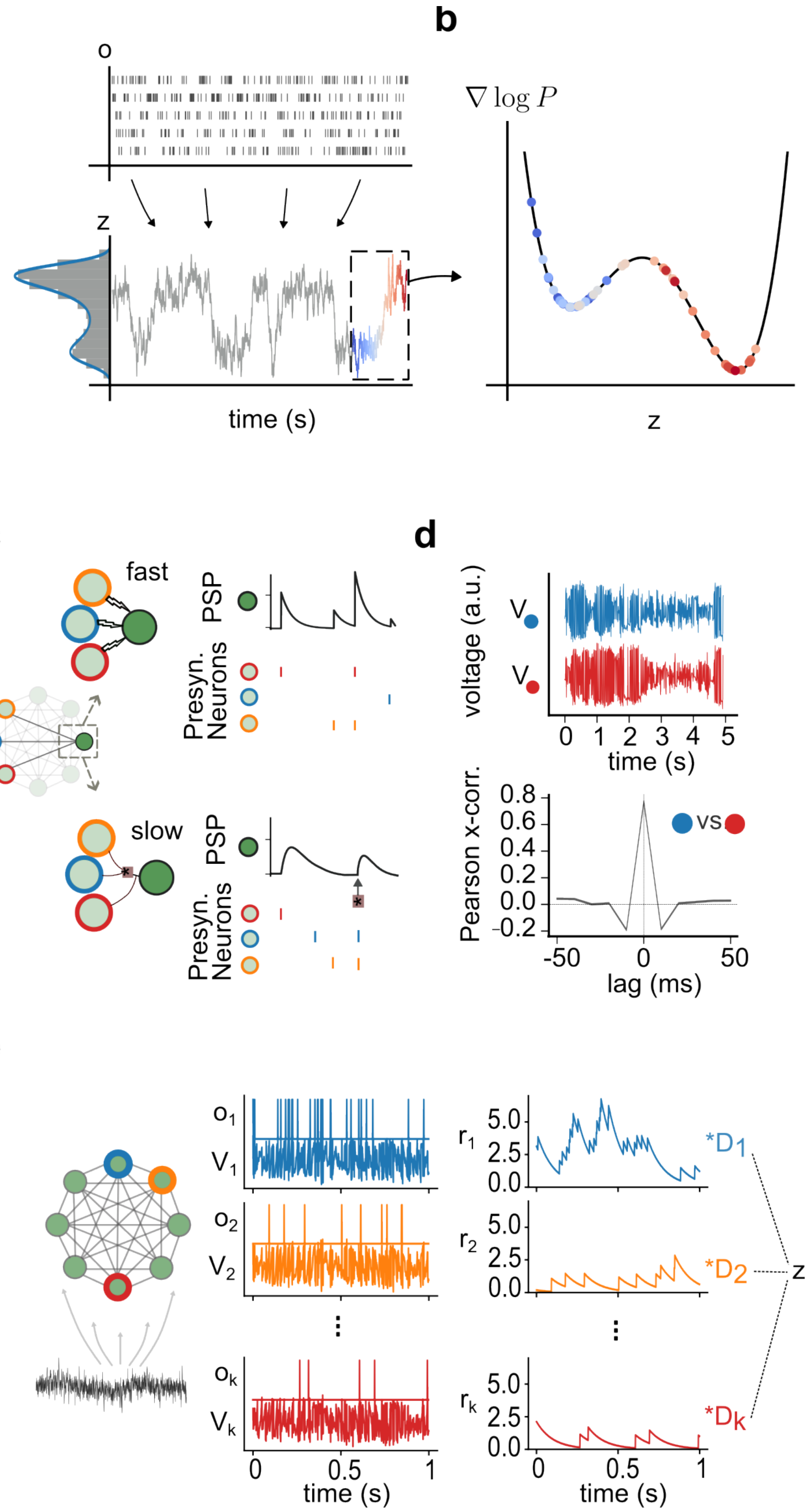

S1 Fig. Spiking neural networks can sample from arbitrary distributions through a combination of nonlinear synaptic interactions and correlated voltage fluctuations. (A) Example SCN sampling from a given bimodal distribution, a clear example of non-Gaussian distribution. Top: Spikes from the network ($\mathbf{o}$). Bottom: network readout (z_t) corresponding to samples from bimodal $P(z)$. Over time, samples from SCN approximate the original probability distribution, as seen in the histogram/density overlay. **(B)** The samples $\mathbf{z}$ explore the gradient landscape of the $\log P$ distribution; colored dots correspond to samples in the dashed box in panel **A**. **(C)** SCNs have two types of interactions between neurons: fast interactions (top) quickly propagate spikes $\mathbf{o}$ through Ω_f, and are responsible for maintaining E/I balance; slow interactions (bottom) act on a slower timescale through Ω_s, and implement both linear and nonlinear interactions. Linear interactions alone are sufficient for sampling from Gaussian distributions; generic distributions require nonlinear interactions. **(D)** Correlated voltage stochastic fluctuations enables the network to explore the distribution effectively; the Pearson cross-correlation (bottom) between two voltage traces (top) shows an interesting example of short-timescale subthreshold correlation, reminiscent of experimental studies. **(E)** Left: SCN structure with correlated voltage inputs $\boldsymbol{\eta}$. Middle: Voltage V and spikes o for 3 example neurons; spikes o contribute to the post-synaptic potentials r, which obey $\dot{r} = -\lambda r + o$. Right: readout $\mathbf{z}$ can be linearly decoded from the network activity using the decoding vectors D_is: $\mathbf{z} = \mathbf{D}\mathbf{r}$.
