# Supplementary material for: Combining sampling and attractor dynamics in spiking models of head direction systems
Source: PLoS Comput Biol. 2026 Jul 30;22(7):e1014577. doi: 10.1371/journal.pcbi.1014577 (PMC13450861; doi:10.1371/journal.pcbi.1014577)
Supplement: Supplementary file 3 [file pcbi.1014577.s003.pdf]

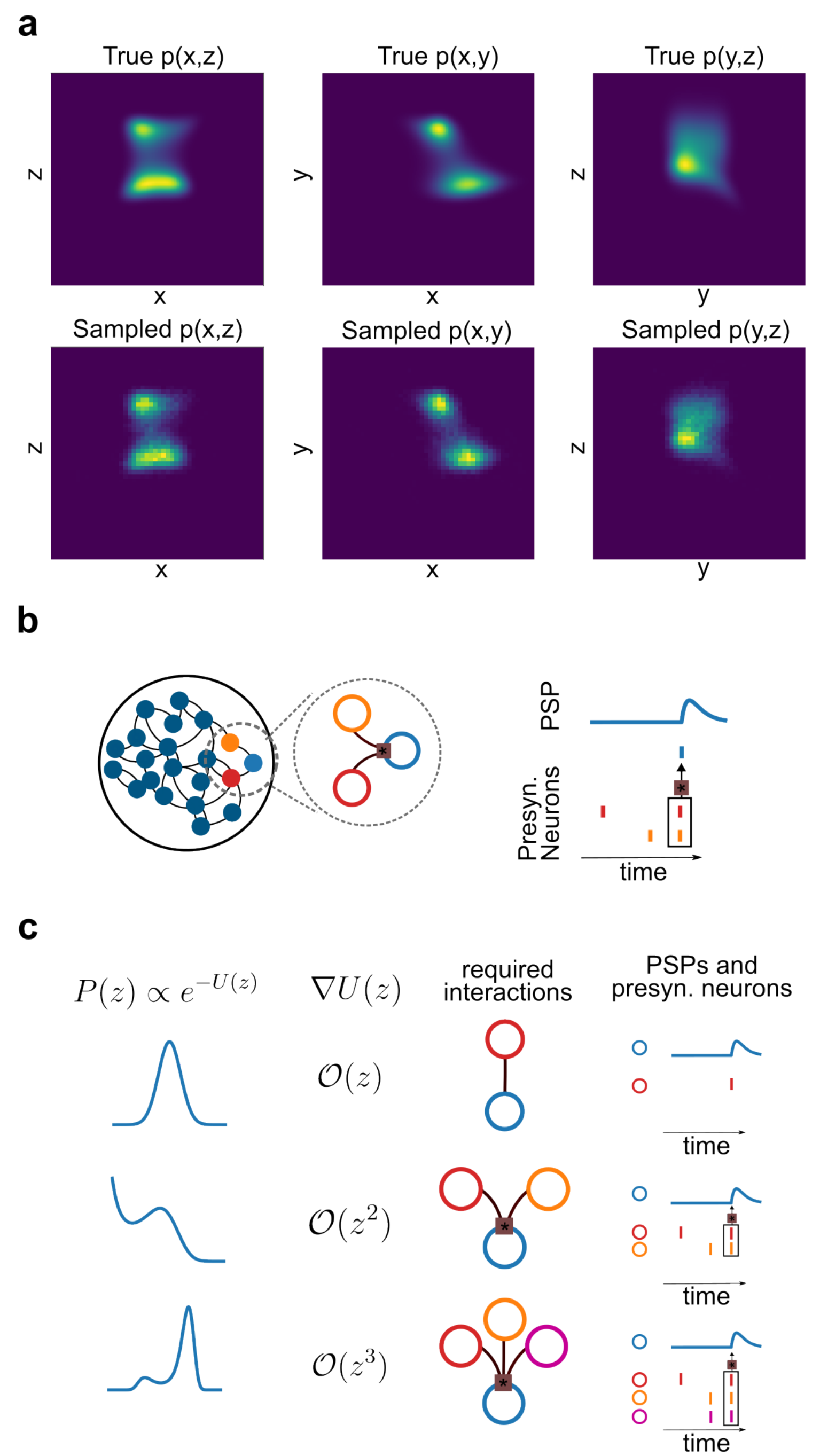

S2 Fig. Sampling from high$-D$ non-Gaussian distributions in mSCNs

(A) $3D$, 4th order polynomial moment exponential family distribution, given by $\exp(-U(x, y, z))$, where $U(x, y, z) = x + x^2 - 3x^3 + y^2 - 3y + x^2y^2 + xyz^2 + x^2yz + x^4 + z^4$. Top row: true marginal PDF, bottom: sampled marginal normalized histogram after 10000 samples. **(B)** Nonlinear polynomial dynamical systems in SCNs require multiplicative interactions among pairs of incoming inputs. The PSP of the receiving neuron i will be affected according to $\mathbf{\Omega}_{ijk} r_t^j r_t^k$, where j, k are the two presynaptic neurons, and $\mathbf{\Omega}$ is the matrix of nonlinear synaptic weights. **(C)** Higher-order polynomials require increasingly complicated multiplicative interactions. Specifically, networks have to implement $(g-1)$-th order multiplications for distributions with g-th order polynomial moments (see Extended Methods).
