# Supplementary material for: Combining sampling and attractor dynamics in spiking models of head direction systems
Source: PLoS Comput Biol. 2026 Jul 30;22(7):e1014577. doi: 10.1371/journal.pcbi.1014577 (PMC13450861; doi:10.1371/journal.pcbi.1014577)
Supplement: Supplementary file 4 [file pcbi.1014577.s004.pdf]

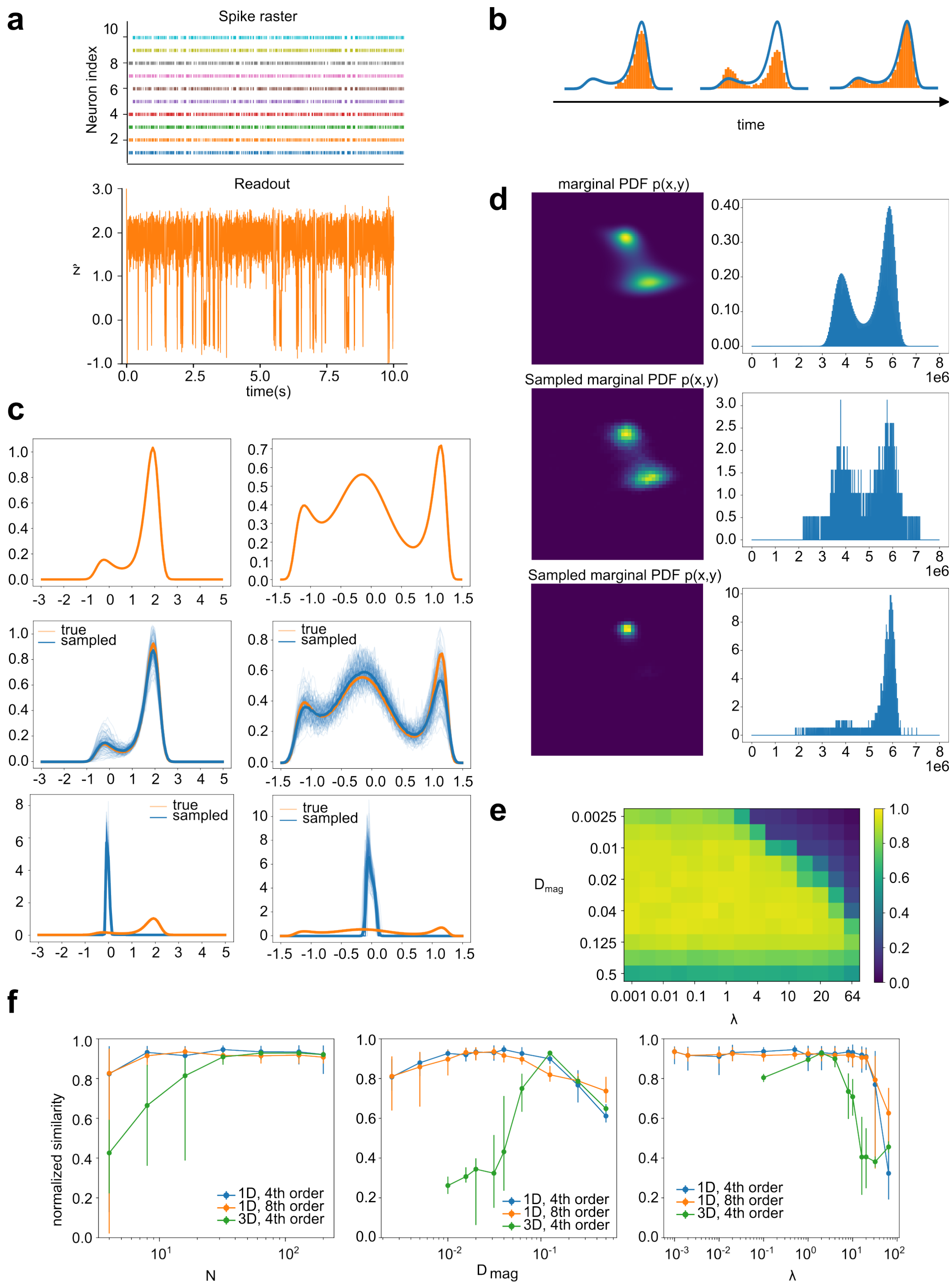

S3 Fig. Sampling behaviour and performance for various distributions

(A) Example neural activity of 10 neurons (top) and linearly read out samples (bottom) for a bimodal distribution (as in panel C, left). **(B)** Schematic of the evolution over time of an empirical histogram-based density from samples (orange), and the true density (blue). **(C)** Visualization of good and bad sampling for the 1D, 4th and 8th order distributions (left and right, respectively); true distributions (top) are sampled with best (middle) and worst (bottom) parameters as per the parameters table in the convergence analysis. Orange lines are the true distributions, thick blue line is the mean of 100 runs, each plotted as a thin blue line. **(D)** Visualization of good and bad sampling for the 3D, 4th order distribution; true distribution (top) is sampled with the best and worst parameters, shown in middle and bottom respectively, as per the parameters table in the convergence analysis. The left column showcases the marginal distributions $P(x, y)$, and the right column contains the flattened 3D histogram. **(E)** Similarity scores as a function of D_{mag} and λ for the 1D, 4th order distribution. **(F)** Similarity scores for characterizing the sampling performance for three distributions with varying parameter sets. The optimal parameter set $\{N, D_{mag}, \lambda\}$ is determined for each distribution, and consequently one parameter is varied while the other two are fixed. Blue, orange, and green line correspond to the 1D fourth order, 1D eighth order, and 3D fourth order distributions respectively. Each datapoint is the mean over 20 runs for that parameter set, and the corresponding bars span 5th to 95th percentiles. Similarity score is determined as the $\mathcal{L}_1$ distance between true and sampled distributions.
