# Supplementary material for: Combining sampling and attractor dynamics in spiking models of head direction systems
Source: PLoS Comput Biol. 2026 Jul 30;22(7):e1014577. doi: 10.1371/journal.pcbi.1014577 (PMC13450861; doi:10.1371/journal.pcbi.1014577)
Supplement: Supplementary file 5 [file pcbi.1014577.s005.pdf]

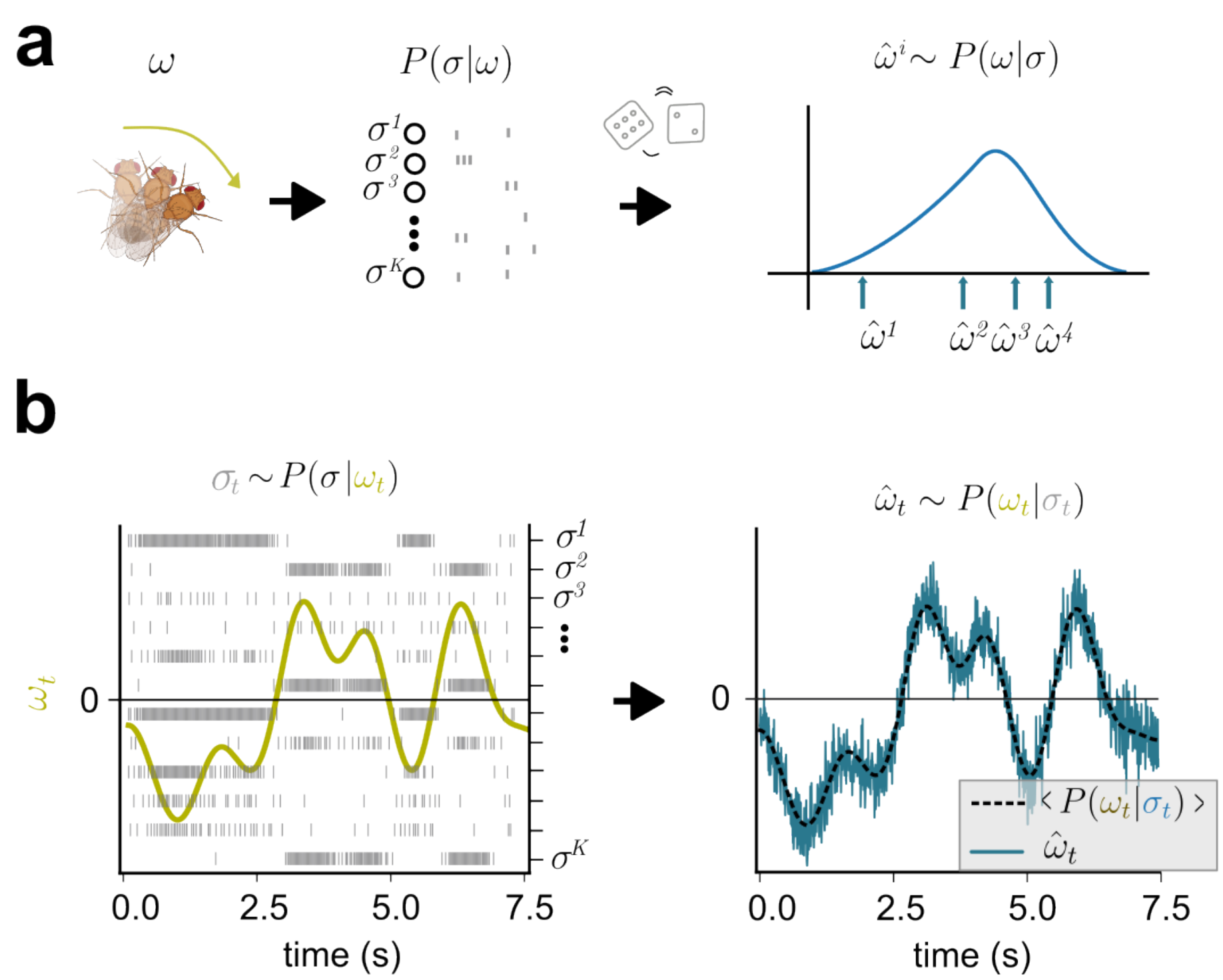

S4 Fig. Sampling-based inference of Poisson encoded angular velocity signals.
(A) Angular velocity is encoded by noisy Poisson neurons; sampling-based inference entails drawing samples from the posterior distribution over angular velocity. **(B)** SCN can perform sampling-based inference of dynamically encoded angular velocity. Left: grey spikes $\boldsymbol{\sigma}_t = \{\sigma_t^1, \ldots, \sigma_t^K\}$ encode a time-varying angular velocity signal ω_t. Right: The network readout, $\hat{\omega}_t$, draws samples from the time-varying posterior distribution $P(\omega_t|\boldsymbol{\sigma}_t)$.
