# Supplementary material for: Combining sampling and attractor dynamics in spiking models of head direction systems
Source: PLoS Comput Biol. 2026 Jul 30;22(7):e1014577. doi: 10.1371/journal.pcbi.1014577 (PMC13450861; doi:10.1371/journal.pcbi.1014577)
Supplement: Supplementary file 6 [file pcbi.1014577.s006.pdf]

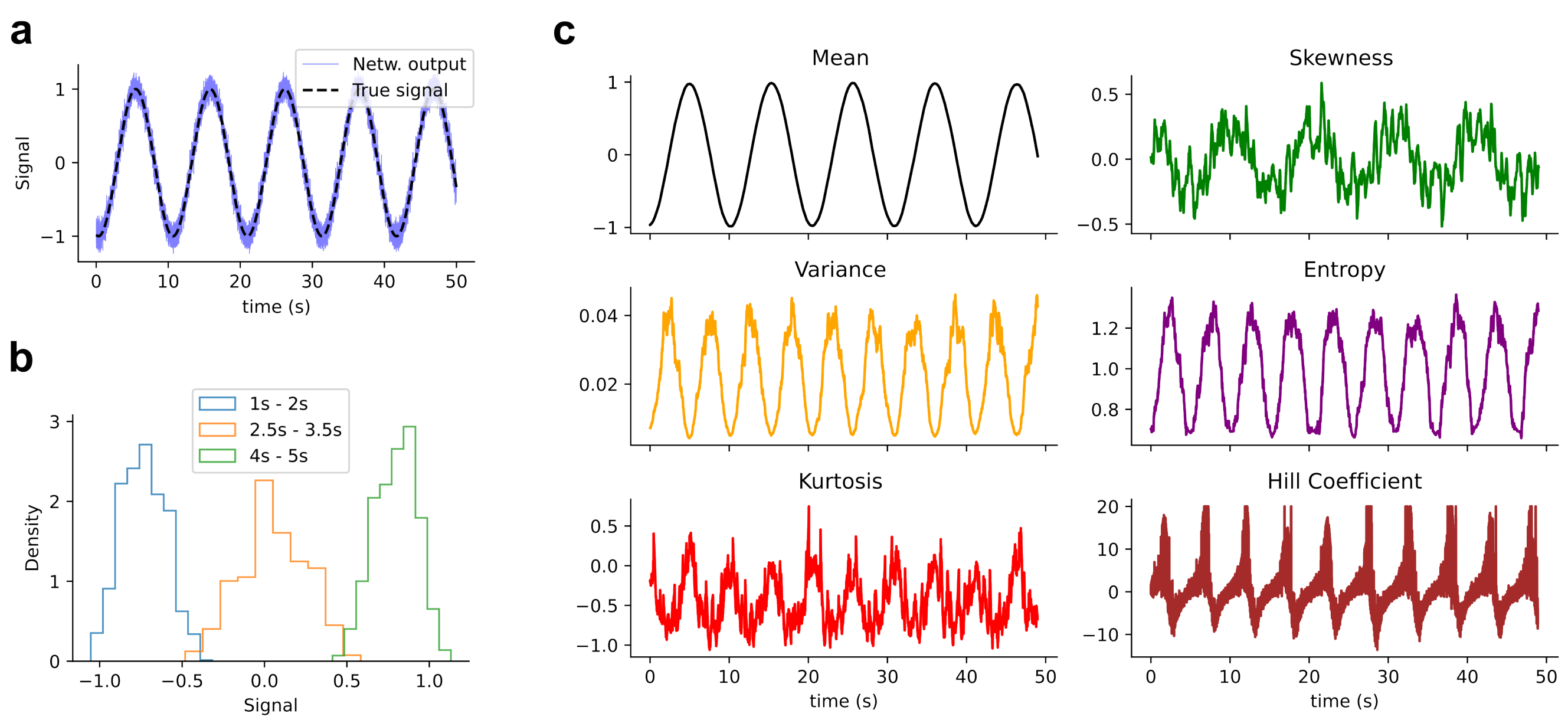

S5 Fig. Sampling-based inference allows the computation of various statistics online on time-varying stimuli posterior distributions
An oscillating external stimulus is encoded by 100 Poisson neurons for 50 seconds with a $dt = 10^{-3}$s. A SCN network with 50 neurons computes real-time sampling-based inference. **(A)** True signal (dashed black) vs network output (light blue) over 50 seconds. **(B)** We assume that a downstream reader computes various statistics on a running window of 1000 samples. Three histograms show the real-time sampling process. **(C)** Various statistics measured on the running windows of 1000 samples: first 4 central moments, entropy measured on the discretized distribution, Hill coefficient measured with a hill estimator with $k = 100$. The Hill coefficient is used to measure the thickness of a distribution's tail, which determines the probability of extreme outliers occurring.
