# Supplementary material for: Combining sampling and attractor dynamics in spiking models of head direction systems
Source: PLoS Comput Biol. 2026 Jul 30;22(7):e1014577. doi: 10.1371/journal.pcbi.1014577 (PMC13450861; doi:10.1371/journal.pcbi.1014577)
Supplement: Supplementary file 7 [file pcbi.1014577.s007.pdf]

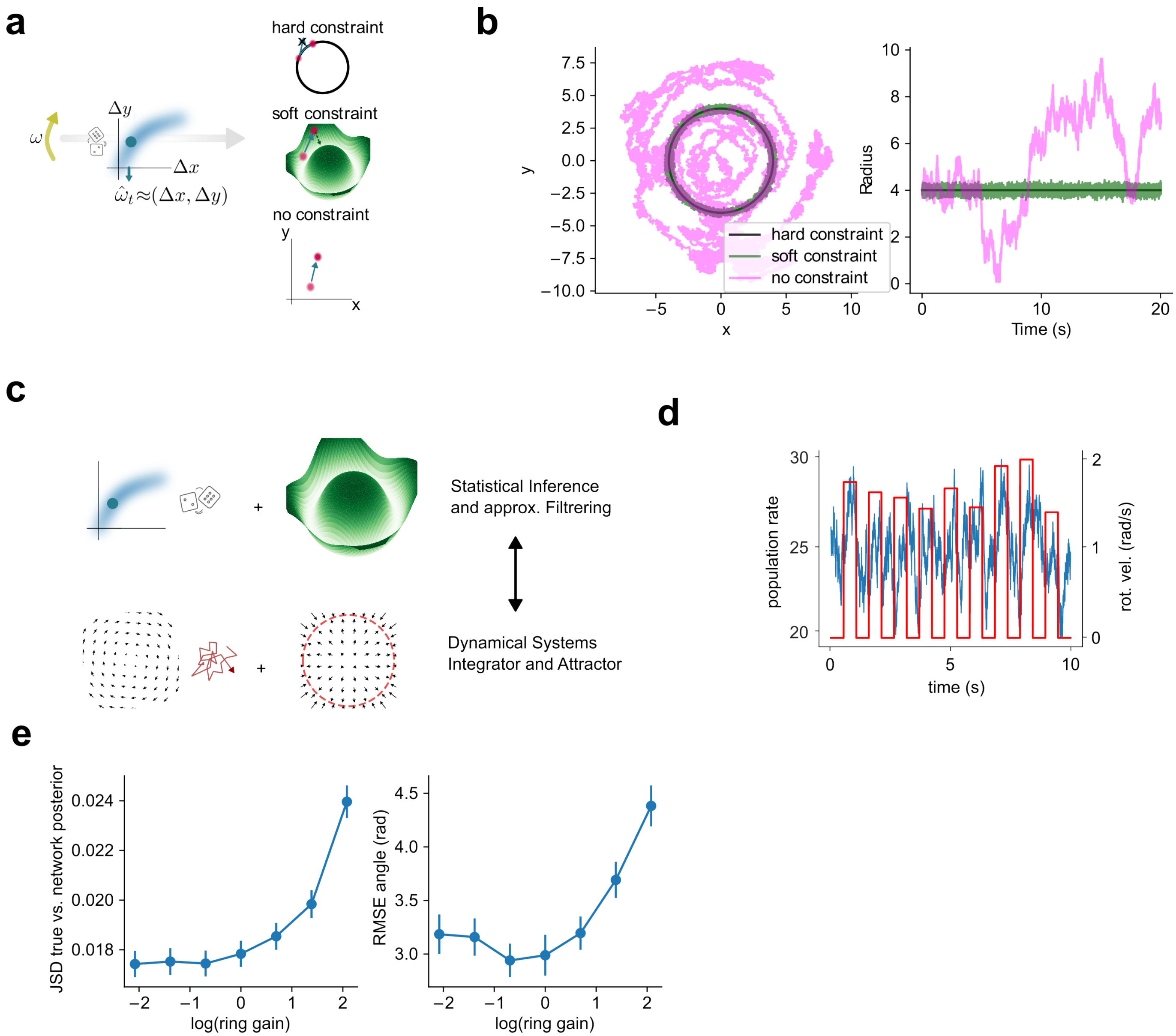

S6 Fig. Constraining $2-D$ angular representations.

(a) Left: angular velocity ω is encoded noisily, and then transformed into a $2-D$, $\Delta x, \Delta y$ HD update. Right: the HD representation in $2-D$ can be constrained in various ways; top: a hard constrain represents a renormalization, whereby $x_t, y_t = \rho \frac{x_t}{\sqrt{x_t^2+y_t^2}}, \rho \frac{y_t}{\sqrt{x_t^2+y_t^2}}$; middle: a soft constraint biases the dynamics to return towards the circle by including an energy term away from the circle, as in $(x^2+y^2)(x^2+y^2-\rho^2)$, so that the dynamics are driven towards the outside if $x^2+y^2<\rho^2$, and towards the inside if $x^2+y^2>\rho^2$; bottom: no constraints allow the dynamics to go wherever in the $2-D$ plane. **(b)** Example x, y dynamics for hard (red), soft (green), and no constraints (cyan). Notice the radius oscillations (right panel). **(c)** Writing the approximate Baysian filtering problem through Langevin dynamics results into a nonlinear dynamical system resembling a $2-D$ integrator combined with a circular limit cycle (i.e. attractor). **(d)** Average firing rate for pulses of positive angular velocity interspersed with zero velocity in a HD network as in Fig. 2. **(e)** Left: Posterior recovery accuracy, measured as Jensen-Shannon Divergence (JSD) between true posterior and network estimate. Right: Average root mean squared error angle representation for varying ring gain strength.
