# Supplementary material for: Combining sampling and attractor dynamics in spiking models of head direction systems
Source: PLoS Comput Biol. 2026 Jul 30;22(7):e1014577. doi: 10.1371/journal.pcbi.1014577 (PMC13450861; doi:10.1371/journal.pcbi.1014577)
Supplement: Supplementary file 8 [file pcbi.1014577.s008.pdf]

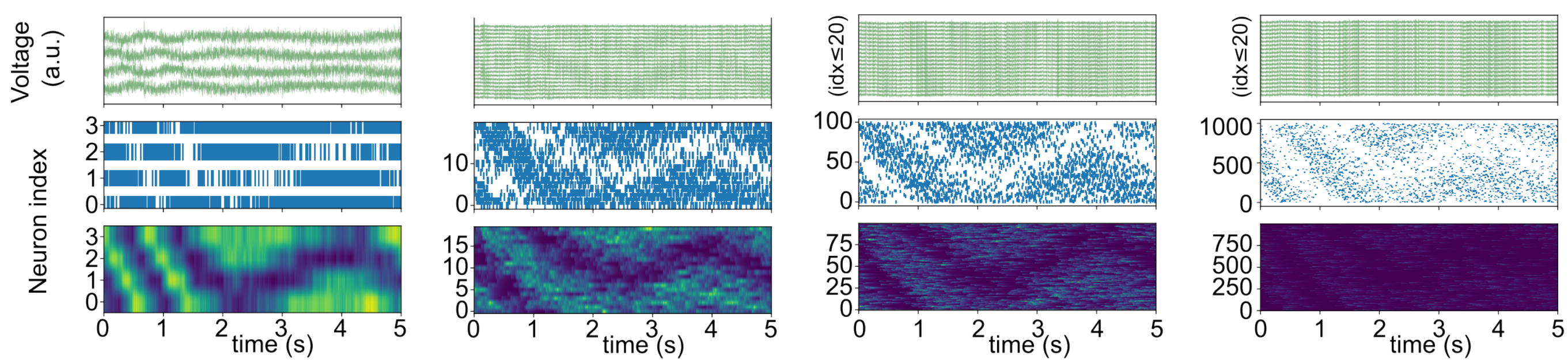

S7 Fig. HD implementation in small vs large networks. Voltage ($\mathbf{v}$), spikes (o), and filtered spikes (r) for SCN HD networks with 4, 16, 100, and 1000 neurons.
