# Supplementary material for: Combining sampling and attractor dynamics in spiking models of head direction systems
Source: PLoS Comput Biol. 2026 Jul 30;22(7):e1014577. doi: 10.1371/journal.pcbi.1014577 (PMC13450861; doi:10.1371/journal.pcbi.1014577)
Supplement: Supplementary file 9 [file pcbi.1014577.s009.pdf]

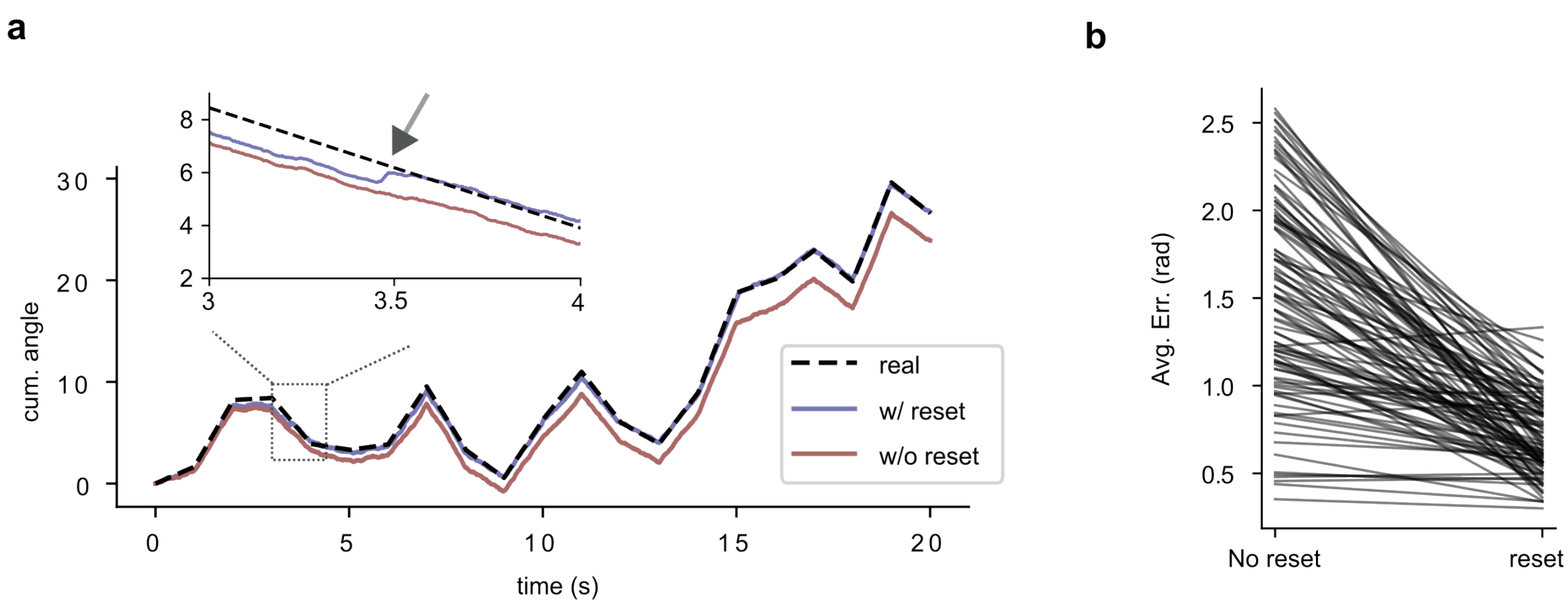

S8 Fig. Performance of a visual reset mechanism.
(a) Comparison of cumulative angle for real vs. HD with reset vs. HD without reset over 20 seconds behavior. Inset: zoom in to show the effect of the reset. Arrow represents a reset event. **(b)** Avg. error after 20 seconds behavior with vs. without reset mechanisms.
