# Supplementary material for: Combining sampling and attractor dynamics in spiking models of head direction systems
Source: PLoS Comput Biol. 2026 Jul 30;22(7):e1014577. doi: 10.1371/journal.pcbi.1014577 (PMC13450861; doi:10.1371/journal.pcbi.1014577)
Supplement: Supplementary file 10 [file pcbi.1014577.s010.pdf]

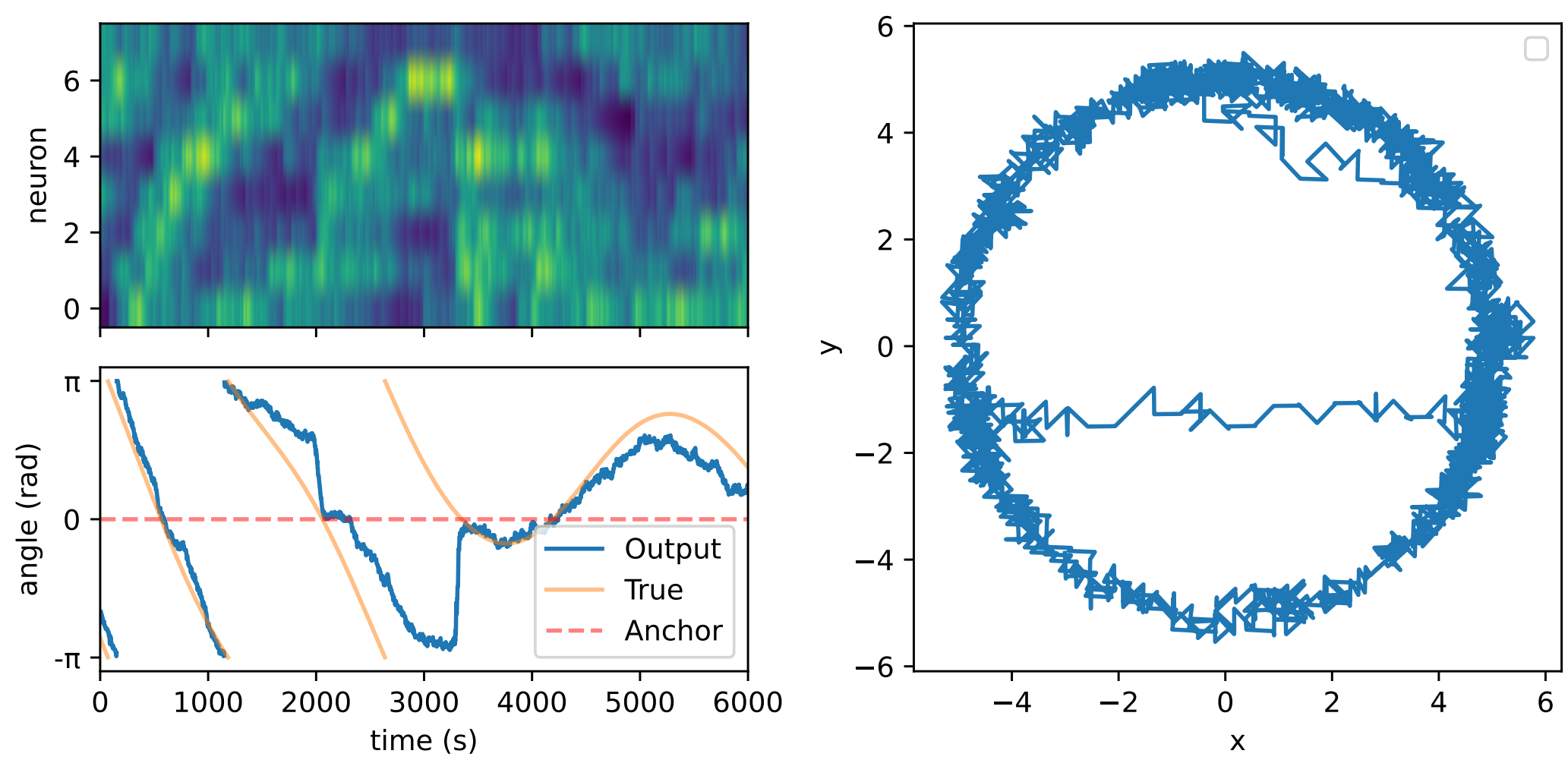

S9 Fig. Excursions from circular manifold. Due to the assumption of 2D angular representation and the sampling-based integration of angular velocity inputs, the network never fully "sticks" to the circle, but is simply attracted towards it, with increasing strength as the network output moves further away from a pre-established radius. During reset events, some large transient excursions away from the circular manifold become more evident. These observations are consistent with engagement of an additional latent dimension during cue-driven HD correction as described in recent experimental observations in mammals.
