# Supplementary material for: Combining sampling and attractor dynamics in spiking models of head direction systems
Source: PLoS Comput Biol. 2026 Jul 30;22(7):e1014577. doi: 10.1371/journal.pcbi.1014577 (PMC13450861; doi:10.1371/journal.pcbi.1014577)
Supplement: Supplementary file 11 [file pcbi.1014577.s011.pdf]

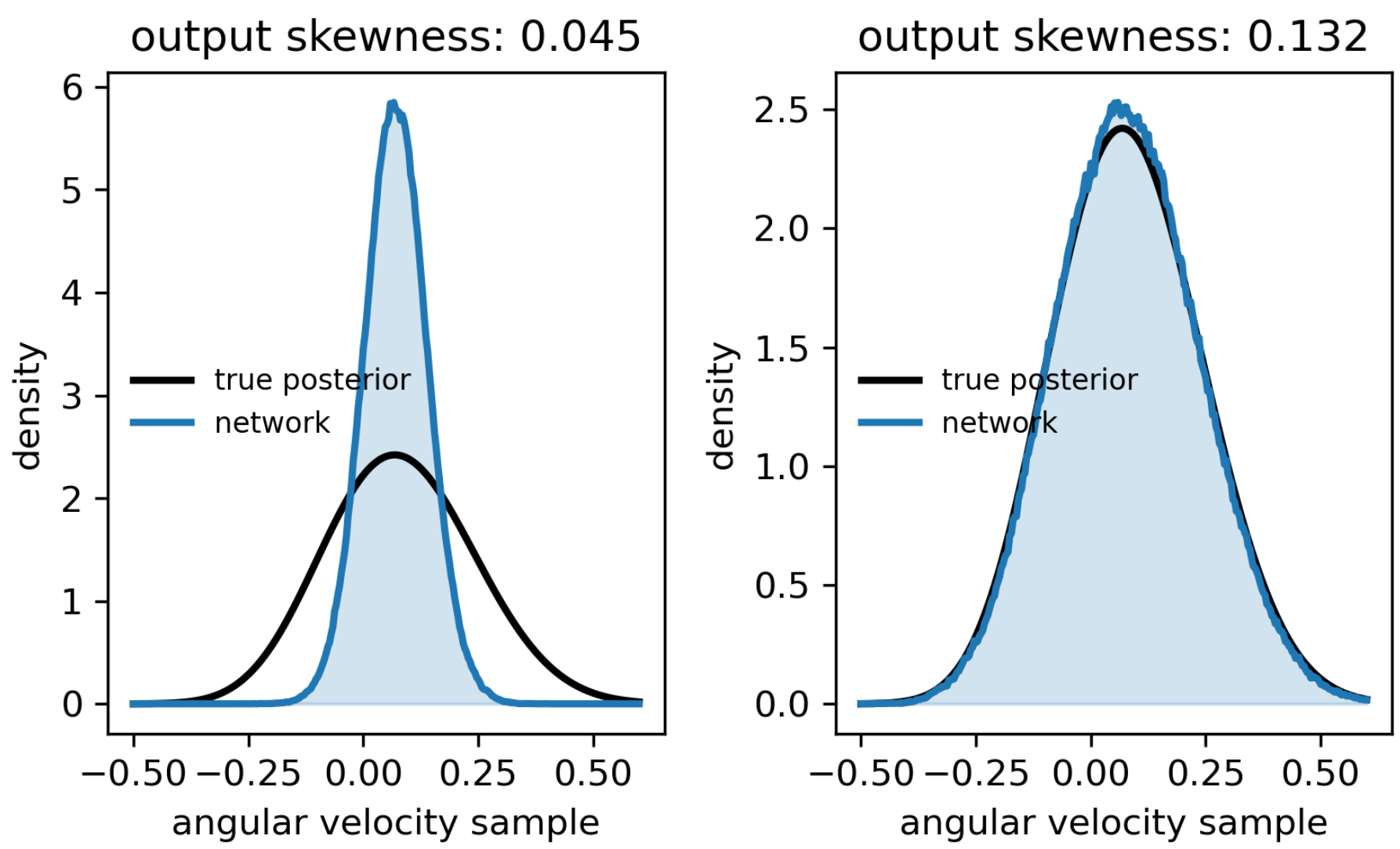

S10 Fig. Comparison of independent voltage noise (left) vs correlated voltage fluctuations (right). For each neuron, independent noise is scaled to match the same average per-neuron variance of correlated fluctuations.
