# Supplementary material for: Combining sampling and attractor dynamics in spiking models of head direction systems
Source: PLoS Comput Biol. 2026 Jul 30;22(7):e1014577. doi: 10.1371/journal.pcbi.1014577 (PMC13450861; doi:10.1371/journal.pcbi.1014577)
Supplement: Supplementary file 12 [file pcbi.1014577.s012.pdf]

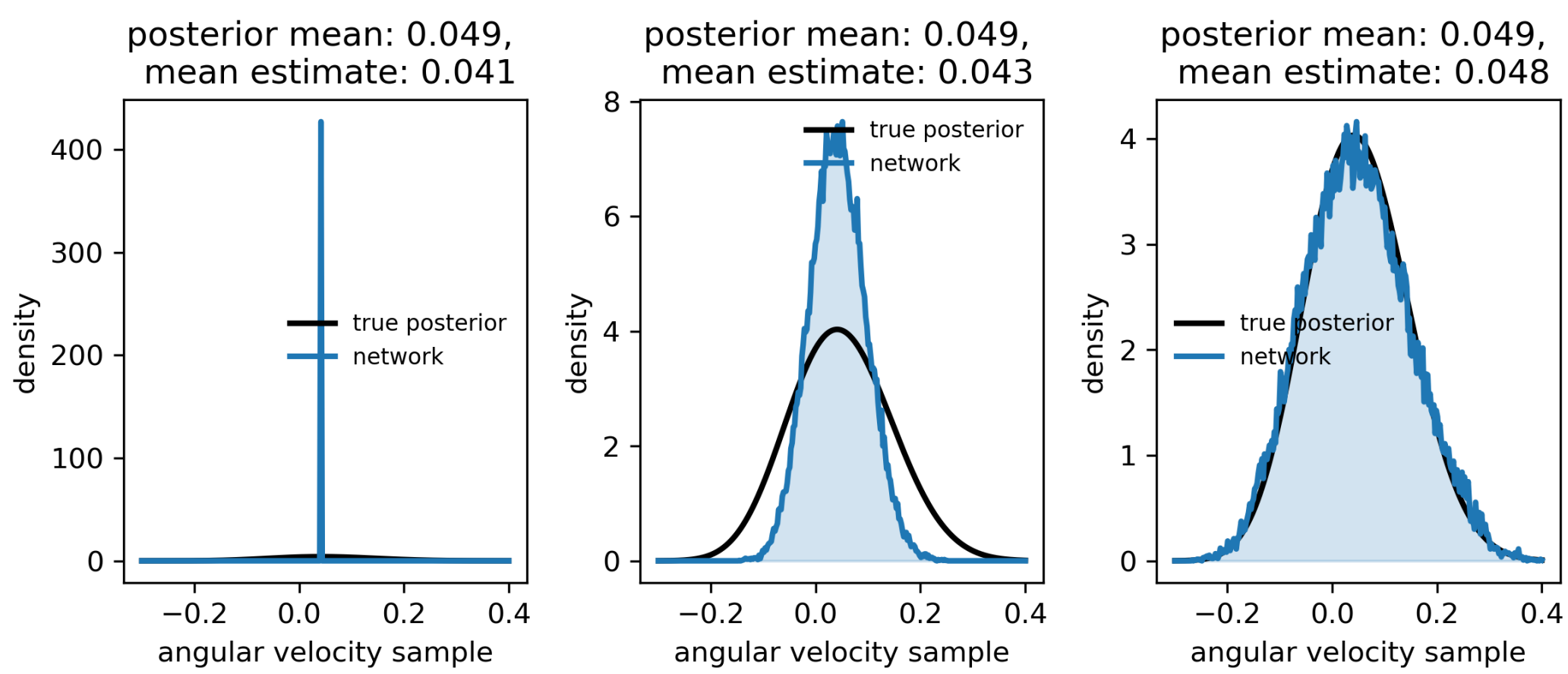

S11 Fig. Removing correlated voltage fluctuation biases network posterior mean estimate. From left to right, SCN implementing sampling-based inference of a constant angular velocity ω using zero, 0.5, and standard (unscaled) stochastic fluctuations.
