# Supplementary material for: Combining sampling and attractor dynamics in spiking models of head direction systems
Source: PLoS Comput Biol. 2026 Jul 30;22(7):e1014577. doi: 10.1371/journal.pcbi.1014577 (PMC13450861; doi:10.1371/journal.pcbi.1014577)
Supplement: Supplementary file 13 [file pcbi.1014577.s013.pdf]

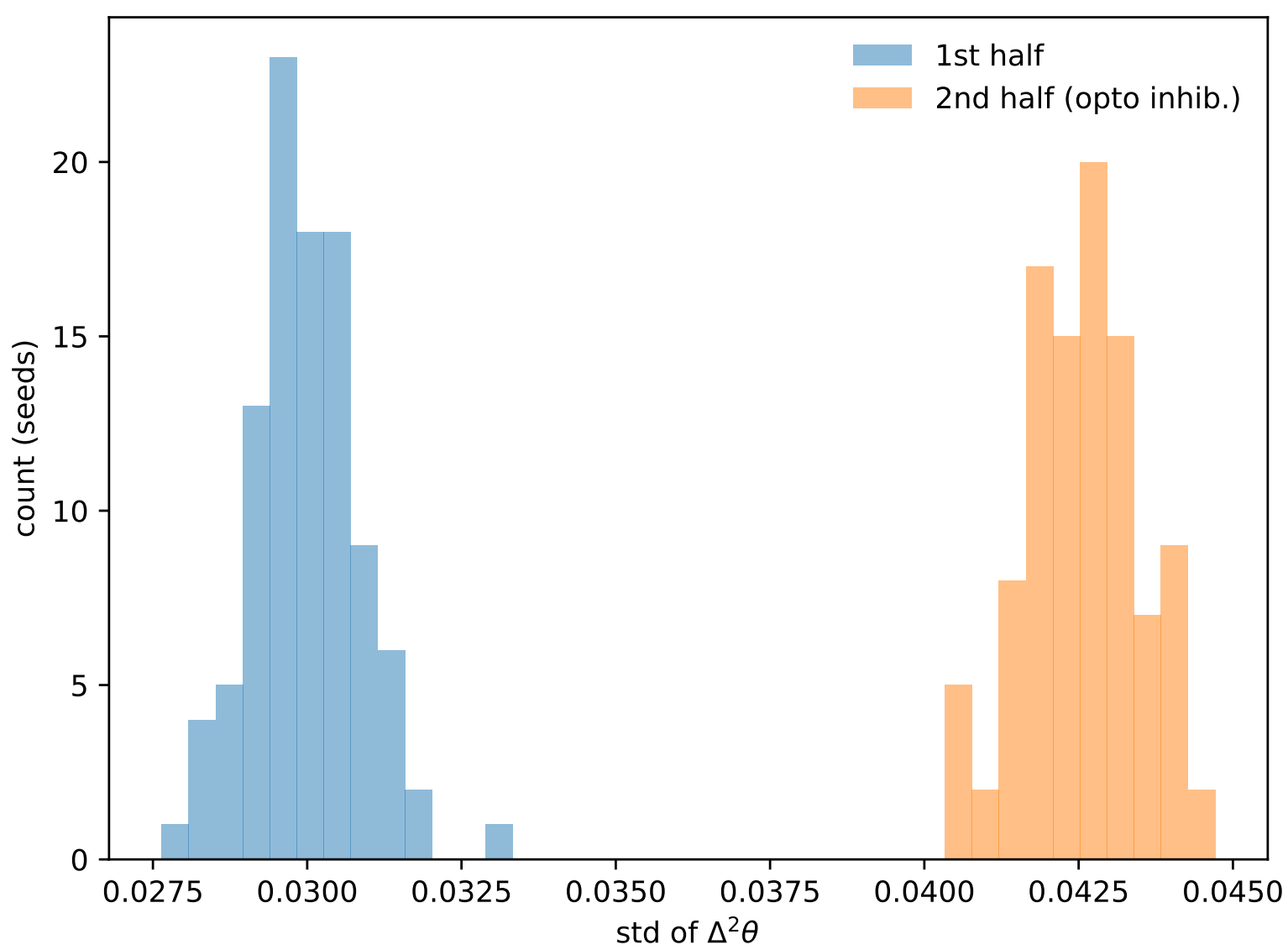

S12 Fig. Ensemble statistics for optogenetic inhibition experiment. For an ensemble of a 100 simulations, we scaled the neuronal gain of the encoding neurons midway $\beta \rightarrow 0.2\beta$, and consequently calculated the standard deviation of the second-order difference in bump readout for the first and second halves of the simulation.
