# Supplementary material for: Combining sampling and attractor dynamics in spiking models of head direction systems
Source: PLoS Comput Biol. 2026 Jul 30;22(7):e1014577. doi: 10.1371/journal.pcbi.1014577 (PMC13450861; doi:10.1371/journal.pcbi.1014577)
Supplement: Supplementary file 14 [file pcbi.1014577.s015.pdf]

Response to reviewers

We thank the reviewers for their thoughtful and constructive feedback. We have revised the manuscript to improve clarity, strengthen the connection to biology, and provide additional support for our claims. The major changes include:

- **Clarification of scope, contextualization, and biological interpretation.** We have revised the Introduction and Discussion to more clearly distinguish between the general theoretical framework and its application to the head-direction (HD) system, emphasizing that our goal is to provide a principled computational framework and generate testable predictions, rather than a complete/faithful biological model. Furthermore, we refined our positioning relative to prior work on sampling-based inference and attractor dynamics and incorporated relevant literature suggested by the reviewers. Finally, we now clarify how uncertainty, represented through bump fluctuations, could be used by downstream circuits as an implicit reliability signal (e.g., for cue integration or modulation of navigation computations).
- **Improved presentation of the model and methods.** We reorganized the Methods section, moved didactic SCN derivations to the Supplementary Material, and added a new explanatory box and key equations in the main text to clarify assumptions, network structure, and synaptic connectivity.
- **New analyses validating the sampling-based interpretation.** We added quantitative analyses comparing the network output to the true posterior, as well as ablation studies (e.g., removal or alteration of stochastic fluctuations), to demonstrate when and how the network correctly represents the posterior distribution as a function of parameters.

Overall, we believe these revisions greatly improved the manuscript. In the following, we include a detailed point-by-point answer to the reviewer's comments.

Best regards,
The authors

General notes on response

For clarification, we add a few notes on how we structured our response:

1. We provide a pdf file containing the diffs in our work, to clearly show alterations where applied.
2. A major part of the methods has been moved to the supplementary materials, which is not highlighted in the diff for clarity, since otherwise the whole sections would be highlighted; however, if any of the contents were altered, this would be shown by the diff.
3. Line numbers mentioned in responses below will refer to the line numbers in the resulting diff file.
4. Additional citations are not rendered

Point-by-point response to Reviewer 1

The work by Pjanovic and colleagues is a theoretical paper in which a spiking neural network model is developed to show how sampling-based inference and attractor-based computation may be used in head-direction circuits (across species). Four specific predictions generated by this theoretical model are clearly laid out, and contrasted with alternative models, such as Kalman-filter based optimal Bayesian updating of location and uncertainty encoding suggested by others in the field. The contrast between these theories speaks to a long-standing concern in the field, echoing debates from a decade ago on whether population codes encoded probabilities by expressing parameters of distributions or by representing samples from the distribution directly. The main contributions of the paper are to (1) extending the spike coding network to accommodate nonlinear dynamical systems and (2) developing a sampling-based theory of HD circuitry in which the attractor dynamics of the system operate as a prior. A concern is that these are two very distinct contributions, deserving dedicated treatment. The mathematical framework (nonlinear SCN + Langevin embedding) is a theoretical result that deserves to be presented as such, with clear statements of what is novel, what assumptions are required, and what the framework can and cannot do. As currently written, the framework is too compressed to evaluate rigorously. The HD circuit model is an application of that framework and should be evaluated on whether it genuinely exercises the machinery and makes specific predictions for the biology. The predictions laid out in this paper accomplish this partly (and are a strength of this theoretical paper). Overall, this paper has strong potential, but it is not currently written in a way to appeal to the broad audience of Plos-Computational Biology.

Strengths:

1. *Introduces a novel extension to the spike coding network (SCN) introduced a decade ago. The model here is crafted to use Langevin dynamics to sample an attractor landscape, in which energy determines the posterior distribution of head direction (- log P(samples—state) ∼ E(state)). A strength of the model is that it can represent non-Gaussian distributions, enabling much more flexible computation than Kalman filter approaches commonly used.*
2. *Clearly lays out predictions for their theoretical framework. The four predictions arise from the SCN dynamics and are as follows: 1) Shared voltage fluctuations: Neurons with similar tuning should have shared sub-threshold fluctuations, reflecting the coordinated sampling of the posterior across the population 2) Multi-timescale dynamics: a. Fast e/i balance on few millisecond timescale - sampling b. Slower timescale (10 ms) implementing attractor dynamics Predictions (1) and (2) follow directly from early work on SCN dynamics (Boerlin et al. 2013). 3) Bump dynamics on the attractor are more "jittery" during uncertainty, and slower when uncertainty is low. The bump itself does not flatten out, but time-averages will look flatter because of the movement. This is a novel prediction that distinguishes this theory from the Kalman-based theory, in which the bump height itself is related to uncertainty. 4) Asymmetry and skew in bump velocity distribution, arising from the Poisson firing of neurons, not from the attractor dynamics.*

Weaknesses:

1. *The paper may understate its novelty (or perhaps I misunderstood it): this framework is more general than the circular Kalman filter model, which is probably the closest to a clear alternative model I'm aware of in the literature. Specifically, the Langevin sampling is not constrained to quadratic energy terms (equivalently, Gaussian samples) – this is pointed out in the Methods but likely has implications in the results/predictions*

as well. Bayesian updates of non-Gaussian priors are fundamentally different from Gaussians: in systems with Gaussian priors, any perturbation produces a proportional shift in the estimate, whereas non-Gaussian priors can produce qualitatively different behavior such as robustness to large conflicts between internal estimates and sensory input. This has been demonstrated in other systems — for example, in songbirds, large perturbations of auditory feedback are rejected rather than integrated, a result that cannot be explained without non-Gaussian priors (refs — Sober, Nemenman); similarly, audio-visual recalibration in barn owls induced by prismatic displacement has a ceiling effect that Gaussian cue integration cannot account for.

Response: We thank the reviewer for pointing out the broader impacts of our model approach. This was indeed under-emphasized and not fully clear. To address the reviewer's comment, we have included a new Box 1 in the main text, explaining the implementation of non-Gaussian sampling in our network, strengthening the technical novelty and the general use of the network as a sampler (line 90-91); moreover, we added references to the literature suggested by the reviewer of non-Gaussian priors as a motivation (line 90-91).

2. *The treatment of visual input in the HD circuit as a pure reset — regardless of its conflict with the current estimate — implicitly assumes infinite reliability of the visual cue, which sits in conflict with the non-Gaussian generality that is the framework's main advantage over Kalman filter approaches. A more principled treatment of visual reset as reliability-weighted cue integration would both strengthen the paper's claims to generality and generate an additional experimentally testable prediction, since cue conflict experiments in HD systems are within experimental reach.*

   Response: We agree with the reviewer that assuming infinite reliability of visual inputs would defeat the point of the framework's generality. In the new version of the text, we better explained our reset mechanism in the results, which indeed is a reliability-weighted cue integration, in that the sampling happens over the sum of log posteriors (line 146-163); Notice how, in figure 3a, we clearly show that the anchor cue is encoded noisily, and the precision of the encoding determines the reliability of the cue (and how quickly the realignment is implemented). This process does not happen dynamically – the reliability of the input is assumed to be fixed; a full treatment of dynamically inferring and adjusting the reliability of the visual inputs goes beyond the scope of the current paper.

3. *The authors refer to the 'naïve Langevin dynamics' on line 292. Please be explicit about what makes the Langevin dynamics "naïve."*

   Response: In the previous version of the text, we referred to "naive" Langevin dynamics as the standard sampler that uses the pure gradient of log P, rather than employing projections or transformations to speed up sampling, as suggested in, e.g., Masset et al., 2022. We replaced it with "standard" (line 436).

4. *The statement that attractor networks and sampling-based inference haven't previously been integrated seems narrowly true, perhaps only so in the context of the HD circuit, but there are other systems in which attractors and sampling-based inference have been considered. Examples in sensory cortex (including work cited in the paper, from the Lengyel group) pointed to single attractor in sensory cortex. The computational novelty is that the attractor structure considered here is more complex and flexible.*

   Response: We agree that previous work used stochastic neural dynamics and probabilistic inference, including studies in sensory cortex where network variability has been interpreted as sampling from a posterior distribution. In some of these works, network dynamics can be viewed as evolving within an energy landscape. Our contribution explicitly embeds sampling-based inference within a continuous attractor system, where the attractor dynamics implement a specific prior and sampling operates over inputs that drive motion along this manifold. This distinction is relevant in the HD system, where continuous attractors play a central functional role. As the reviewer suggests, the strength of our framework lies in its top-down integration of sampling and attractor dynamics, which flexibly handles complex probabilistic computations and enables the representation of both estimates and input uncertainty within a single circuit. To avoid confusion and contextualize our contribution relative to previous work, we have reworked the introduction to better explain the difference between previous implementations and ours (line 43-51)

5. *The methods section is extremely difficult to follow. For instance, much of the derivation of the SNN framework appears to follow that of Boerlin, Machens and Deneve, but without the clarity of the original paper, which states up front that the goal is to construct a network simulates an arbitrary linear dynamical system, and the derives the SNN dynamics that accomplish this. Here, the logic feels inverted, making it hard to evaluate what is being assumed versus derived. I would suggest referencing the original work and highlighting where your derivation extends theirs.*

   Response: We agree that the Methods were quite heavy on pre-existing mathematical machinery, which we had initially included to provide a didactic build-up of our model. To clarify and highlight the main contributions, we have rearranged the text and methods section. To this end, we briefly introduced classic SCN in the main text, and focused on where our implementation differs from previous approaches (line 90-116). Moreover, we reordered the methods, moving all derivations except the main mechanisms for this model to the supplemental information, and in the methods we clearly defined the goal of implementing inference and HD integration in a spiking neural network.

6. *Relatedly, it is not obvious that the entirety of the complex machinery derived in the methods are necessary for the results of the paper. This was what I got from the methods. First, the Boerlin/Deneve SCN framework is introduced, in which membrane potentials track a prediction error and spikes are greedy corrections to that error, with connectivity derived from a coding objective. Second, this framework is extended to nonlinear dynamical systems by approximating F(z) in a polynomial basis, yielding the "multiplicative SCN" (mSCN). Third, Langevin sampling is embedded in the SCN voltage equation by substituting the gradient of an energy function U(z) (where $P(z) \propto exp(-U(z))$) for the dynamical term — this is the conceptual hinge of the paper. For quadratic U this reduces to the linear SCN; for non-quadratic U the polynomial/mSCN machinery handles the nonlinear gradient terms. Fourth, this framework is applied to a Poisson observation model, in which a latent variable z modulates input firing rates, and the resulting energy function is plugged into the membrane potential equation; several rate kernels (exponential, linear, logistic) and noise models are worked through in turn. The HD circuit derivation then introduces angular coordinates, moves to 2D Cartesian to handle the wrapping problem, imposes a soft circular constraint, and derives the SCN dynamics for this system — but apparently using the standard (eqn. 31) rather than multiplicative SCN update equations. The mSCN development (parts 2 through 4) appears not to be used by the HD circuit application, which raises the question of whether this material belongs in the methods of this paper at all, or whether it represents a separate technical contribution that would be better suited to its own treatment elsewhere.*

   Response: As mentioned above, we agree that the previous version of the manuscript had a methods section that was not focused on the main results of the paper, but rather introduced SCN in a didactic manner. In the new text, we have rearranged the methods to be more concrete about the goals of the paper, and moved the more "didactic" part of the derivations to supplementary material. Nonetheless, we find that the notion of multiplicative interactions (through the mSCN framework) is necessary, since the prior we use for HD integration ("soft circular constraint") requires polynomial computations, making it a central component of the network.

7. *Minor: Statements like "mirror recent experimental observations during head-direction realignment in mammals" need a reference (Fig. 3 caption). Minor inconsistency in notation: Equations 7 through 9 use both boldface and a subscript for variables v and T, which I understood to be Nx1 vectors. Either v or v_i, but not both.*

   Response: We removed the statement to avoid complications in the Figure caption, and we fixed the notation in the mentioned equations (line 400-404).

Reviewer 2

This is an interesting paper that examines how attractor dynamics and uncertainty representations can be multiplexed in a single spiking neural network, with a specific focus on the head direction system. It is clearly written and relevant to understanding probabilistic computations in attractor systems. The idea that observed variability may be not "just noise" that degrades an attractor, but functional, is also very interesting from a theoretical perspective. I appreciate the testable experimental predictions.

Some recommendations and questions:

1. *It seems that the main claim of the paper is that the spiking variability represents a posterior, i.e. is performing Bayesian inference over the heading angle. However, the main figures did not show this very clearly. It would be very helpful to see an overlay of the true posterior and that represented by the network, and perhaps a small analysis of parameter regimes in which the network representation is more or less accurate.*

   Response: We agree with the reviewer that the main idea, i.e., that network output variability represents input uncertainty, did not come through in the main figure. In the new version, we have included a schematic showing how input uncertainty is implicitly encoded in the network output in the form of larger or smaller bump movement (fig. 1e) Moreover, we have added figures explicitly showing an overlay of the true posterior vs. network output (fig. 2e), and an analysis of attractor strength vs JS divergence of true vs simulated posterior (supp. fig. 6e).

2. *The relationship between the network connectivity and its function are not entirely clear. It is mentioned that an established procedure was used to embed specific Langevin dynamics aligned to the ring manifold into the network, but it would be helpful to describe this in more detail in the main text. Additionally, does the connectivity end up with a ring-like topology, as in e.g. the fly HD system? Any additional analysis or intuition behind how the resulting connectivity patterns support the functions claimed in the rest of the manuscript would be very appreciated.*

   Response: To clarify the relationship between network connectivity and function, we have improved the explanation of the network structure and dynamics, including the voltage formulas and breakdown of the connectivity (line 103-116). The connectivity matrices for fast and slow synapses are shown in Fig 4b; we added the corresponding symbols to the caption for clarity. We confirm that the slow linear synapses, which implement the inference and integration, do settle on a ring-like topology - we now explicitly say so in the main text (line 186). Even though there are other slow interactions (e.g., nonlinear attractor), nearest neighbor excitation can also be seen from simple correlation matrices over 100ms (Fig. 4b).

3. *If I understand correctly, this particular spiking network follows from the line of balanced spiking networks originating with Boerlin, Machens, and Deneve 2013. These networks are certainly quite interesting, but given that they are a bit non-traditional, a short overview of how they work in the main text would be quite useful.*

   Response: We have added an introduction to SCN in the main text (Box 1), explaining the fundamental ideas behind the network architecture, and how we extended upon them.

4. *In general I think it would be very helpful to include the key equations in the main text. For example, the neuron and synaptic dynamics, network connectivity, where different noise sources enter, and how different features of inputs, activity, etc relate to the Bayesian quantities involved.*

   Response: We have improved the presentation of the network and included the key equations in the main text so that the reader can easily understand the relationship between the Bayesian update and the network equations, connectivity, and structure (line 91-116).

5. *In the absence of visual cues, is there a trade-off between the uncertainty in the representation and the stability of the attractor? Intuitively it seems larger uncertainty would correspond to more noise and in turn more drift of the bump, whereas if one dialed down this noise one could perhaps attain a more stable estimate but at the cost of a poorer encoding of uncertainty.*

   Response: Uncertainty, in our work, is given by the encoding of the angular velocity ω by a population of noisy Poisson neurons σ. Therefore, the uncertainty is strictly related to the posterior $P(\omega|\sigma)$, which is explored by Langevin sampling and integrated. The reviewer is right in thinking that, in the absence of visual cues, the uncertainty in the representation of angular velocity leads to more drift of the bump, as is shown in Fig. 2d. On the other hand, the reviewer seems to suggest that the "noise" term in Langevin sampling (what we call stochastic fluctuations) might be dialed down to reduce drift at the cost of not encoding uncertainty. This intuition, although enticing, is unfortunately incorrect: removing the stochastic fluctuations breaks detailed balance and changes the invariant object of the dynamics from a stochastic exploration of the

posterior to a deterministic attractor, which leads to less "wiggling" of the bump but also to representing the wrong quantity in general (i.e., integrating that quantity does not converge to the posterior mean and would lead to wrong estimates). We added Supp. Fig. 11 to showcase the necessity of this term.

6. *I also didn't fully understand whether one should think of the attractor and the uncertainty representation as "co-existing" vs "working together" somehow. A line or two explaining some intuition about this would help.*

   Response: This is an interesting point. We believe that the two mechanisms co-exist in the network, as it is (theoretically) possible to remove one or the other. On the other side, we show in supp. fig. 6, removing the attractor leads the network to random wandering in 2D, ruining the angular estimation. In lines 126-132 of the new text, we now explicitly talk about this in the context of supp. fig. 6e.

7. *Given that this is a spiking network, a short discussion of the relationship of the spiking statistics in the network model (e.g. firing rates) vs real spiking statistics in HD systems would be nice (if I am not mistaken, the EPG neurons spike in the fly. . . ?).*

   The units in our network spike in response to the network estimate being outside a so-called "bounding box" (Calaim et al., 2022). The size of this box is arbitrary, and has profound effects on the spiking statistics of the network, as does the number of neurons in the network. Therefore, we decided not to report spiking statistics in the paper, as we believe it depends on choices that do not depend on the logic of the computation implemented.

8. *Some other literature in which spiking variability does not just simply degrade attractors but plays some role in supporting them, that could be worth citing: Mongillo, Hansel, van Vreeswijk PRL 2012; and Pang PLoS CB 2025.*

   Response: We thank the reviewer for these suggestions, which clearly show how variability can support attractor-like or nonlinear dynamics, strengthening the point that noise can have a constructive role in network computation (line 278-283).

Reviewer 3

This is an interesting paper that brings together the neurocomputational principles of sampling-based probabilistic inference and attractor dynamics, as far as I am aware, in a novel way. It applies this to the neural task of tracking head direction (HD) from uncertain sensory cues (angular velocity and fixed landmark inputs).

1. *This is useful (as a 'case study') for illustrating the properties of the unified system, but somewhat less convincing as reinterpretation of HD circuits in biology, as it was not always clear what properties of these circuits it could newly account for, compared to alternative/existing models. Perhaps that is not the main claim the paper intends to make, in which case it could perhaps use some revision to avoid this misinterpretation of the core aim. More explicitly, it seems self-evident that "precision of HD representations...depends on the reliability of sensory cues" (line 10) but this does not seem sufficient alone to motivate modelling HD as an "uncertainty aware" circuit. There is no discussion in the paper of how this might augment downstream processing of HD information, for example, or why sampling-based inference "might be necessary" (line 43) or have some functional role in navigation. It is reasonable observation that attractor networks (including models of HD) have mostly treated noise as perturbation, whereas it could carry useful information; and legitimate to explore how this could be the case. However, the work presented is highly speculative (e.g. at no point based on anatomical data), and the results not fully convincing about the advantages of this specific approach to explaining HD circuit function.*

   Response: We agree that our primary goal is not to provide a biologically detailed implementation of HD circuits, but rather to propose a computational framework to generate testable hypotheses about the computational roles of noise and uncertainty in the network. In this context, the HD system is a well-characterized and experimentally accessible case study in which the role of stochasticity and/or the propagation of uncertainty to downstream areas remains debated. We have revised the manuscript to make this distinction more explicit in the introduction (line 57-63). In particular, we emphasize that our contribution is to provide a computational framework, derived from first principles, that can generate testable predictions. We hope

that this will avoid misunderstanding the model as a complete biological account, while still highlighting its potential relevance for understanding HD computational principles.

The reviewer is correct that the fact that HD precision depends on sensory reliability is not sufficient to guarantee an uncertainty-aware representation. This work is inspired by previous literature showing that uncertainty is represented in different systems and brain areas. Therefore, we explore this hypothesis and generate testable predictions to answer the question of whether the HD system does, indeed, represent stimulus uncertainty through sampling-based methods. The reviewer correctly pointed out that this work is exploratory, which we have highlighted more explicitly in lines 57-63.

We also agree that in the previous version of the manuscript, we did not offer enough reasons why a notion of uncertainty might be beneficial for downstream areas and the organism in general. We have expanded the manuscript to clarify the potential functional role of uncertainty in this system (line 126-137). Specifically, the sampled posterior is over angular velocity, and the fast fluctuations of the HD bump reflect the reliability of the self-motion signal. As we show in Supp. Fig. 4, the temporal statistics of bump motion encode the uncertainty (and higher order moments) of the sensory stimuli. A downstream circuit could use the short-timescale variability of the HD representation as an implicit reliability signal. For example, increased bump jitter could indicate that path integration is unreliable, favoring stronger weighting of external cues during reorientation, or reducing the gain of downstream computations that depend on heading estimates. We have added this interpretation to the Discussion (line 237-244).

2. *For example, line 96 onwards summarises how the model produces "intuitive and biologically relevant behavior, as well as several experimentally observed phenomena": a bump of activity, that tracks head direction well for reliable velocity inputs, with decreasing precision and accumulating error for noisy inputs, etc. I don't see how any of these are novel to this model, compared to previous models. Is there anything in these results that allows us to distinguish the observed phenomena from "noise superimposed on a deterministic attractor" (line 106)? The same caveat applies to the reset results, i.e., these also seem comparable to previous models.*

   Response: The results that we present in figure 2, such as a bump that tracks HD with higher/lower precision for higher/lower input quality, are baseline qualitative HD tracking behaviors that we expected our network to reproduce. In the results, we now explicitly state this and have removed the potentially misleading sentence "intuitive and biologically relevant" (line 119). These baseline results are not necessarily unique to our model; nonetheless, as we argue in the following answers, we have now included supplementary analyses that allow us to distinguish between structured and unstructured noise (Supp. Fig. 10 and 11; see responses below).

3. *Another limitation is that the consequences of the particular assumptions made to set up the network (and relate it to biological features) were not always sufficiently explored to help the reader understand which aspects were actually crucial to its function. For example, I understand it to be theoretically motivated, by the choice of sampling approach, that "the sampling process requires neurons to share structured sources of stochastic fluctuation" in a specific way, but is it actually necessary for the circuit to function for the HD task? And if this was not observed (e.g. in the fly) to be implemented in correlated voltage fluctuations (their prediction 1, line 139 on) then could it still be supposed to be implemented in some other form? Is the use of Langevin sampling critical?*

   Response: Building on the last question, we believe Langevin sampling is an effective and theoretically tractable dynamics-based sampling method, but sampling can be implemented in a number of ways - to that end, we have elaborated on previous approaches (line 46-51). That being said, the advantage of using a theoretically tractable method is that the resulting network structure and dynamics can be fully dissected and analyzed in different ways. In the new text, we improved the explanation of the network derivation and structure, making sure to explain the necessity of every single component (line 103-116). Moreover, we have added a supplementary to show that:

   (a) lowering the strength of the attractor, although it improved the representation of the posterior, leads to stronger deviations from the circle and quick deterioration of HD representation (Supp. Fig. 6e)

   (b) removing correlated noise and substituting it with uncorrelated noise, does not allow the network to explore the posterior over angular velocity (Supp. Fig. 10), and can lead to biased mean angular velocity estimates (Supp. Fig. 11).

4. *Despite the above issues, the paper does make significant contributions in developing, in a principled fashion, a spiking network that should maintain the best estimate of dynamic variable, assuming Poisson encoding and implementing Langevin sampling, constrained by a prior towards a (ring) attractor. A significant strength of the paper is that the methods work this out analytically for a variety of cases, e.g. different firing rate kernels, different noise models, linear or non-linear intrinsic dynamics, etc. As mentioned at the start of my review, this seems a very interesting and novel approach, with potential for wide application, even if its applicability to the HD system is not fully convincing.*

   Response: We thank the reviewer for the encouraging words. We hope that the new additions to the paper will highlight the strengths of the paper while avoiding confusion as to what we can and cannot do with our framework.

5. *Some additional comments:*
   - Fig 1 doesn't seem to quite connect together the various illustrated ideas because it does not show (in d) how the 'bump' behaviour actually reflects/encodes the uncertainty of the inferred distribution (through rapid fluctuations)

   Response: We have improved the schematic of Figure 1 to better convey the message that bump fluctuations encode input uncertainty (Fig. 1d,e).

   - Figure 2, the caption (b) seems to be the only place in the main text that explains how the circular prior is set up. Overall, I think the main text explanation of the methods needs to be expanded. Around line 83 it becomes confusing, the paragraph seems to start concerning sampling only, but then describes the whole dynamics including the circular manifold. Are the slow interactions implementing both the following the slope of the log distribution and the circle constraint?

   Response: We have reworked the results section to improve the explanation of the methods. In particular, we have separated the general implementation of sampling in spike coding networks (Box 1), and focused on the integration of inferred angular velocity samples in the results (line 91-105). This should help clarify which interactions implement which computation – in particular, the slow interactions that implement both the slope of $\log P$ and the circular attractor.

   - Fig2d, this seems to give the impression that the circuit has encoded uncertainty in the deviation from the true angle, but of course this is not known to the circuit (it does not know the true angle). But is it intended that there is some mechanism by which the neural system can extract and use the uncertainty as represented in the rapid fluctuations (2e)? This relates to prediction 3, around line 192, suggesting "motion of the bump reflects variability" not just the mean of the input. It was not entirely clear to me what observation would be expected under the alternative, e.g., that (at high resolution) it would be seen that the quality of the bump degrades, vs. the bump shape being maintained but fluctuating more in position. Or if the model output "reduces to integrating the posterior mean" how would the difference be detected?

   Response: We agree that the circuit does not have access to the true angle, and therefore cannot represent uncertainty as a deviation from ground truth. The quantity shown in Fig. 2d is used purely as an external evaluation metric to quantify performance, not as a quantity encoded by the network. We have modified the figure caption to explain this better and avoid suggesting that the network has access to the true angle (see caption for fig. 2d).

   As the reviewer pointed out, input uncertainty is not represented as an explicit variable, but is encoded implicitly in the temporal statistics of the bump motion (e.g., variability of its position). We have included a reference to the supplementary figure 5 where we show how the samples can be used by downstream areas to estimate second and higher-order moments of the posterior. Moreover, as mentioned above, we have now included more hypotheses on how downstream areas might use this type of uncertainty in the discussion (line 237-244).

   Finally, the bump shape in our network is mostly determined by the choice of the neurons' weights $\mathbf{D}$ and the voltage decay λ. Therefore, the millisecond-timescale bump shape does not change as a function of input quality, but averaging over hundreds of ms (as in calcium imaging) can lead to an "artificial widening" when input quality is low. Similarly, in our model, if we excluded the correlated voltage fluctuations and included noise as uncorrelated perturbations of each neuron's voltage, we still expect the same ms-timescale bump

shape. Nonetheless, even though in the limit of fast neural dynamics the bump trajectory corresponds to integrating the posterior mean, the sampling formulation predicts that the statistics of movement reflect properties of the posterior (such as skewness or other moments) that are not captured by classical models in which noise is treated as a perturbation.

- Fig 3c I am not sure what I should examine in this figure to observe "excursions away from the circular manifold".

Response: We have improved the text (see caption fig. 3b-c) and added a supp. fig (Supp. Fig. 9) to explicitly show this effect during reset, where the network deviates quite strongly from the circular manifold due to the reset signal.

- Prediction 4, asymmetry and skew seems a counterintuitive prediction, but could be better explained, and is it not predicted in models that do not sampling based inference (4D)? In general, it feels like the paper could be improved if the results could be compared to the most similar possible model that does not preserve the variance.

Response: We agree that this type of higher-moment statistic could feel counterintuitive, and we have expanded the explanation in the result section (line 207-219). As the reviewer suggests, when the model does not receive the right type of correlated fluctuations, it won't show the same amount of skew that characterizes Poisson posteriors. Comparing our results to a network with the same per-neuron average variance but independent across neurons, the posterior will look "skinnier" and very little skew, as we now show in Supp. Fig. 11.
