# Supplementary material for: Combining sampling and attractor dynamics in spiking models of head direction systems
Source: PLoS Comput Biol. 2026 Jul 30;22(7):e1014577. doi: 10.1371/journal.pcbi.1014577 (PMC13450861; doi:10.1371/journal.pcbi.1014577)
Supplement: Peer review history

# Peer review: Decision Letter 0

**DOI:** 10.1371/journal.pcbi.1014577.r001

**Authors:** Daniel Bush, William Redman

1 Apr 2026

PCOMPBIOL-D-26-00420

Combining sampling and attractor dynamics in spiking models of head direction systems

PLOS Computational Biology

Dear Dr. Keemink,

Thank you for submitting your manuscript to PLOS Computational Biology. After careful consideration, we feel that it has merit but does not fully meet PLOS Computational Biology's publication criteria as it currently stands. Therefore, we invite you to submit a revised version of the manuscript that addresses the points raised during the review process.

Please submit your revised manuscript by Jun 01 2026 11:59PM. If you will need more time than this to complete your revisions, please reply to this message or contact the journal office at ploscompbiol@plos.org. When you're ready to submit your revision, log on to https://www.editorialmanager.com/pcompbiol/ and select the 'Submissions Needing Revision' folder to locate your manuscript file.

Please include the following items when submitting your revised manuscript:

* A letter that responds to each point raised by the editor and reviewer(s). You should upload this letter as a separate file labeled 'Response to Reviewers'. This file does not need to include responses to formatting updates and technical items listed in the 'Journal Requirements' section below.

* A marked-up copy of your manuscript that highlights changes made to the original version. You should upload this as a separate file labeled 'Revised Manuscript with Track Changes'.

* An unmarked version of your revised paper without tracked changes. You should upload this as a separate file labeled 'Manuscript'.

If you would like to make changes to your financial disclosure, competing interests statement, or data availability statement, please make these updates within the submission form at the time of resubmission. Guidelines for resubmitting your figure files are available below the reviewer comments at the end of this letter

We look forward to receiving your revised manuscript.

Kind regards,

William Redman

Academic Editor

PLOS Computational Biology

Daniel Bush

Section Editor

PLOS Computational Biology

**Additional Editor Comments:**

Dear Authors,

Thank you for your submission to PLOS Computational Biology. All 3 reviewers remarked on the novelty and strengths of the paper, and it is clearly well suited for PLOS Computational Biology. That being said, the reviewers did identify areas that could have greater clarity, particularly around the methods and the biological plausibility. These are certainly addressable (I would recommend medium revision if that was an option), but I do think they need to be addressed before acceptance.

Best,

Will Redman

**Journal Requirements:**

If the reviewer comments include a recommendation to cite specific previously published works, please review and evaluate these publications to determine whether they are relevant and should be cited. There is no requirement to cite these works unless the editor has indicated otherwise.

1) We ask that a manuscript source file is provided at Revision. Please upload your manuscript file as a .doc, .docx, .rtf or .tex. If you are providing a .tex file, please upload it under the item type u2018LaTeX Source Fileu2019 and leave your .pdf version as the item type u2018Manuscriptu2019.

2) Please provide an Author Summary. This should appear in your manuscript between the Abstract (if applicable) and the Introduction, and should be 150-200 words long. The aim should be to make your findings accessible to a wide audience that includes both scientists and non-scientists. Sample summaries can be found on our website under Submission Guidelines:

https://journals.plos.org/ploscompbiol/s/submission-guidelines#loc-parts-of-a-submission

3) Please upload all main figures as separate Figure files in .tif or .eps format. For more information about how to convert and format your figure files please see our guidelines:

https://journals.plos.org/ploscompbiol/s/figures

4) We notice that your supplementary Figures are included in the manuscript file. Please remove them and upload them with the file type 'Supporting Information'. Please ensure that each Supporting Information file has a legend listed in the manuscript after the references list.

5) Please amend your detailed Financial Disclosure statement. This is published with the article. It must therefore be completed in full sentences and contain the exact wording you wish to be published.

1) State what role the funders took in the study. If the funders had no role in your study, please state: "The funders had no role in study design, data collection and analysis, decision to publish, or preparation of the manuscript."

2) If any authors received a salary from any of your funders, please state which authors and which funders..

If you did not receive any funding for this study, please simply state: u201cThe authors received no specific funding for this work.u201d

6) Some material included in your submission may be copyrighted. According to PLOS's copyright policy, authors who use figures or other material (e.g., graphics, clipart, maps) from another author or copyright holder must demonstrate or obtain permission to publish this material under the Creative Commons Attribution 4.0 International (CC BY 4.0) License used by PLOS journals. Please closely review the details of PLOS's copyright requirements here: PLOS Licenses and Copyright. If you need to request permissions from a copyright holder, you may use the PLOS Content Copyright Permission Form Please respond directly to this email and provide any known details concerning your material's license terms and permissions required for reuse, even if you have not yet obtained copyright permissions or are unsure of your material's copyright compatibility. Once you have responded and addressed all other outstanding technical requirements, you may resubmit your manuscript within Editorial Manager. Potential Copyright Issues:

1. Figures 1C, 1D, 2A, 2B, Prediction 1A, S5A, and S6C. Please confirm whether you drew the images / clip-art within the figure panels by hand. If you did not draw the images, please provide (a) a link to the source of the images or icons and their license / terms of use; or (b) written permission from the copyright holder to publish the images or icons under our CC BY 4.0 license. Alternatively, you may replace the images with open source alternatives. See these open source resources you may use to replace images / clip-art: - https://commons.wikimedia.org - https://openclipart.org/.

1. We note that 1A, Prediction 1A are created through BioRender. Please confirm that you hold a Premium account and provide a pdf copy of the CC BY 4.0 Licence as provided by BioRender. For instructions on how to generate a CC BY 4.0 license for your figure, please see the guidelines here: https://help.biorender.com/hc/en-gb/articles/21282341238045-Publishing-in-open-access-resources

Please ensure the figure legend contains appropriate attribution text & a link to biorender.com

If you are using the free assets from BioRender, we are unable to publish these images as they are licenced under a stricter licence than CC BY 4.0. In this case we ask you to remove the BioRender images and replace them with open source alternatives.

See these open source resources you may use to replace images / clip-art:

- https://bioart.niaid.nih.gov/

- https://bioicons.com/

- https://healthicons.org/

- https://scidraw.io/

- https://reactome.org/icon-lib

- https://www.phylopic.org/images

- https://journals.plos.org/plosbiology/article?id=10.1371/journal.pbio.3002395

**Reviewers' comments:**

Reviewer's Responses to Questions

**Comments to the Authors:**

**Please note here if the review is uploaded as an attachment.**

Reviewer #1: The work by Pjanovic and colleagues is a theoretical paper in which a spiking neural network model is developed to show how sampling-based inference and attractor-based computation may be used in head-direction circuits (across species). Four specific predictions generated by this theoretical model are clearly laid out, and contrasted with alternative models, such as Kalman-filter based optimal Bayesian updating of location and uncertainty encoding suggested by others in the field. The contrast between these theories speaks to a long-standing concern in the field, echoing debates from a decade ago on whether population codes encoded probabilities by expressing parameters of distributions or by representing samples from the distribution directly.

The main contributions of the paper are to (1) extending the spike coding network to accommodate nonlinear dynamical systems and (2) developing a sampling-based theory of HD circuitry in which the attractor dynamics of the system operate as a prior. A concern is that these are two very distinct contributions, deserving dedicated treatment. The mathematical framework (nonlinear SCN + Langevin embedding) is a theoretical result that deserves to be presented as such, with clear statements of what is novel, what assumptions are required, and what the framework can and cannot do. As currently written, the framework is too compressed to evaluate rigorously. The HD circuit model is an application of that framework and should be evaluated on whether it genuinely exercises the machinery and makes specific predictions for the biology. The predictions laid out in this paper accomplish this partly (and are a strength of this theoretical paper).

Overall, this paper has strong potential, but it is not currently written in a way to appeal to the broad audience of Plos-Computational Biology.

Strengths:

1. Introduces a novel extension to the spike coding network (SCN) introduced a decade ago. The model here is crafted to use Langevin dynamics to sample an attractor landscape, in which energy determines the posterior distribution of head direction ( - log P(samples|state) ~ E(state)). A strength of the model is that it can represent non-Gaussian distributions, enabling much more flexible computation than Kalman filter approaches commonly used.

2. Clearly lays out predictions for their theoretical framework. The four predictions arise from the SCN dynamics and are as follows:

1) Shared voltage fluctuations: Neurons with similar tuning should have shared sub-threshold fluctuations, reflecting the coordinated sampling of the posterior across the population

2) Multi-timescale dynamics:

a. Fast e/i balance on few millisecond timescale - sampling

b. Slower timescale (~10 ms) implementing attractor dynamics

Predictions (1) and (2) follow directly from early work on SCN dynamics (Boerlin et al. 2013).

3) Bump dynamics on the attractor are more “jittery” during uncertainty, and slower when uncertainty is low. The bump itself does not flatten out, but time-averages will look flatter because of the movement. This is a novel prediction that distinguishes this theory from the Kalman-based theory, in which the bump height itself is related to uncertainty.

4) Asymmetry and skew in bump velocity distribution, arising from the Poisson firing of neurons, not from the attractor dynamics.

Weaknesses:

1) The paper may understate its novelty (or perhaps I misunderstood it): this framework is more general than the circular Kalman filter model, which is probably the closest to a clear alternative model I'm aware of in the literature. Specifically, the Langevin sampling is not constrained to quadratic energy terms (equivalently, Gaussian samples) – this is pointed out in the Methods but likely has implications in the results/predictions as well. Bayesian updates of non-Gaussian priors are fundamentally different from Gaussians: in systems with Gaussian priors, any perturbation produces a proportional shift in the estimate, whereas non-Gaussian priors can produce qualitatively different behavior such as robustness to large conflicts between internal estimates and sensory input. This has been demonstrated in other systems — for example, in songbirds, large perturbations of auditory feedback are rejected rather than integrated, a result that cannot be explained without non-Gaussian priors (refs — Sober, Nemenman); similarly, audio-visual recalibration in barn owls induced by prismatic displacement has a ceiling effect that Gaussian cue integration cannot account for. The treatment of visual input in the HD circuit as a pure reset — regardless of its conflict with the current estimate — implicitly assumes infinite reliability of the visual cue, which sits in conflict with the non-Gaussian generality that is the framework's main advantage over Kalman filter approaches. A more principled treatment of visual reset as reliability-weighted cue integration would both strengthen the paper's claims to generality and generate an additional experimentally testable prediction, since cue conflict experiments in HD systems are within experimental reach.

2) The authors refer to the ‘naïve Langevin dynamics’ on line 292. Please be explicit about what makes the Langevin dynamics “naïve.”

3) The statement that attractor networks and sampling-based inference haven’t previously been integrated seems narrowly true, perhaps only so in the context of the HD circuit, but there are other systems in which attractors and sampling-based inference have been considered. Examples in sensory cortex (including work cited in the paper, from the Lengyel group) pointed to single attractor in sensory cortex. The computational novelty is that the attractor structure considered here is more complex and flexible.

4) The methods section is extremely difficult to follow. For instance, much of the derivation of the SNN framework appears to follow that of Boerlin, Machens and Deneve, but without the clarity of the original paper, which states up front that the goal is to construct a network simulates an arbitrary linear dynamical system, and the derives the SNN dynamics that accomplish this. Here, the logic feels inverted, making it hard to evaluate what is being assumed versus derived. I would suggest referencing the original work and highlighting where your derivation extends theirs.

5) Relatedly, it is not obvious that the entirety of the complex machinery derived in the methods are necessary for the results of the paper. This was what I got from the methods. First, the Boerlin/Deneve SCN framework is introduced, in which membrane potentials track a prediction error and spikes are greedy corrections to that error, with connectivity derived from a coding objective. Second, this framework is extended to nonlinear dynamical systems by approximating F(z) in a polynomial basis, yielding the "multiplicative SCN" (mSCN). Third, Langevin sampling is embedded in the SCN voltage equation by substituting the gradient of an energy function U(z) (where P(z) ∝ exp(-U(z))) for the dynamical term — this is the conceptual hinge of the paper. For quadratic U this reduces to the linear SCN; for non-quadratic U the polynomial/mSCN machinery handles the nonlinear gradient terms. Fourth, this framework is applied to a Poisson observation model, in which a latent variable z modulates input firing rates, and the resulting energy function is plugged into the membrane potential equation; several rate kernels (exponential, linear, logistic) and noise models are worked through in turn. The HD circuit derivation then introduces angular coordinates, moves to 2D Cartesian to handle the wrapping problem, imposes a soft circular constraint, and derives the SCN dynamics for this system — but apparently using the standard (eqn. 31) rather than multiplicative SCN update equations. The mSCN development (parts 2 through 4) appears not to be used by the HD circuit application, which raises the question of whether this material belongs in the methods of this paper at all, or whether it represents a separate technical contribution that would be better suited to its own treatment elsewhere.

Minor:

Statements like “mirror recent experimental observations during head-direction realignment in mammals” need a reference (Fig. 3 caption).

Minor inconsistency in notation: Equations 7 through 9 use both boldface and a subscript for variables v and T, which I understood to be Nx1 vectors. Either v or v_i, but not both.

Reviewer #2: This is an interesting paper that examines how attractor dynamics and uncertainty representations can be multiplexed in a single spiking neural network, with a specific focus on the head direction system. It is clearly written and relevant to understanding probabilistic computations in attractor systems. The idea that observed variability may be not “just noise” that degrades an attractor, but functional, is also very interesting from a theoretical perspective. I appreciate the testable experimental predictions.

Some recommendations and questions:

- It seems that the main claim of the paper is that the spiking variability represents a posterior, i.e. is performing Bayesian inference over the heading angle. However, the main figures did not show this very clearly. It would be very helpful to see an overlay of the true posterior and that represented by the network, and perhaps a small analysis of parameter regimes in which the network representation is more or less accurate.

- The relationship between the network connectivity and its function are not entirely clear. It is mentioned that an established procedure was used to embed specific Langevin dynamics aligned to the ring manifold into the network, but it would be helpful to describe this in more detail in the main text. Additionally, does the connectivity end up with a ring-like topology, as in e.g. the fly HD system? Any additional analysis or intuition behind how the resulting connectivity patterns support the functions claimed in the rest of the manuscript would be very appreciated.

- If I understand correctly, this particular spiking network follows from the line of balanced spiking networks originating with Boerlin, Machens, and Deneve 2013. These networks are certainly quite interesting, but given that they are a bit non-traditional, a short overview of how they work in the main text would be quite useful.

- In general I think it would be very helpful to include the key equations in the main text. For example, the neuron and synaptic dynamics, network connectivity, where different noise sources enter, and how different features of inputs, activity, etc relate to the Bayesian quantities involved.

- In the absence of visual cues, is there a trade-off between the uncertainty in the representation and the stability of the attractor? Intuitively it seems larger uncertainty would correspond to more noise and in turn more drift of the bump, whereas if one dialed down this noise one could perhaps attain a more stable estimate but at the cost of a poorer encoding of uncertainty.

- I also didn’t fully understand whether one should think of the attractor and the uncertainty representation as “co-existing” vs “working together” somehow. A line or two explaining some intuition about this would help.

- Given that this is a spiking network, a short discussion of the relationship of the spiking statistics in the network model (e.g. firing rates) vs real spiking statistics in HD systems would be nice (if I am not mistaken, the EPG neurons spike in the fly…?).

- Some other literature in which spiking variability does not just simply degrade attractors but plays some role in supporting them, that could be worth citing: Mongillo, Hansel, van Vreeswijk PRL 2012; and Pang PLoS CB 2025.

Reviewer #3: This is an interesting paper that brings together the neurocomputational principles of sampling-based probabilistic inference and attractor dynamics, as far as I am aware, in a novel way. It applies this to the neural task of tracking head direction (HD) from uncertain sensory cues (angular velocity and fixed landmark inputs). This is useful (as a ‘case study’) for illustrating the properties of the unified system, but somewhat less convincing as reinterpretation of HD circuits in biology, as it was not always clear what properties of these circuits it could newly account for, compared to alternative/existing models. Perhaps that is not the main claim the paper intends to make, in which case it could perhaps use some revision to avoid this misinterpretation of the core aim.

More explicitly, it seems self-evident that “precision of HD representations...depends on the reliability of sensory cues” (line 10) but this does not seem sufficient alone to motivate modelling HD as an “uncertainty aware” circuit. There is no discussion in the paper of how this might augment downstream processing of HD information, for example, or why sampling-based inference “might be necessary” (line 43) or have some functional role in navigation. It is reasonable observation that attractor networks (including models of HD) have mostly treated noise as perturbation, whereas it could carry useful information; and legitimate to explore how this could be the case. However, the work presented is highly speculative (e.g. at no point based on anatomical data), and the results not fully convincing about the advantages of this specific approach to explaining HD circuit function.

For example, line 96 onwards summarises how the model produces “intuitive and biologically relevant behavior, as well as several experimentally observed phenomena”: a bump of activity, that tracks head direction well for reliable velocity inputs, with decreasing precision and accumulating error for noisy inputs, etc. I don’t see how any of these are novel to this model, compared to previous models. Is there anything in these results that allows us to distinguish the observed phenomena from “noise superimposed on a deterministic attractor” (line 106)? The same caveat applies to the reset results, i.e., these also seem comparable to previous models.

Another limitation is that the consequences of the particular assumptions made to set up the network (and relate it to biological features) were not always sufficiently explored to help the reader understand which aspects were actually crucial to its function. For example, I understand it to be theoretically motivated, by the choice of sampling approach, that “the sampling process requires neurons to share structured sources of stochastic fluctuation” in a specific way, but is it actually necessary for the circuit to function for the HD task? And if this was not observed (e.g. in the fly) to be implemented in correlated voltage fluctuations (their prediction 1, line 139 on) then could it still be supposed to be implemented in some other form? Is the use of Langevin sampling critical?

Despite the above issues, the paper does make significant contributions in developing, in a principled fashion, a spiking network that should maintain the best estimate of dynamic variable, assuming Poisson encoding and implementing Langevin sampling, constrained by a prior towards a (ring) attractor. A significant strength of the paper is that the methods work this out analytically for a variety of cases, e.g. different firing rate kernels, different noise models, linear or non-linear intrinsic dynamics, etc. As mentioned at the start of my review, this seems a very interesting and novel approach, with potential for wide application, even if its applicability to the HD system is not fully convincing.

Some additional comments:

Fig 1 doesn’t seem to quite connect together the various illustrated ideas because it does not show (in d) how the ‘bump’ behaviour actually reflects/encodes the uncertainty of the inferred distribution (through rapid fluctuations)

Figure 2, the caption (b) seems to be the only place in the main text that explains how the circular prior is set up. Overall, I think the main text explanation of the methods needs to be expanded. Around line 83 it becomes confusing, the paragraph seems to start concerning sampling only, but then describes the whole dynamics including the circular manifold. Are the slow interactions implementing both the following the slope of the log distribution and the circle constraint?

Fig2d, this seems to give the impression that the circuit has encoded uncertainty in the deviation from the true angle, but of course this is not known to the circuit (it does not know the true angle). But is it intended that there is some mechanism by which the neural system can extract and use the uncertainty as represented in the rapid fluctuations (2e)? This relates to prediction 3, around line 192, suggesting “motion of the bump reflects variability” not just the mean of the input. It was not entirely clear to me what observation would be expected under the alternative, e.g., that (at high resolution) it would be seen that the quality of the bump degrades, vs. the bump shape being maintained but fluctuating more in position. Or if the model output “reduces to integrating the posterior mean” how would the difference be detected?

Fig 3c I am not sure what I should examine in this figure to observe “excursions away from the circular manifold”.

Prediction 4, assymmetry and skew seems a counterintuitive prediction, but could be better explained, and is it not predicted in models that do not sampling based inference (4D)? In general it feels like the paper could be improved if the results could be compared to the most similar possible model that does not preserve the variance.

**********

**Have the authors made all data and (if applicable) computational code underlying the findings in their manuscript fully available?**

The PLOS Data policy requires authors to make all data and code underlying the findings described in their manuscript fully available without restriction, with rare exception (please refer to the Data Availability Statement in the manuscript PDF file). The data and code should be provided as part of the manuscript or its supporting information, or deposited to a public repository. For example, in addition to summary statistics, the data points behind means, medians and variance measures should be available. If there are restrictions on publicly sharing data or code —e.g. participant privacy or use of data from a third party—those must be specified.

Reviewer #1: Yes

Reviewer #2: Yes

Reviewer #3: None

**********

PLOS authors have the option to publish the peer review history of their article (what does this mean?). If published, this will include your full peer review and any attached files.

If you choose “no”, your identity will remain anonymous but your review may still be made public.

**Do you want your identity to be public for this peer review?**  For information about this choice, including consent withdrawal, please see our Privacy Policy.

Reviewer #1: No

Reviewer #2: No

Reviewer #3: No

**Figure resubmission:**

While revising your submission, we strongly recommend that you use PLOS’s NAAS tool (https://ngplosjournals.pagemajik.ai/artanalysis) to test your figure files. NAAS can convert your figure files to the TIFF file type and meet basic requirements (such as print size, resolution), or provide you with a report on issues that do not meet our requirements and that NAAS cannot fix.

After uploading your figures to PLOS’s NAAS tool - https://ngplosjournals.pagemajik.ai/artanalysis, NAAS will process the files provided and display the results in the "Uploaded Files" section of the page as the processing is complete. If the uploaded figures meet our requirements (or NAAS is able to fix the files to meet our requirements), the figure will be marked as "fixed" above. If NAAS is unable to fix the files, a red "failed" label will appear above. When NAAS has confirmed that the figure files meet our requirements, please download the file via the download option, and include these NAAS processed figure files when submitting your revised manuscript.

**Reproducibility:**

To enhance the reproducibility of your results, we recommend that authors of applicable studies deposit laboratory protocols in protocols.io, where a protocol can be assigned its own identifier (DOI) such that it can be cited independently in the future. Additionally, PLOS ONE offers an option to publish peer-reviewed clinical study protocols. Read more information on sharing protocols at https://plos.org/protocols?utm_medium=editorial-email&utm_source=authorletters&utm_campaign=protocols

# Author comment: Author response to Decision Letter 1

**DOI:** 10.1371/journal.pcbi.1014577.r002

11 Jun 2026

Attachment Submitted filename: response_to_reviewers.pdf

# Peer review: Decision Letter 1

**DOI:** 10.1371/journal.pcbi.1014577.r003

**Authors:** Daniel Bush, William Redman

14 Jul 2026

Dear Dr. Keemink,

We are pleased to inform you that your manuscript 'Combining sampling and attractor dynamics in spiking models of head direction systems' has been provisionally accepted for publication in PLOS Computational Biology.

Before your manuscript can be formally accepted you will need to complete some formatting changes, which you will receive in a follow up email. A member of our team will be in touch with a set of requests.

Please note that your manuscript will not be scheduled for publication until you have made the required changes, so a swift response is appreciated.

IMPORTANT: The editorial review process is now complete. PLOS will only permit corrections to spelling, formatting or significant scientific errors from this point onwards. Requests for major changes, or any which affect the scientific understanding of your work, will cause delays to the publication date of your manuscript.

Should you, your institution's press office or the journal office choose to press release your paper, you will automatically be opted out of early publication. We ask that you notify us now if you or your institution is planning to press release the article. All press must be co-ordinated with PLOS.

Thank you again for supporting Open Access publishing; we are looking forward to publishing your work in PLOS Computational Biology.

Best regards,

William Redman

Academic Editor

PLOS Computational Biology

Daniel Bush

Section Editor

PLOS Computational Biology

***********************************************************

All 3 reviewers agree that the authors made changes during the revision that addressed their concerns and view this submission as now ready to be accepted. I am in agreement.

One minor thing to note, Reviewer 3 has said "I found it somewhat difficult to follow how the conceptual development relates to the effective (mechanistic) implementation as a spiking neural circuit. The authors might want to consider whether this could be improved to help neuroscientists appreciate the contribution and how it could be experimentally explored." The authors might consider this point as they ready the final version of the manuscript.

Reviewer's Responses to Questions

**Comments to the Authors:**

**Please note here if the review is uploaded as an attachment.**

Reviewer #1: I thank the authors for the substantial revision. The reorganized Methods are much clearer, and Box 1 and the key equations in the main text are a real improvement, and the notational fixes and clarification of "standard" Langevin dynamics are all fine. The multimodal integration figure is clear to me now, and I agree that the dynamic encoding of reliability is beyond the scope of the current paper.

Reviewer #2: I thank the authors for their detailed revision, which has addressed all of my concerns. The revised manuscript is much clearer, in particular the exposition of the modeling.

Reviewer #3: I am generally happy with the authors' responses and revision of the paper, which has helpfully clarified the scope and contributions. I found it somewhat difficult to follow how the conceptual development relates to the effective (mechanistic) implementation as a spiking neural circuit. The authors might want to consider whether this could be improved to help neuroscientists appreciate the contribution and how it could be experimentally explored.

**********

**Have the authors made all data and (if applicable) computational code underlying the findings in their manuscript fully available?**

The PLOS Data policy requires authors to make all data and code underlying the findings described in their manuscript fully available without restriction, with rare exception (please refer to the Data Availability Statement in the manuscript PDF file). The data and code should be provided as part of the manuscript or its supporting information, or deposited to a public repository. For example, in addition to summary statistics, the data points behind means, medians and variance measures should be available. If there are restrictions on publicly sharing data or code —e.g. participant privacy or use of data from a third party—those must be specified.

Reviewer #1: Yes

Reviewer #2: Yes

Reviewer #3: Yes

**********

PLOS authors have the option to publish the peer review history of their article (what does this mean?). If published, this will include your full peer review and any attached files.

If you choose “no”, your identity will remain anonymous but your review may still be made public.

**Do you want your identity to be public for this peer review?**  For information about this choice, including consent withdrawal, please see our Privacy Policy.

Reviewer #1: No

Reviewer #2: No

Reviewer #3: No

# Editor report: Acceptance letter

**DOI:** 10.1371/journal.pcbi.1014577.r004

**Authors:** Daniel Bush, William Redman

PCOMPBIOL-D-26-00420R1

Combining sampling and attractor dynamics in spiking models of head direction systems

Dear Dr Keemink,

I am pleased to inform you that your manuscript has been formally accepted for publication in PLOS Computational Biology. Your manuscript is now with our production department and you will be notified of the publication date in due course.

The corresponding author will soon be receiving a typeset proof for review, to ensure errors have not been introduced during production. Please review the PDF proof of your manuscript carefully, as this is the last chance to correct any errors. Please note that major changes, or those which affect the scientific understanding of the work, will likely cause delays to the publication date of your manuscript.

Soon after your final files are uploaded, unless you have opted out, the early version of your manuscript will be published online. The date of the early version will be your article's publication date. The final article will be published to the same URL, and all versions of the paper will be accessible to readers.

For Research, Software, and Methods articles, you will receive an invoice from PLOS for your publication fee after your manuscript has reached the completed accept phase. If you receive an email requesting payment before acceptance or for any other service, this may be a phishing scheme. Learn how to identify phishing emails and protect your accounts at https://explore.plos.org/phishing.

Thank you again for supporting PLOS Computational Biology and open-access publishing. We are looking forward to publishing your work!

With kind regards,

Janani Seenivasan

PLOS Computational Biology | Carlyle House, Carlyle Road, Cambridge CB4 3DN | United Kingdom ploscompbiol@plos.org | Phone +44 (0) 1223-442824 | ploscompbiol.org | @PLOSCompBiol
